# Identification of MKRN1 as a key modulator of the p53-MDM2 feedback loop

**DOI:** 10.1038/s41418-026-01662-4

**Published:** 2026-01-30

**Authors:** Tatsuya Shimada, Takuya Noguchi, Ryuto Komatsu, Kohei Otani, Takaya Komatsu, Sara Suzuki, Maki Mitsuya, Takumi Okubo, Ryo Ito, Mayuka Yamada, Yusuke Hirata, Atsushi Matsuzawa

**Affiliations:** 1 Laboratory of Health Chemistry, Graduate School of Pharmaceutical Sciences, Tohoku University, Sendai, Japan; 2 Department of Medical Biochemistry, School of Pharmacy, Iwate Medical University, Yahaba, Japan

**Keywords:** Cell biology, Kinases

## Abstract

The p53-murine double minute 2 (MDM2) feedback loop plays a central role in tumor suppression by optimizing p53-dependent DNA damage responses (DDRs), though it has been suggested that factors other than MDM2 are also involved in the regulation of the p53-MDM2 feedback loop. We identified makorin ring finger protein 1 (MKRN1) as a novel ubiquitin E3 ligase that ubiquitinates MDM2 and thereby promotes the p53 activation. As previously demonstrated, MKRN1 ubiquitinates and degrades p53 under steady-state conditions. However, when DNA damage occurs, MKRN1 switches its substrate to MDM2. Thereafter, MKRN1 promotes the stabilization and activation of p53 through proteasomal degradation of MDM2, which contributes to the elimination of DNA-damaged cells. Moreover, we found that the switch in the substrate of MKRN1 was determined by the NAD(+)-dependent protein deacetylase Sirtuin-1 (SIRT1). Thus, our results suggest that MKRN1 working in conjunction with SIRT1 is a master regulator of the p53-MDM2 feedback loop modulated by crosstalk between ubiquitination and acetylation.

## Main

DDRs are important mechanisms for avoiding the accumulation of DNA damage, which allows cancer development through mutagenic consequences [1–3]. p53, one of the most prominent tumor suppressor genes, plays a crucial role in DDRs [1, 4–7]. At a steady state, p53 levels are negatively regulated by MDM2-induced proteasomal degradation. However, upon DNA damage, post-translational modifications (PTMs) dissociate MDM2 from p53, leading to the stabilization and activation of p53 [8, 9]. Indeed, in p53-deficient mice, tumors develop spontaneously, although the developmental process is otherwise normal [10, 11]. Thus, MDM2 degradation, which activates p53, plays an important role in the anti-tumor activity of p53. Under DNA damage conditions, MDM2 degradation is thought to be mediated by self-ubiquitination [12, 13]. However, recent studies have demonstrated that the E3 ligase activity of MDM2 is not necessarily required for its degradation during DNA damage [14]. Several ubiquitin ligases, such as the p300-CBP-associated factor (PCAF) and β-TRCP, have been identified as the ubiquitin ligases that regulate the expression level of MDM2 [15–18].

MKRN1 has been identified as a RING finger ubiquitinating enzyme responsible for ubiquitination and proteasome-dependent degradation of human telomerase reverse transcriptase (hTERT) [19]. Several molecules including p53, p21, Fas-associated protein with death domain (FADD), Phosphatase and Tensin Homolog (PTEN), and AMP-activated protein kinase (AMPK) have been identified as substrates of MKRN1 [20–23]. Thus, MKRN1 functions as a key regulator of various biological processes, including cell cycle, apoptosis, and cellular metabolism [20, 23–25]. However, the involvement of MKRN1 in the DDRs remains unclear.

Lysine acetylation is a major post-translational modification of proteins that modulates their biological functions [26, 27]. This process is reciprocally regulated by lysine acetyltransferases and lysine deacetylases, which catalyze the addition or removal of acetyl groups from their targets, respectively [28, 29]. Acetylation of non-histone proteins is thought to modulate protein-protein or protein-nucleic acid interactions by altering protein conformation, stability, hydrophobicity, and localization [28, 29]. Notably, p53 acetylation inhibits its binding to MDM2, leading to p53 stabilization and activation [30]. In contrast, the DNA damage-dependent deacetylation of MDM2 promotes self-ubiquitination [31]. Thus, reversible lysine modification by acetyltransferases and deacetylases serves as a molecular switch that regulates cellular functions [28, 29]. SIRT1 is a class III histone deacetylase (HDAC) that catalyzes the deacetylation of its substrates in an NAD(+)-dependent manner [32]. Accumulating evidence shows that SIRT1 deacetylates not only histone proteins but also a wide variety of non-histone proteins such as the nuclear factor-κB subunit RelA/p65, mammalian target of rapamycin (mTOR), nuclear factor erythroid 2-related factor 2 (Nrf2), and Forkhead box protein O1 (FoxO1), and plays key roles in gene regulation, apoptosis, senescence, cell growth, aging, and tumorigenesis [33–35].

In the present study, we revealed that MKRN1 functions as a ubiquitinating enzyme responsible for the MDM2 degradation in response to DNA damage. MKRN1 binds to MDM2 deacetylated by SIRT1 in a DNA damage-dependent manner, and then leads MDM2 to the proteasomal degradation, resulting in the stabilization and activation of p53, and subsequent apoptosis. Together with previous studies showing that MKRN1 functions as a ubiquitin ligase responsible for p53 under steady-state conditions, MKRN1 has emerged as a central player in modulating the p53-MDM2 feedback loop.

## Results

### MKRN1 promotes DNA damage-induced p53 stabilization and apoptosis

**Fig. 1:**
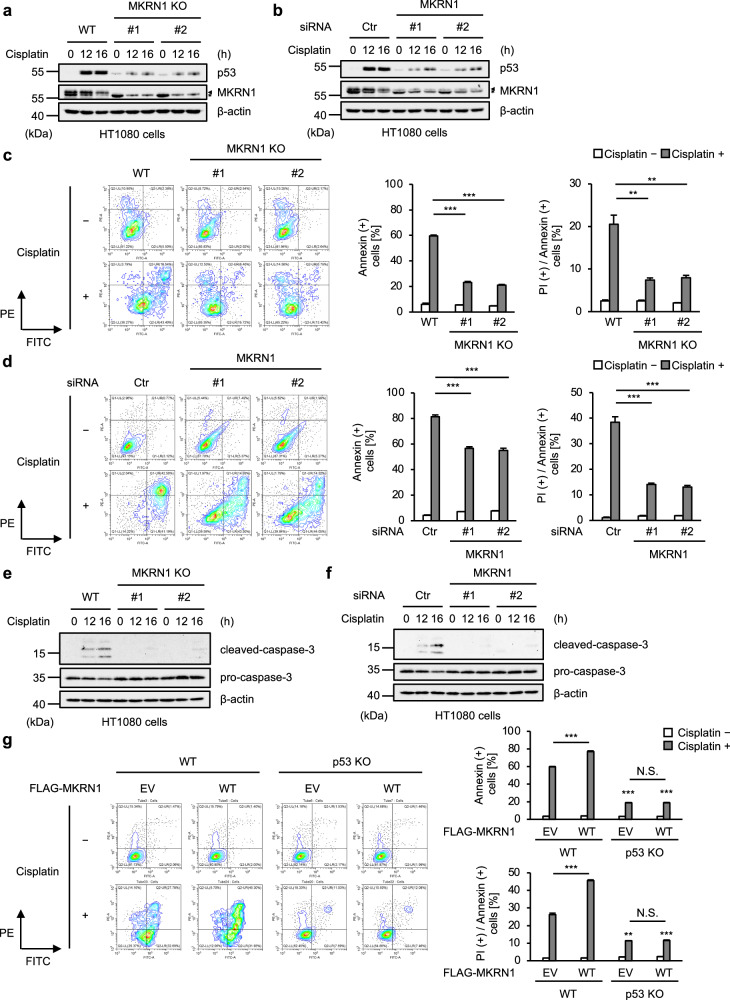
MKRN1 promotes DNA damage-induced p53 stabilization and apoptosis. **a** HT1080 cells were treated with cisplatin (25 μM) for the indicated periods. Cell lysates were subjected to immunoblotting with the indicated antibodies. The band indicated by the asterisk is non-specific bands. **b** HT1080 cells were transfected with siRNA for negative control or MKRN1 (MKRN1 #1 or MKRN1 #2). After 48 h, cells were treated with cisplatin (25 μM) for the indicated periods. Cell lysates were subjected to immunoblotting with the indicated antibodies. The band indicated by the asterisk is non-specific bands. **c** HT1080 cells were treated with cisplatin (25 μM) for 24 h. Apoptotic cells were labeled with annexin V-FITC and PI for 15 min and analyzed by FACS. The data were converted to FITC-PE fluorescence density plots and quantified. Data shown are the mean ± S.D. (*n* = 3). Significant differences were determined by one-way ANOVA, followed by Tukey-Kramer test; *** *p* < 0.001, ** *p* < 0.01. **d** HT1080 cells were transfected with siRNA for negative control or MKRN1 (MKRN1 #1 or MKRN1 #2). After 48 h, cells were treated with cisplatin (25 μM) for 24 h. Apoptotic cells were labeled with annexin V-FITC and PI for 15 min and analyzed by FACS. The data were converted to FITC-PE fluorescence density plots and quantified. Data shown are the mean ± S.D. (n = 3). Significant differences were determined by one-way ANOVA, followed by Tukey-Kramer test; *** *p* < 0.001. **e** HT1080 cells were treated with cisplatin (25 μM) for the indicated periods. Cell lysates were subjected to immunoblotting with the indicated antibodies. **f** HT1080 cells were transfected with siRNA for negative control or MKRN1 (MKRN1 #1 or MKRN1 #2). After 48 h, cells were treated with cisplatin (25 μM) for the indicated periods. Cell lysates were subjected to immunoblotting with the indicated antibodies. **g** HT1080 cells were transfected with the indicated plasmids for 24 h and then treated with cisplatin (25 μM) for 24 h. Apoptotic cells were labeled with annexin V-FITC and PI for 15 min and analyzed by FACS. The data were converted to FITC-PE fluorescence density plots and quantified. Data shown are the mean ± S.D. (*n* = 3). Significant differences were determined by one-way ANOVA, followed by Tukey-Kramer test; *** *p* < 0.001, ** *p* < 0.01, N.S.: not significant.

A previous study has demonstrated that MKRN1 degrades p53 through the ubiquitin-proteasome system [20]. As shown in Extended Data Fig. 1a, under unstimulated conditions, p53 expression increased when MKRN1 was deleted by using the Clustered Regularly Interspaced Short Palindromic Repeat/CRISPR-associated protein-9 nuclease (CRISPR/Cas9) system in human fibrosarcoma HT1080 cells. However, no difference in the p53 expression levels was observed in the presence of the proteasome inhibitor MG132, suggesting that proteasomal degradation of p53 is suppressed in MKRN1 knockout (KO) HT1080 cells (Extended Data Fig. 1b). Similar observations were made when MKRN1 was knocked down by siRNA (Extended Data Fig. 1c, d). Additionally, it turned out that endogenous MKRN1 binds to endogenous p53 at steady state, and MKRN1 KO HT1080 cells showed increased levels of K48-ubiquitinated p53, an indicator of proteasomal degradation (Extended Data Fig. 1e, f). On the other hand, the mRNA levels of p53 at steady state were not changed by MKRN1 knockout (Extended Data Fig. 1g). Collectively, these results suggest that MKRN1 promotes the proteasomal degradation of p53 under steady-state conditions in HT1080 cells. We next investigated the role of MKRN1 in p53 stabilization upon DNA damage, and found that the p53 stabilization induced by the DNA-damaging agent cisplatin was attenuated in both MKRN1 KO HT1080 cells and MKRN1 knockdown HT1080 cells (Fig. 1a, and 1b). Similar results were observed in the presence of other DNA-damaging agents such as doxorubicin and etoposide (Extended Data Fig. 2a, b). Moreover, reduced p53 stabilization was observed in A549 and U2OS cells (Extended Data Fig. 2c and 2d). Collectively, these results suggest that MKRN1 is required for the p53 stabilization in response to DNA damage. Our previous studies have demonstrated that cisplatin treatment under these experimental conditions induces p53-dependent apoptosis in HT1080 cells [36, 37]. Therefore, we investigated the effect of the MKRN1 knockout on p53-dependent apoptosis. As shown in Fig. 1c, quantitative analysis by using fluorescence-activated cell sorting (FACS) after staining with annexin V-FITC and propidium iodide (PI) demonstrated that annexin V-positive (apoptotic) cells were significantly reduced in MKRN1 KO HT1080 cells compared to wild-type (WT) HT1080 cells. Similar results were observed when MKRN1 was knocked down (Fig. 1d). Consistent with these observations, cisplatin-induced caspase activation was attenuated in MKRN1 KO or knockdown HT1080 cells (Fig. 1e, f). Additionally, the finding that MKRN1 KO HT1080 cells exhibited resistance to cisplatin-induced apoptosis was supported by the cell viability assays (Extended Data Fig. 2e, h). MKRN1 KO or knockdown HT1080 cells exhibited higher cell viability in the presence of doxorubicin or etoposide (Extended Data Fig. 2i, j). Cell viability in the presence of cisplatin was also restored in A549 and U2OS cells following MKRN1 knockdown (Extended Data Fig. 2k, l). Moreover, in MKRN1 KO HT1080 cells, cisplatin treatment reduced the mRNA levels of BAX, PUMA, and NOXA, which contribute to p53-dependent apoptosis (Extended Data Fig. 3a–c). We thus examined whether MKRN1 specifically promotes p53-induced apoptosis. In WT HT1080 cells, exogenously overexpressed MKRN1 increased in both Annexin V-positive and PI/Annexin V-double positive cells induced by cisplatin, whereas it failed to do so in HT1080 p53 KO cells, suggesting that MKRN1 promotes p53-dependent apoptosis (Fig. 1g and Extended Data Fig. 3d). On the other hand, comet assays revealed that the degree of cisplatin-induced DNA damage remained unchanged in MKRN1 KO HT1080 cells, meaning that MKRN1 does not affect cisplatin-dependent DNA damage, but enhances cisplatin-induced apoptosis by regulating downstream of DNA damage (Extended Data Fig. 4). Taken together, these results indicate that MKRN1 positively regulates DNA damage-induced apoptosis through the stabilization of p53.

**Fig. 2:**
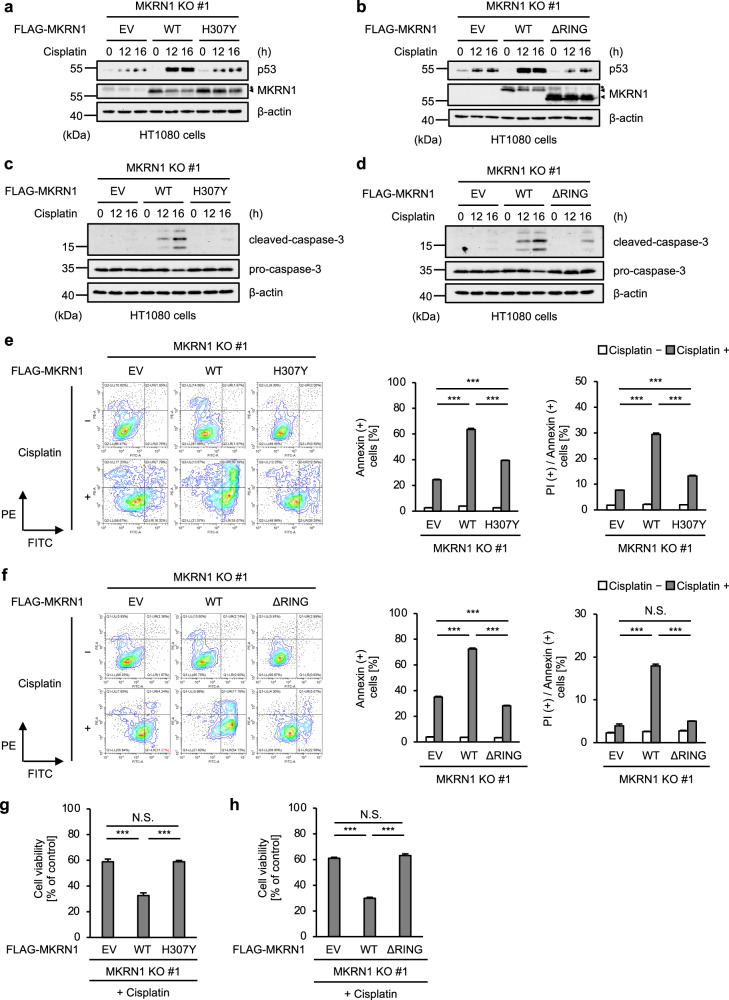
The E3 ubiquitin ligase activity of MKRN1 is required for the p53 stabilization and apoptosis. **a**–**d** HT1080 cells were treated with cisplatin (25 μM) for the indicated periods. Cell lysates were subjected to immunoblotting with the indicated antibodies. The band indicated by the asterisk is non-specific bands. **e**,**f** HT1080 cells were treated with cisplatin (25 μM) for 24 h. Apoptotic cells were labeled with annexin V-FITC and PI for 15 min and analyzed by FACS. The data were converted to FITC-PE fluorescence density plots and quantified. Data shown are the mean ± S.D. (n = 3). Significant differences were determined by one-way ANOVA, followed by Tukey-Kramer test; *** *p* < 0.001, N.S.: not significant. **g**,**h** HT1080 cells were treated with cisplatin (25 μM) for 24 h and then subjected to Cell Proliferation Assay. Data shown are the mean ± S.D. (*n* = 3). Significant differences were determined by one-way ANOVA, followed by Tukey-Kramer test; *** *p* < 0.001, N.S.: not significant.

**Fig. 3:**
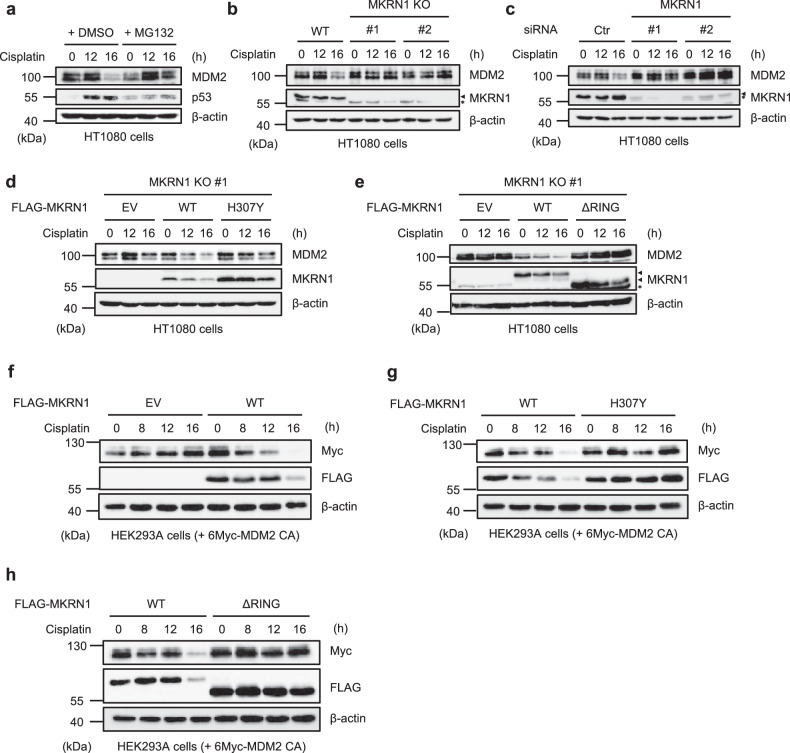
MKRN1 promotes the MDM2 degradation in response to DNA damage. **a** HT1080 cells were treated with cisplatin (25 μM) for the indicated periods in the presence of DMSO or MG132 (5 μM). Cell lysates were subjected to immunoblotting with the indicated antibodies. **b** HT1080 cells were treated with cisplatin (25 μM) for the indicated periods. Cell lysates were subjected to immunoblotting with the indicated antibodies. The band indicated by the asterisk is non-specific bands. **c** HT1080 cells were transfected with siRNA for negative control or MKRN1 (MKRN1 #1 or MKRN1 #2). After 48 h, HT1080 cells were treated with cisplatin (25 μM) for the indicated periods. Cell lysates were subjected to immunoblotting with the indicated antibodies. The band indicated by the asterisk is non-specific bands. **d**,**e** HT1080 cells were treated with cisplatin (25 μM) for the indicated periods. Cell lysates were subjected to immunoblotting with the indicated antibodies. The band indicated by the asterisk is non-specific bands. **f**–**h** HEK293A cells were transfected with the indicated plasmids for 24 h and then treated with cisplatin (25 μM) for the indicated periods. Cell lysates were subjected to immunoblotting with the indicated antibodies.

**Fig. 4:**
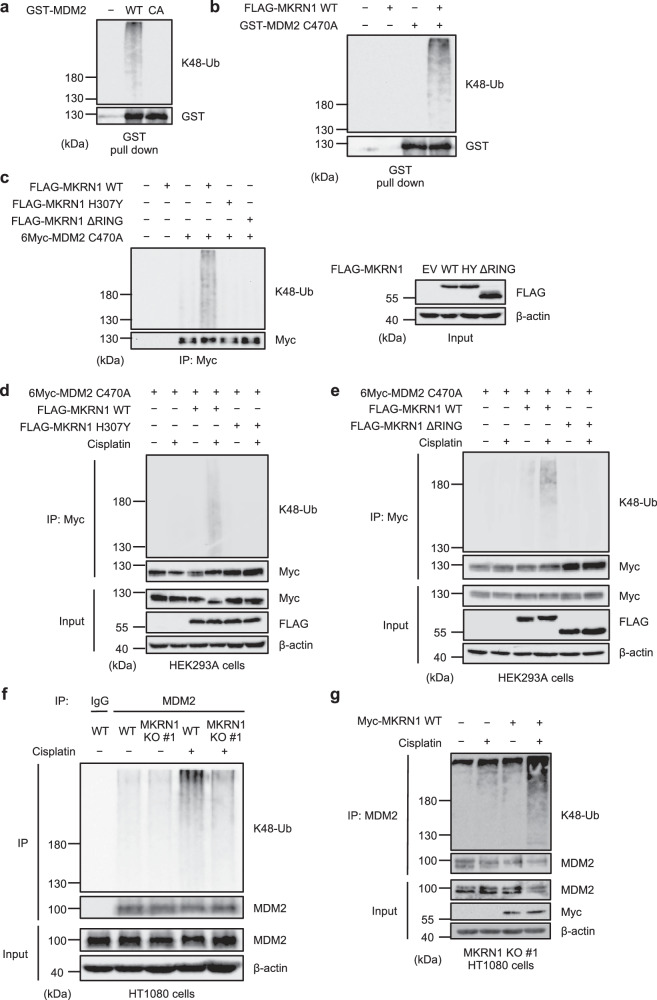
MKRN1 ubiquitinates MDM2 in response to DNA damage. **a** In vitro ubiquitination reactions were performed using GST-MDM2 WT or C470A recombinant proteins and then subjected to immunoblotting with the indicated antibodies. **b** In vitro ubiquitination reactions were performed using GST-MDM2 C470A recombinant proteins and affinity-purified FLAG-MKRN1 WT and then subjected to immunoblotting with the indicated antibodies. **c** In vitro ubiquitination reactions were performed using affinity-purified 6Myc-MDM2 C470A and FLAG-MKRN1 WT or mutants (MKRN1 H307Y and ΔRING), and then subjected to immunoblotting with the indicated antibodies. **d**,**e** HEK293A cells were transfected with the indicated plasmids for 24 h and then treated with cisplatin (25 μM) for 8 h in the presence of MG132 (5 μM). Cell lysates were immunoprecipitated with anti-Myc agarose beads and then subjected to immunoblotting with the indicated antibodies. **f** HT1080 cells were treated with cisplatin (25 μM) for 8 h in the presence of MG132 (5 μM). Cell lysates were immunoprecipitated with anti-IgG or MDM2 antibody and then subjected to immunoblotting with the indicated antibodies. **g** MKRN1 KO HT1080 cells were transfected with the indicated plasmids for 24 h and then treated with cisplatin (25 μM) for 8 h in the presence of MG132 (5 μM). Cell lysates were immunoprecipitated with anti-MDM2 antibody and then subjected to immunoblotting with the indicated antibodies.

### The E3 ubiquitin ligase activity of MKRN1 is required for the p53 stabilization and apoptosis

Next, we examined the requirement of the E3 ubiquitin ligase activity of MKRN1 for DNA damage-induced p53 stabilization and apoptosis. To this end, we established MKRN1-reconstituted HT1080 cell lines. The reconstitution of MKRN1 wild-type (WT) in MKRN1 KO HT1080 cells successfully restored the stabilization of p53, whereas reconstitution of an enzymatically inactive mutant of MKRN1 (H307Y mutant), in which histidine (H) 307 was substituted with tyrosine (Y), failed (Fig. 2a). An MKRN1 deletion mutant lacking the RING domain (MKRN1 ΔRING mutant), which is required for its E3 ubiquitin ligase activity, also failed to restore the stabilization of p53 (Fig. 2b). Moreover, the reconstitution of MKRN1 WT, but not the H307Y or ΔRING mutants restored cisplatin-induced caspase-3 activation (Fig. 2c, d). Consistent with these observations, cisplatin-induced apoptosis was not sensitized by the reconstitution of the MKRN1 H307Y and ΔRING mutants (Fig. 2e, f). The observations that MKRN1 H307Y and ΔRING mutants could not induce apoptosis were supported by the cell viability assays (Fig. 2g, h, Extended Data Fig. 5a, b). These results therefore suggest that the E3 ubiquitin ligase activity of MKRN1 is required for DNA damage-induced apoptosis.

**Fig. 5:**
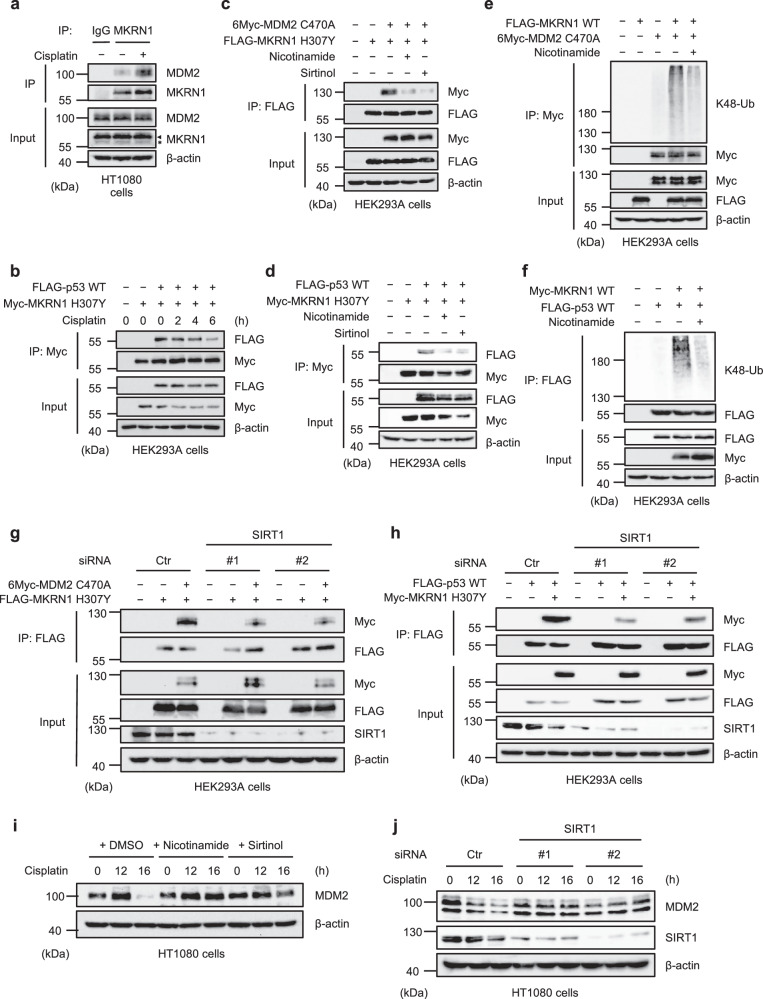
SIRT1 is required for the MDM2 ubiquitination by MKRN1. **a** HT1080 cells were treated with cisplatin (25 μM) for 8 h. Cell lysates were immunoprecipitated with anti-IgG or MKRN1 antibody and then subjected to immunoblotting with the indicated antibodies. The band indicated by the asterisk is non-specific bands. **b** HEK293A cells were transfected with the indicated plasmids for 24 h and then treated with cisplatin (25 μM) for the indicated periods. Cell lysates were immunoprecipitated with anti-Myc agarose beads and then subjected to immunoblotting with the indicated antibodies. **c** HEK293A cells were transfected with the indicated plasmids for 24 h and then treated with nicotinamide (1 mM) or sirtinol (10 μM) for 6 h. Cell lysates were immunoprecipitated with anti-FLAG agarose beads and then subjected to immunoblotting with the indicated antibodies. **d** HEK293A cells were transfected with the indicated plasmids for 24 h and then treated with nicotinamide (1 mM) or sirtinol (10 μM) for 6 h. Cell lysates were immunoprecipitated with anti-Myc agarose beads and then subjected to immunoblotting with the indicated antibodies. **e** HEK293A cells were transfected with the indicated plasmids for 24 h and then treated with MG132 (5 μM), and DMSO or nicotinamide (1 mM) for 6 h. Cell lysates were immunoprecipitated with anti-Myc agarose beads and then subjected to immunoblotting with the indicated antibodies. **f** HEK293A cells were transfected with the indicated plasmids for 24 h and then treated with MG132 (5 μM), and DMSO or nicotinamide (1 mM) for 6 h. Cell lysates were immunoprecipitated with anti-FLAG agarose beads and then subjected to immunoblotting with the indicated antibodies. **g** HEK293A cells were transfected with siRNA for negative control or SIRT1 (SIRT1 #1 or SIRT1 #2). After 24 h, cells were transfected with the indicated plasmids for 24 h. Cell lysates were immunoprecipitated with anti-FLAG agarose beads and then subjected to immunoblotting with the indicated antibodies. **h** HEK293A cells were transfected with siRNA for negative control or SIRT1 (SIRT1 #1 or SIRT1 #2). After 24 h, cells were transfected with the indicated plasmids for 24 h. Cell lysates were immunoprecipitated with anti-FLAG agarose beads and then subjected to immunoblotting with the indicated antibodies. **i** HT1080 cells were treated with cisplatin (25 μM) for the indicated periods in the presence of nicotinamide (1 mM) or sirtinol (10 μM). Cell lysates were subjected to immunoblotting with the indicated antibodies. **j** HT1080 cells were transfected with siRNA for negative control or SIRT1 (SIRT1 #1 or SIRT1 #2). After 24 h, cells were treated with cisplatin (25 μM) for the indicated periods. Cell lysates were subjected to immunoblotting with the indicated antibodies.

### MKRN1 promotes the MDM2 degradation in response to DNA damage

p53 undergoes downregulation by the ubiquitin E3 ligase MDM2 via ubiquitination-dependent proteasomal degradation. However, upon DNA damage, p53 is stabilized because MDM2 degradation is promoted [38]. Therefore, our subsequent investigation focused on determining the impact of MKRN1 on MDM2 degradation. As shown in Fig. 3a, cisplatin-induced MDM2 degradation at 16 h after the treatment was inhibited by the proteasome inhibitor MG132, confirming that DNA damage promotes the proteasomal degradation of MDM2. Cisplatin-induced MDM2 degradation was strongly inhibited in MKRN1 KO HT1080 cells (Fig. 3b). This observation was confirmed by the MKRN1 knockdown (Fig. 3c). Meanwhile, mRNA levels of MDM2 were not increased but rather suppressed in MKRN1 KO HT1080 cells upon cisplatin treatment, possibly due to the reduced p53 activation (Extended Data Fig. 6a). MKRN1 therefore appears to regulate the MDM2 expression at protein but not mRNA levels. As shown in Fig. 1a, the p53 stabilization was observed 12 h after cisplatin treatment in WT HT1080 cells, whereas the degradation of MDM2, which allows to stabilize p53, was observed at a later time point (16 h) (Fig. 3a–c). At first glance, this observation seems to contradict the kinetics of MDM2-mediated p53 degradation. However, the degradation of MDM2 was observed at earlier time points in WT HT1080 cells, under the condition in which transcriptional induction was blocked by the general protein synthesis inhibitor cycloheximide (CHX) (Extended Data Fig. 6b). This finding suggests the possibility that the degradation of MDM2 was observed with an apparent delay because MDM2 is transcriptionally upregulated by stabilized p53. In addition, it was found that the MDM2 degradation was suppressed in MKRN1 KO HT1080 cells, even when the effects of transcriptional upregulation of MDM2 were blocked by CHX (Extended Data Fig. 6b). On the other hand, the suppression of the MDM2 degradation induced by cisplatin was canceled by the reconstitution of MKRN1 WT, but not by the H307Y and ΔRING mutants (Fig. 3d, e). Moreover, exogenously overexpressed MKRN1 WT, but not the H307Y or ΔRING mutants promoted the MDM2 degradation at 16 h after cisplatin treatment (Fig. 3f–h). Collectively, these results suggest that MKRN1 enzymatically promotes the MDM2 degradation. Next, we investigated the link between the reduced p53 stabilization and the suppression of MDM2 degradation in MKRN1 KO HT1080 cells. When the expression of MDM2 was abrogated by knockdown in MKRN1 KO HT1080 cells, cisplatin-induced p53 stabilization was restored (Extended Data Fig. 6c). Moreover, in MDM2 knockdown HT1080 cells, cell viability after cisplatin treatment was not affected by the expression of MKRN1 (Extended Data Fig. 6d). Meanwhile, the p53 stabilization induced by Nutlin-3, which stabilized p53 independently of the MDM2 degradation, was not altered in the absence of MKRN1 (Extended Data Fig. 6e) [39, 40]. Collectively, these results suggest that MKRN1 promotes the p53 stabilization by promoting MDM2 degradation.

**Fig. 6:**
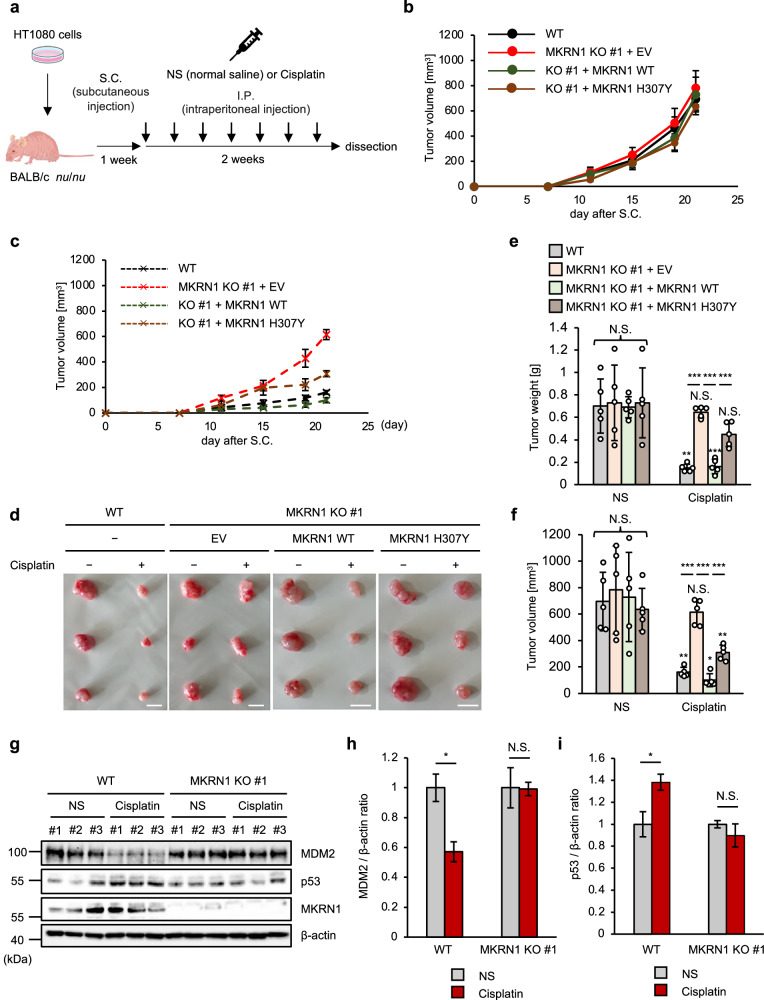
MKRN1 is a potential therapeutic target for cancer treatment. **a** HT1080 cells were subcutaneously injected into nude mice, and the xenografts were analyzed as described in the method section. (NS: the group administered normal saline, cisplatin: the group administered cisplatin). **b**,**c** Tumor volume was measured and is shown as the mean ± S.D. (WT: *n* = 5, MKRN1 KO + EV: *n* = 5, MKRN1 KO + MKRN1 WT: *n* = 5, MKRN1 KO + MKRN1 H307Y: *n* = 5). **d** A representative image of tumor xenografts harvested at day 27 (scale bar, 10 mm). **e**,**f** Tumor weight (**e**) and tumor volume (**f**) were measured at day 27. Data shown are the mean ± S.D. (WT: *n* = 5, MKRN1 KO + EV: *n* = 5, MKRN1 KO + MKRN1 WT: *n* = 5, MKRN1 KO + MKRN1 H307Y: *n* = 5). Statistical differences were determined by Student’s t-test; *** *p* < 0.001, ** *p* < 0.01, * *p* < 0.05, N.S.: not significant. **g** Tumor xenografts were subjected to immunoblotting with the indicated antibodies. **h**,**i** The blots in (**g**) were quantified. Data shown are mean ± S.D. (WT: *n* = 3, MKRN1 KO: *n* = 3). Statistical differences were determined by Student’s t-test; * *p* < 0.05 and N.S.: not significant.

### MKRN1 ubiquitinates MDM2 in response to DNA damage

Next, we examined whether MKRN1 ubiquitinates MDM2. Since both MKRN1 and MDM2 possess the E3 ubiquitin ligase activity, we had to remove MDM2’s E3 ubiquitin ligase activity to detect only MKRN1’s E3 ubiquitin ligase activity. As shown in Fig. 4a, the in vitro ubiquitination assay revealed that the MDM2 C470A mutant failed to induce self-ubiquitination. The MDM2 C470A mutant was ubiquitinated when incubated with MKRN1 WT, but not with the H307Y and ΔRING mutants in vitro (Fig. 4b, c). Similar results were obtained in cellular experiments. As shown in Fig. 4d, e, exogenously overexpressing MKRN1 WT, but not the H307Y and ΔRING mutants promoted the cisplatin-induced ubiquitination of MDM2 C470A. Taken together, these results indicate that MKRN1 ubiquitinates MDM2. Next, we examined whether MKRN1 ubiquitinates MDM2 in response to DNA damage. As shown in Fig. 4f, cisplatin treatment enhanced the ubiquitination of endogenous MDM2 in WT but not MKRN1 KO HT1080 cells. In addition, the reconstitution of MKRN1 WT into MKRN1 KO HT1080 cells successfully restored cisplatin-induced ubiquitination of endogenous MDM2 (Fig. 4g). Therefore, MKRN1 seems to play a role in the ubiquitination-mediated degradation of MDM2 in response to DNA damage. Next, we investigated the interactions between MKRN1 and MDM2. MKRN1 H307Y and MDM2 C470A mutants interacted with each other both in cells and in vitro (Extended Data Fig. 7a–c). The N- and C-terminal deletion mutants of MKRN1 could interact with the MDM2 C470A mutant, whereas the deletion mutant of RING domain of MKRN1 did not (Extended Data Fig. 7d–g). Collectively, these results suggest that MKRN1 interacts with MDM2 through its RING domain, and then directly ubiquitinates MDM2.

**Fig. 7:**
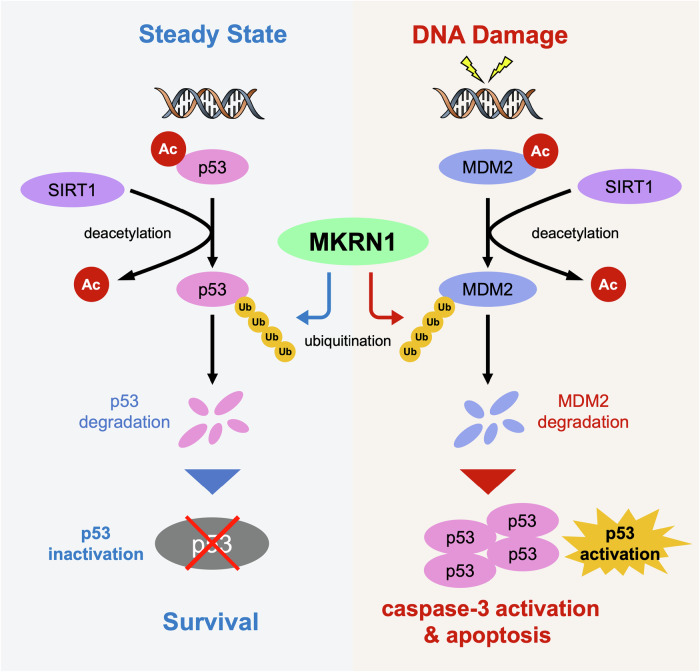
Schematic model to explain our study. Under steady-state conditions, MKRN1 ubiquitinates deacetylated p53 by SIRT1, resulting in proteasomal degradation of p53. Meanwhile, upon DNA damage, MKRN1 switches its substrate to MDM2 that was deacetylated by SIRT1 in response to DNA damage, leading to the p53 activation. Thus, the p53-MDM2 feedback loop is modulated by crosstalk between MKRN1 and SIRT1.

### SIRT1 is required for the MDM2 ubiquitination by MKRN1

The results shown in Fig. 1–4, it is suggested that MKRN1 switches its substrates in the presence or absence of DNA damage. We investigated the mechanisms underlying these effects. First, we observed that the interaction between MKRN1 and MDM2 was enhanced by the cisplatin treatment, even when evaluated at the endogenous level (Fig. 5a and Extended Data Fig. 8a). In contrast, cisplatin treatment suppressed the interaction between MKRN1 and p53 (Fig. 5b). We speculated that the reciprocal substrate selectivity of MKRN1 is determined by the acetylation status of p53 and MDM2 because its stability is regulated by their acetylation. In particular, deacetylation facilitates their degradation [31, 41]. Indeed, we found that inhibitors of the NAD(+)-dependent protein deacetylase SIRT1, such as nicotinamide and sirtinol, prevented the interaction of MKRN1 with MDM2 (Fig. 5c and Extended Data Fig. 8b). Meanwhile, the SIRT1 inhibitors also inhibited the interaction between MKRN1 and p53 (Fig. 5d and Extended Data Fig. 8b). Moreover, the SIRT1 inhibitors prevented the ubiquitination of p53 and MDM2 mediated by MKRN1 (Fig. 5e and 5f). These observations, therefore, suggest that deacetylation of p53 and MDM2 is required for their interaction with MKRN1 and subsequent ubiquitination. Next, we examined the involvement of SIRT1 in the MKRN1-dependent regulation of p53 and MDM2. In SIRT1 knockdown cells, both MKRN1-p53 and MKRN1-MDM2 interactions were suppressed (Fig. 5g, 5h and Extended Data Fig. 8c). In addition, acetylation-mimicking mutants of MDM2 and p53, which are replaced with SIRT1-targeted lysine (K) with glutamine (Q), showed reduced binding to MKRN1 (Extended Data Fig. 8 d and 8e) [31, 42]. Moreover, cisplatin-induced degradation of MDM2 was abolished by the inactivation or knockdown of SIRT1 (Fig. 5i, j). Meanwhile, analysis of the intracellular localization revealed that nuclear-localized MKRN1 was increased in the presence of cisplatin, suggesting that MKRN1 translocates to the nucleus upon DNA damage, and degrades MDM2 in the nucleus (Extended Data Fig. 8f). Of note, this observation supports previous findings that MDM2 deacetylated by SIRT1 in the cytoplasm translocates to the nucleus upon DNA damage, where MDM2 is degraded [31]. Collectively, SIRT1 appears to be critical for the MKRN1-dependent modulation of the p53-MDM2 feedback loop.

### MKRN1 is a potential therapeutic target for cancer treatment

We provide evidence that MKRN1 is required for the elimination of DNA-damaged cells by inducing p53-dependent apoptosis. Finally, we tested whether MKRN1 serves as a tumor suppressor in a xenograft tumor model to clarify the in vivo role of MKRN1 as a p53 activator (Fig. 6a) [43]. First, we confirmed that the growth rates of xenograft tumors among WT, MKRN1 KO, or MKRN1 reconstituted cells in MKRN1 KO HT1080 cells did not change (Fig. 6b). However, given that MKRN1 suppresses p53-dependent cell proliferation through the p21 activation as previously demonstrated, MKRN1 KO HT1080 cells might proliferate faster than WT cells [20]. To address this discrepancy, we evaluated mRNA expression levels of p21. Knockdown of MKRN1 increased p21 expression, but neither the mRNA nor protein levels of p21 were altered in MKRN1 KO HT1080 cells (Extended Data Fig. 9a-9c). On the other hand, the expression levels of MDM2, a downstream gene of p53, were increased in MKRN1 knockdown cells together with the p53 stabilization (Extended Data Fig. 1c). However, this observation was not seen in MKRN1 KO HT1080 cells, even though p53 was stabilized after cisplatin treatment (Extended Data Fig. 1a). These observations that there are differences between MKRN1 KO and knockdown cells suggest the possibility that MKRN1 KO HT1080 cells acquired compensatory mechanisms for p53-mediated transcriptional induction at steady state, probably through the process of its cloning. Indeed, similar to MKRN1 KO HT1080 cells, no difference in tumor growth was observed between p53 KO and wild-type HT1080 cells, supporting the idea that the p53 functions at steady-state were compensated in MKRN1 KO HT1080 cells (Extended Data Fig. 9d-9g). Unlike the steady-state condition, we observed differences in xenograft tumor growth in the presence of cisplatin (Fig. 6c). In particular, cisplatin suppressed the growth of xenograft tumors in WT HT1080 cells, whereas it failed to do so in that of MKRN1 KO HT1080 cells (Fig. 6c). The effect of cisplatin was reflected in gross appearance (Fig. 6d), tumor weight (Fig. 6e), and tumor volume (Fig. 6f). Reconstitution of MKRN1 WT, but not the H307Y mutant, in MKRN1 KO HT1080 cells successfully recovered the effects of cisplatin (Fig. 6c–f). These observations suggest that MKRN1 determines the sensitivity to cisplatin, and the loss of MKRN1 is responsible for cisplatin resistance. Immunoblot analysis of the xenograft tumors revealed that both p53 activation and MDM2 degradation in cisplatin-treated mice were attenuated in the xenograft tumors of MKRN1 KO HT1080 cells, confirming that MKRN1-dependent modulation of the p53-MDM2 feedback loop occurred in xenograft tumors (Fig. 6g, i). Collectively, these results suggest that MKRN1 prevents xenograft tumor growth by promoting p53-dependent apoptosis (Fig. 7). Thus, MKRN1 is a potential therapeutic target for cancer treatment.

## Discussion

Since the functions of p53, such as cell cycle arrest or induction of apoptosis, are unnecessary in healthy cells, its expression needs to be maintained at low levels [1, 44, 45]. Therefore, MDM2, which continuously degrades p53 under steady-state conditions, is particularly important in this context. In contrast, the feedback loop that maintains low p53 expression needs to be disabled after DNA damage is detected, and the rapid degradation system removing MDM2 makes quick and reliable activation of p53. However, the mechanisms underlying MDM2 degradation are not fully understood. In this study, we found that the p53 activation induced by DNA damage was diminished due to the failure of MDM2 degradation in MKRN1 KO cells, and identified MKRN1 as the responsible E3 ligase for MDM2 that induces its proteasomal degradation (Fig. 7). Our results indicate that MKRN1 plays a pivotal role in the MDM2 degradation, although previous studies have identified several E3 ligases that target MDM2. For instance, β-TRCP, PCAF, and the anaphase-promoting complex/cyclosome (APC/C) have been identified as E3 ligases for MDM2 [16, 46, 47]. In β-TRCP knockdown cells, only the late phase of the p53 activation was attenuated, suggesting that β-TRCP may be only partially involved in the MDM2 degradation [16]. Consistent with this observation, β-TRCP minimally impacted p53-induced apoptosis [16, 46]. In addition, the contribution of PCAF to p53-induced apoptosis appeared to be minimal [15]. APC/C acts as an E3 ligase for MDM2 only during the G1 and mitotic phases of the cell cycle [47].

A previous study has demonstrated that MKRN1 targets p53 as a substrate under steady-state conditions, and thereby negatively regulates the function of p53 [20]. Taking account of this evidence, MKRN1 targets both p53 and MDM2 depending on the presence or absence of DNA damage stress. Hence, MKRN1 appears to act as a key molecular determinant controlling the balance of the p53-MDM2 feedback loop. Moreover, our results that genetic suppression or pharmacological inhibition of SIRT1 inhibits the binding of MKRN1-MDM2 or MKRN1-p53 suggest that the stress-dependent substrate switching mechanism of MKRN1 is determined by the acetylation state of the substrates. Lysine residues targeted for acetylation are positively charged at intracellular pH and interact with other macromolecules, including other proteins, DNA, and RNA, through intermolecular interactions such as electrostatic forces and hydrogen bonds [28, 29]. That is, acetylation neutralizes the inherent positive charge of lysine residues and potentially inhibits their interactions [28, 29]. This suggests that the acetylation state of the substrates influences electrostatic interactions with amino acid residues crucial for MKRN1 substrate recognition. Thus, the substrate of MKRN1 is determined by SIRT1, indicating that MKRN1 and SIRT1 fine-tune the p53-MDM2 feedback loop through the crosstalk between ubiquitination and acetylation.

On the other hand, this raises the question of how SIRT1 selectively deacetylates its substrates, MDM2 and p53, upon DNA damage. Previous studies have shown that SIRT1 deacetylates MDM2 in response to DNA damage, whereas the p53 deacetylation is suppressed [31, 48]. Although the molecular mechanisms underlying this selectivity remains unclear, one possibility is that the selectivity may be regulated by PTMs of SIRT1 itself or its cofactors. For example, the mechanism by which Homeodomain-interacting protein kinase 2 (HIPK2) regulates SIRT1 function is noteworthy. HIPK2 phosphorylates SIRT1 at Ser682 in response to DNA damage, and then suppresses the p53 deacetylation by blocking its interaction with active regulator of SIRT1 (AROS) required for the p53 deacetylation, without altering the deacetylase activity or localization of SIRT1 itself [49, 50]. Moreover, it has been observed that Thr454 phosphorylation of Deleted in Breast Cancer-1 (DBC1) by Ataxia Telangiectasia Mutated (ATM) and Ataxia Telangiectasia and Rad3-related (ATR) kinases enhance DBC1-SIRT1 binding, thereby reduce DNA damage-dependent SIRT1-p53 interaction, while SIRT1-MDM2 binding is maintained even upon DNA damage [31, 51]. Taken together, these findings suggest that upon DNA damage, substrate selectivity may be regulated by PTMs that alter binding ability of SIRT1 to specific substrates, rather than the enzymatic activity of SIRT1, which makes possible substrate selectivity in which the p53 deacetylation is suppressed while the MDM2 deacetylation is maintained.

We also demonstrated the pro-apoptotic function of MKRN1 in the DDRs at the cellular level, and the anti-tumor function of MKRN1 in vivo. In particular, our in vivo experiments showed that the expression levels of MKRN1 determine the sensitivity to cisplatin, suggesting that reduced MKRN1 expression may cause the cisplatin resistance, although the regulatory mechanism of MKRN1 expression is largely unknown. Therefore, to elucidate the regulatory mechanism of MKRN1 expression may be useful to overcome the cisplatin resistance. In any case, our findings demonstrate that the presence or absence of the MKRN1 expression significantly impacts the efficacy of anticancer drugs such as cisplatin, indicating that MKRN1 activators could complement anticancer drug therapies.

## Material and methods

### Cell lines

Human fibrosarcoma cell line HT1080, human adeno carcinoma cell line A549 and human embryonic kidney 293 A (HEK293A) were obtained from JCRB Cell Bank (Japanese Collection of Research Bioresources Cell Bank). Human-osteosarcoma tumor cell line U2OS were obtained from ATCC (American Type Culture Collection). HT1080 cells were cultured in Dulbecco’s Modified Eagle Medium (DMEM) containing 10% heat-inactivated fetal bovine serum (FBS) and 1% penicillin-streptomycin solution at 37 °C under a 5% CO_2_ atmosphere. A549, HEK293A and U2OS cells were cultured in DMEM containing 5% heat-inactivated FBS and 1% penicillin-streptomycin solution at 37 °C under a 5% CO_2_ atmosphere.

### Reagents and plasmids

All reagents were obtained from commercial sources; dimethyl sulfoxide (DMSO), cisplatin (Wako, Osaka, Japan), Z-VAD-fmk (Peptide Institute, Osaka, Japan), etoposide (VP-16), MG132, nicotinamide, sirtinol (Enzo Life Sciences, Farmingdale, NY, USA), nutlin-3 (Santa Cruz, Dallas, TX, USA), doxorubicin and cycloheximide (Sigma-Aldrich, St. Louis, MO, USA). cDNAs encoding human MKRN1, p53 and MDM2 was obtained by PCR using cDNA from HT1080 cells as a template, and were inserted into pcDNA3.2 with Flag, Myc or 6 × Myc-tagged plasmid. Plasmid transfection was performed using Polyethylenimine “Max” (PEI-MAX) (Polysciences, Warrington, PA, USA), according to the manufacturer’s instructions. siRNAs targeting human MKRN1 (#1: 5’- GGCGAAGCUGAGUCAAGAA -3’, #2: 5’- GGAUCCUCUCCAACUGCAA -3’), human MDM2 (#1: 5’- GACAAAGAAGAGAGUGUGG -3’, #2: 5’- AACCUGAAAUUUAUUCACAUA -3’) and human SIRT1 (#1: 5’- GAUGAAGUUGACCUCCUCA -3’, #2: 5’- UGAAGUGCCUCAGAUAUUA -3’) were purchased from GeneDesign. siGENOME Non-Targeting siRNA Pools (Dharmacon, Lafayette, CO, USA) was used as a negative control. siRNA transfection was performed using Lipofectamine RNAiMAX reagent (Invitrogen, Waltham, MA, USA) according to the manufacturer’s instructions.

### Antibodies

Antibodies against the following proteins were used in this study; FLAG (Sigma-Aldrich, F7425), p53 (Cell Signaling Technology, Danvers, MA, USA, #2524 for immunoblotting and #9282 for immunoprecipitation), MDM2 (#86934), K48-linkage specific polyubiquitin (#8081), p21 (#2946), MKRN1 (Bethyl Laboratories, Montgomery, TX, USA, #A300-990A), Myc (MBL, Tokyo, Japan, 562), GST (Wako, 013-21851), FLAG (014-22383), c-Myc (Santa Cruz, sc-40), MKRN1 (sc-515815), MDM2 (HDM2-323) (sc-56154), caspase-3 (sc-271028), SIRT1 (sc-74465), Ac-Lys (sc-32268), Lamin A/C (sc-376248), α-tubulin (sc-5286), β-actin (sc-47778). Mouse IgG and rabbit IgG (Cell Signaling Technology) were also used in this study.

### Generation of knockout cell lines

All knockout cells were established using the CRISPR/Cas9 system [52, 53]. Guide RNAs (gRNAs) were designed to target a region in exon 1 of the human MKRN1 gene (5′- CAGTCACCGCCCCGTCCCT -3′) or exon 3 of the human p53 gene (5′- ATCTGAGCAGCGCTCATGGTGGG -3′) using CRISPRdirect. gRNA-encoding oligonucleotide was cloned into lentiCRISPRv2 plasmid (Addgene, Watertown, MA, USA), and the plasmid was transfected with HEK293A cells together with a packaging plasmid psPAX2 and an envelope plasmid pVSV-G. The supernatants were collected and used to infect HT1080 cells, and then infected cells were selected with puromycin and cloned by limiting dilution to obtain 100% efficiency. To determine the mutations of each gene in cloned cells, the genomic sequence around the target region was analyzed by PCR-direct sequencing using extracted DNA from each clone as a template and the following primers: 5′- GGAACAACAGCCACAACATC -3’ and 5′- TGCAGAGATCGAGACAACG -3′ for MKRN1. 5′- AGAGACCCCAGTTGCAAACC -3′ and 5′- CCCTGCCCTCAACAAGATGT -3′ for p53.

### Generation of reconstituted cells

A packaging cell line Phoenix-AMPHO was transfected with pMXs-IH inserted with cDNAs encoding human MKRN1 WT, human MKRN1 H307Y mutant and human MKRN1 ΔRING mutant. After 48 h, lentivirus-containing supernatants were harvested and centrifuged at 1000 rpm for 5 min to discard the debris. HT1080 cells were incubated with the virus-containing medium with 10 μg/mL polybrene for 48 h and then un-infected cells were eliminated by hygromycin selection.

### Colorimetric cell viability assay

Cells were seeded on 96-well plates at a density of 1 × 10^4^ / well. After indicated stimulation or treatment, cell viability was determined using CellTiter 96 Cell Proliferation Assay (Promega, Madison, WI, USA) or CellTiter-Glo 2.0 Cell Viability Assay (Promega) according to the manufacturer’s protocol. The absorbance was read at 490 nm or the luminescence was measured, using a microplate reader. Data are normalized to control (100%) without stimulus, unless noted otherwise.

### Quantitative real-time PCR

Isolation of total RNA and template cDNA were conducted as previously described [54]. The primers used for quantitative real-time PCR were as follows: 5′- CCCCTCCTGGCCCCTGTCATCTTC -3′ and 5′- GCAGCGCCTCACAACCTCCGTCAT -3′ for p53. 5′- ACCTCACAGATTCCAGCTTCG -3′ and 5′- TTTCATAGTATAAGTGTCTTTTT -3′ for MDM2. 5′- GAGAGGTCTTTTTCCGAGTGG -3′ and 5′- GGAGGAAGTCCAATGTCCAG -3′ for Bax. 5′- TTGTGCTGGTGCCCGTTCCA -3′ and 5′- AGGCTAGTGGTCACGTTTGGCT -3′ for Puma. 5′- TGGAAGTCGAGTGTGCTACTCAA -3′ and 5′- CAGAAGAGTTTGGATATCAGATTCAGA -3′ for Noxa. 5′- GAGGCCGGGATGAGTTGGGAGGAG -3′ and 5′- CAGCCGGCGTTTGGAGTGGTAGAA -3′ for p21. 5′- AACAGCCTCAAGATCATCAGC -3′ and 5′- GGATGATGTTCTGGAGAGCC -3′ for GAPDH.

### FACS analysis

Cells were seeded on 12-well plates at a density of 2 × 10^5^ / well. After indicated stimulation or treatment, cells were detached from the culture dish by trypsin treatment and dispersed by pipetting as mild as possible to avoid mechanical damages to the cells. For annexin V and propidium iodide (PI) staining, cells were labeled with annexin V-FITC (MBL) and PI for 15 min in binding buffer containing 10 mM HEPES (pH 7.5), 150 mM NaCl, 5 mM KCl, 1 mM MgCl_2_, 1.8 mM CaCl_2_. Fluorescence levels of the cells were detected by CytoFLEX (Beckman Coulter, Brea, CA, USA), and apoptotic cells were analyzed by using CytoExpert (Beckman Coulter).

### Immunoblot analysis

Cells were seeded on 12-well plates at a density of 2 × 10^5^ / well. After indicated stimulation or treatment, cells were lysed in DISC lysis buffer containing 20 mM Tris-HCl (pH 7.4), 150 mM NaCl, 1% Triton X-100, 10% glycerol and 1% protease inhibitor cocktails (Nacalai Tesque, Kyoto, Japan). The lysates were centrifuged at 4 °C at 15,000 rpm for 15 min. After centrifugation, the cell extracts were resolved by SDS-PAGE and analyzed as previously described [55].

### Nuclear extraction

Cells were seeded on 6-well plates at a density of 5 × 10^5^ / well. After indicated stimulation or treatment, cells were lysed in ice-cold lysis buffer containing 10 mM HEPES (pH 7.5), 10 mM KCl, 0.1 mM EGTA, 0.1 mM EDTA, 1 mM DTT, and 1% protease inhibitor cocktails. The lysates were added 1% NP-40 and then centrifuged at 4 °C at 2500 rpm for 3 min. After the supernatants containing cytoplasmic fraction were removed, the pellets were suspended in ice-cold lysis buffer containing 20 mM HEPES (pH 7.5), 400 mM NaCl, 1 mM EGTA, 1 mM DTT and 1% protease inhibitor cocktails for 15 min with vortexed every 5 min. The lysates were centrifuged at 4 °C at 15,000 rpm for 15 min and then the supernatants were collected as nuclear fraction.

### Immunoprecipitation

Cells were seeded on 6-well plates at a density of 5 × 10^5^ / well. After indicated stimulation or treatment, cells were lysed in ice-cold lysis buffer containing 23 mM Tris-HCl (pH 7.4), 150 mM NaCl, 4 mM EDTA-2Na (pH 8.0), 0.4% Triton X-100, 1% sodium deoxycholate, 0.6% NP-40, 0.06% SDS and 1% protease inhibitor cocktails. The lysates were centrifuged at 4 °C at 15,000 rpm for 15 min. After centrifugation, the cell extracts were immunoprecipitated with 20 μL anti-FLAG agarose beads (Sigma-Aldrich), anti-Myc agarose beads (10D11, Wako) or protein G-Sepharose beads (Amersham Biosciences, Uppsala, Sweden) with the indicated antibodies for 2 h at 4 °C. The beads were washed four times with the same buffer before analysis by SDS-PAGE or affinity purification.

### In vivo ubiquitination assay

HEK293A cells transfected with the indicated plasmids were treated with MG132 for 4-6 h before collection. The cells were lysed in ice-cold lysis buffer supplemented with 10 mM N-ethylmaleimide (NEM) and then the cell lysates centrifuged at 4 °C at 15,000 rpm for 15 min. 10% SDS was added to the lysates and heated at 98 °C to disrupt noncovalent protein-protein interactions. The lysates subjected to immunoprecipitation with anti-FLAG agarose beads or anti-Myc agarose beads for 4 h at 4 °C and then the beads were washed four times with the same buffer before immunoblot analysis.

### In vitro ubiquitination assay

In vitro ubiquitination assay was performed using the E2-Ubiquitin Conjugation Kit (Abcam, Cambridge, UK), according to the manufacturer’s protocol with minor modifications. Briefly, ubiquitination reactions were carried out with purified FLAG-MKRN1 as an E3 enzyme, with recombinant GST-MDM2 or purified 6Myc-MDM2 by immunoprecipitation as a substrate, in a reaction buffer containing recombinant biotinylated ubiquitin, E1-activating enzyme, His-tagged E2-conjugating enzyme (Ubc5a) and Mg-adenosine triphosphate. The reaction mixtures were incubated for 4 h at 37 °C and then the reactions were terminated by the addition of 2 × Laemmli sample buffer (non-reducing). The reaction mixtures were subjected to immunoblot analysis.

### Recombinant protein purification

Purification of FLAG-MKRN1 was carried out as previously described [56]. For purification of GST-MDM2, the E. coli BL21 (DE3) (NEB) strain was introduced with pGEX6P-1 inserted with cDNAs encoding hMDM2 and treated with 1 mM isopropyl β-1-thiogalactopyranoside (IPTG) for 6 h at 37 °C. The recombinant proteins were extracted with ice-cold lysis buffer containing 20 mM Tris-HCl (pH 7.4), 0.5% Triton X-100, 150 mM NaCl and 2 mM EDTA and then were affinity purified using glutathione Sepharose 4B beads (GE healthcare, Chicago, IL, USA).

### In vitro binding assay

Purified FLAG-MKRN1 dissolved in IP lysis buffer containing 20 mM Tris-HCl (pH 7.4), 150 mM NaCl, 1% Triton X-100, 1% sodium deoxycholate, 1% protease inhibitor cocktails was mixed with GST-MDM2 conjugated with glutathione Sepharose 4B beads. The mixtures were subjected to binding reaction for 4 h at 4 °C and then the beads were washed four times with the same buffer before immunoblot analysis.

### Alkaline comet assay

The Comet assay using Comet SCGE assay kit (Enzo Life Sciences, Inc., New York, USA) was performed as previously described [57]. Comet scores were calculated according to comet tail length (*n* = 50).

### In vivo tumor xenograft model

BALB/c-nu/nu male nude mice (5-6 weeks old) were obtained from CLEA Japan (Tokyo, Japan). HT1080 cells were subcutaneously injected into nude mice (3 × 10^6^ cells in 0.2 mL PBS/mouse). A week after subcutaneous injection, mice were randomly divided into two groups and treated normal saline (NS) or cisplatin (2 mg/kg) by intraperitoneal injection every other day. Tumor size was measured twice a week using a caliper and calculated using the following formula: tumor volume = length × width^2^ / 2. Two weeks later, animals were sacrificed, and the xenograft tumors were harvested. Mice were maintained according to the Guidelines for Animal Experimentation of Tohoku University. No sample size estimation or blinding was conducted.

### Statistical analysis

All experiments were repeated at least three independent times. The value was described as the mean ± standard deviation (S.D.) using Prism 9 Version 9.5.1 software (GraphPad, La Jolla, CA, USA). Two groups were compared using Student’s t-test. Multiple-group comparisons were conducted using the one-way ANOVA analysis of variance followed by the Tukey-Kramer test using Prism software (GraphPad). Data were considered significant when *** *p* <  0.001, ** *p*  <  0.01, * *p* <  0.05.

## Supplementary information

Extended data figures

Full and uncropped WB (main figures)

Full and uncropped WB (Extended data figures)

## Data Availability

The data presented in this study are available in article.
