# Supplementary material for: Identification of MKRN1 as a key modulator of the p53-MDM2 feedback loop
Source: Cell Death Differ. 2026 Jan 30;33(7):1474–87. doi: 10.1038/s41418-026-01662-4 (PMC13342678; doi:10.1038/s41418-026-01662-4)
Supplement: Supplementary file 1 — Extended data figures [file 41418_2026_1662_MOESM1_ESM.pdf]

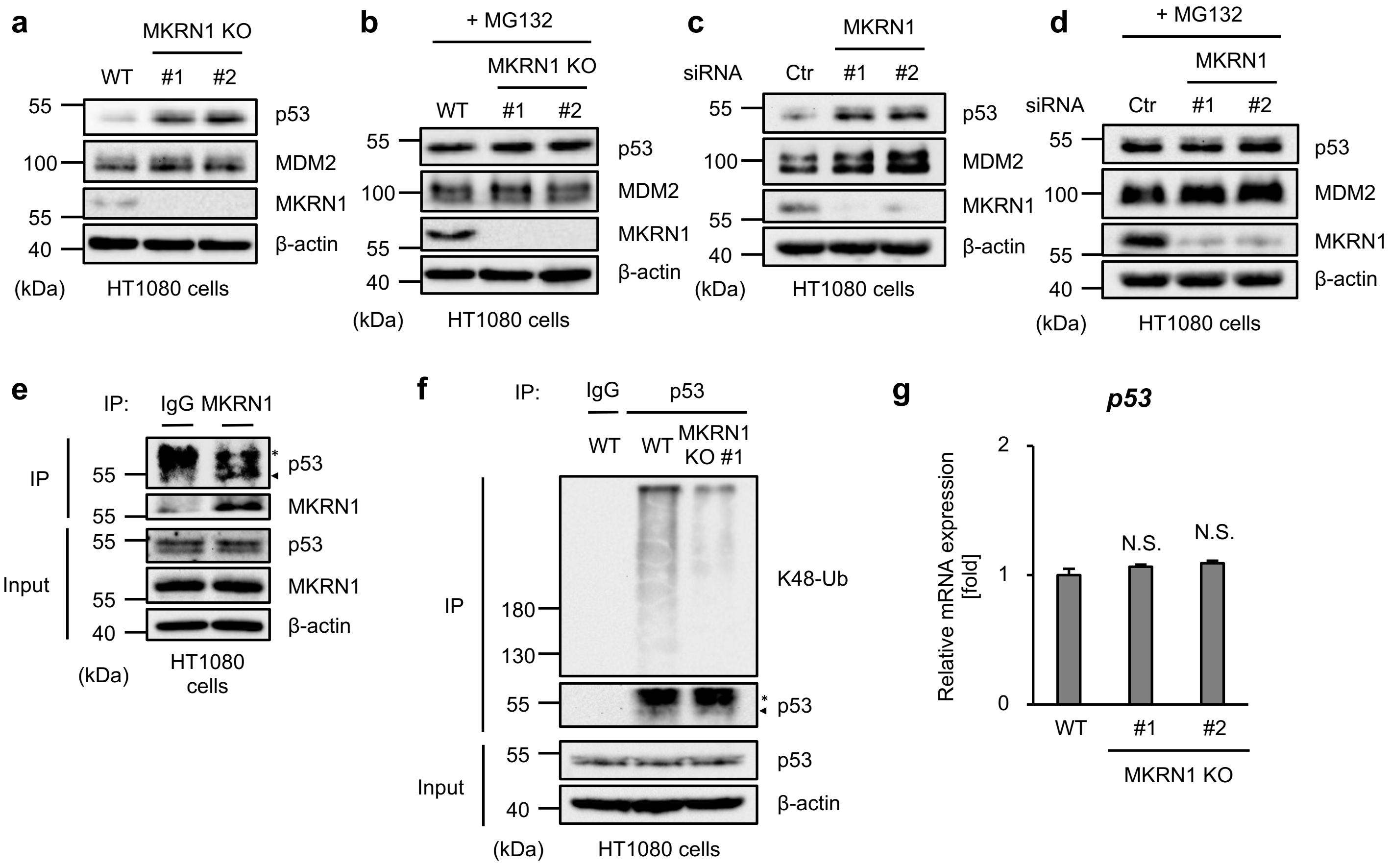

Extended Data Figure 1.

(**a**) Cell lysates from HT1080 cells were subjected to immunoblotting with the indicated antibodies. (**b**) HT1080 cells were treated with MG132 (5 μM) for 4 h. Cell lysates were subjected to immunoblotting with the indicated antibodies. (**c**) HT1080 cells were transfected with siRNA for negative control or MKRN1 (MKRN1 #1 or MKRN1 #2). After 48 h, cell lysates were subjected to immunoblotting with the indicated antibodies. (**d**) HT1080 cells were transfected with siRNA for negative control or MKRN1 (MKRN1 #1 or MKRN1 #2). After 48 h, cells were treated with MG132 (5 μM) for 4 h. Cell lysates were subjected to immunoblotting with the indicated antibodies. (**e**) HT1080 cells were treated with MG132 (5 μM) for 4 h. Cell lysates were immunoprecipitated with anti-IgG or MKRN1 antibody and then subjected to immunoblotting with the indicated antibodies. The band indicated by an asterisk is a non-specific band. (**f**) HT1080 cells were treated with MG132 (5 μM) for 4 h. Cell lysates were immunoprecipitated with anti-IgG or p53 antibody and then subjected to immunoblotting with the indicated antibodies. The band indicated by an asterisk is a non-specific band. (**g**) mRNA levels of p53 in HT1080 cells were analyzed by quantitative real-time PCR (normalized with GAPDH mRNA levels). Data shown are the mean ± S.D. (n = 3). Significant differences were determined by one-way ANOVA, followed by Tukey-Kramer test; N.S.: not significant.

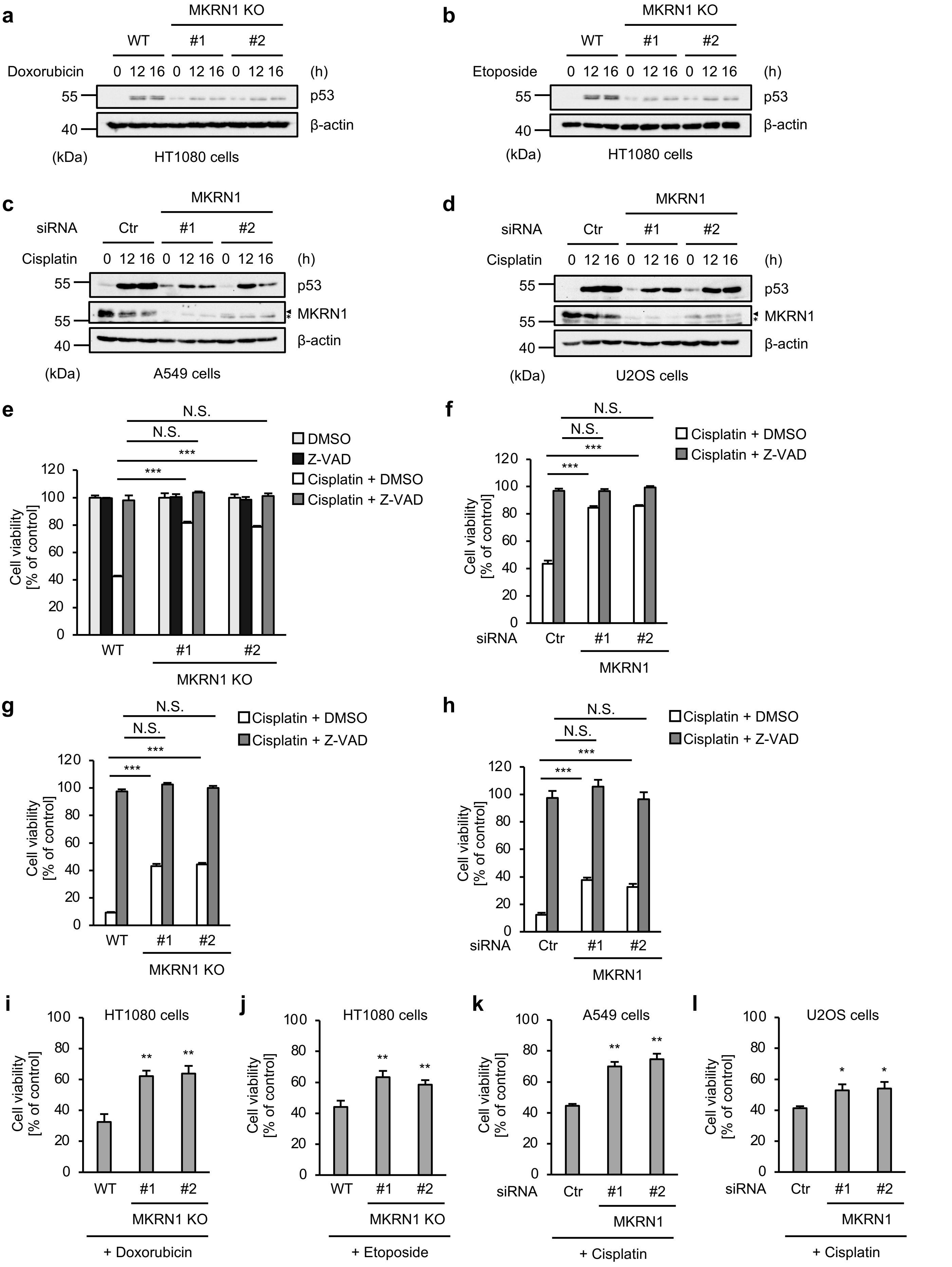
a
MKRN1 KO
WT #1 #2
Doxorubicin 0 12 16 0 12 16 0 12 16 (h)
55 p53
40 β-actin
(kDa) HT1080 cells
b
MKRN1 KO
WT #1 #2
Etoposide 0 12 16 0 12 16 0 12 16 (h)
55 p53
40 β-actin
(kDa) HT1080 cells
c
MKRN1
siRNA Ctr #1 #2
Cisplatin 0 12 16 0 12 16 0 12 16 (h)
55 p53
55 MKRN1
40 β-actin
(kDa) A549 cells
d
MKRN1
siRNA Ctr #1 #2
Cisplatin 0 12 16 0 12 16 0 12 16 (h)
55 p53
55 MKRN1
40 β-actin
(kDa) U2OS cells
e
N.S.
N.S.
***
***
DMSO
Z-VAD
Cisplatin + DMSO
Cisplatin + Z-VAD
Cell viability [% of control]
WT #1 #2
MKRN1 KO
f
N.S.
N.S.
***
***
Cisplatin + DMSO
Cisplatin + Z-VAD
Cell viability [% of control]
siRNA Ctr #1 #2
MKRN1
g
N.S.
N.S.
***
***
Cisplatin + DMSO
Cisplatin + Z-VAD
Cell viability [% of control]
WT #1 #2
MKRN1 KO
h
N.S.
N.S.
***
***
Cisplatin + DMSO
Cisplatin + Z-VAD
Cell viability [% of control]
siRNA Ctr #1 #2
MKRN1
i
HT1080 cells
Cell viability [% of control]
** **
WT #1 #2
MKRN1 KO
+ Doxorubicin
j
HT1080 cells
Cell viability [% of control]
** **
WT #1 #2
MKRN1 KO
+ Etoposide
k
A549 cells
Cell viability [% of control]
** **
siRNA Ctr #1 #2
MKRN1
+ Cisplatin
l
U2OS cells
Cell viability [% of control]
* *
siRNA Ctr #1 #2
MKRN1
+ Cisplatin

Extended Data Figure 2.

(**a**)(**b**) HT1080 cells were treated with doxorubicin (1 μg/mL) (a) or etoposide (20 μM) (b) for the indicated periods. Cell lysates were subjected to immunoblotting with the indicated antibodies. (**c**)(**d**) A549 (c) or U2OS (d) cells were transfected with siRNA for negative control or MKRN1 (MKRN1 #1 or MKRN1 #2). After 48 h, cells were treated with cisplatin (25 μM) for the indicated periods. Cell lysates were subjected to immunoblotting with the indicated antibodies. The band indicated by an asterisk is a non-specific band. (**e**) HT1080 cells were treated with cisplatin (25 μM) for 24 h in the presence of DMSO or Z-VAD-fmk (20 μM) and then subjected to Cell Proliferation Assay. Data shown are the mean ± S.D. (n = 3). Significant differences were determined by one-way ANOVA, followed by Tukey-Kramer test; *** $p < 0.001$, N.S.: not significant. (**f**) HT1080 cells were transfected with siRNA for negative control or MKRN1 (MKRN1 #1 or MKRN1 #2). After 48 h, cells were treated with cisplatin (25 μM) for 24 h and then subjected to Cell Proliferation Assay. Data shown are the mean ± S.D. (n = 3). Significant differences were determined by one-way ANOVA, followed by Tukey-Kramer test; *** $p < 0.001$, N.S.: not significant. (**g**) HT1080 cells were treated with cisplatin (25 μM) for 24 h in the presence of DMSO or Z-VAD-fmk (20 μM) and then subjected to Cell Viability Assay. Data shown are the mean ± S.D. (n = 3). Significant differences were determined by one-way ANOVA, followed by Tukey-Kramer test; *** $p < 0.001$, N.S.: not significant. (**h**) HT1080 cells were transfected with siRNA for negative control or MKRN1 (MKRN1 #1 or MKRN1 #2). After 48 h, cells were treated with cisplatin (25 μM) for 24 h in the presence of DMSO or Z-VAD-fmk (20 μM) and then subjected to Cell Viability Assay. Data shown are the mean ± S.D. (n = 3). Significant differences were determined by one-way ANOVA, followed by Tukey-Kramer test; *** $p < 0.001$, N.S.: not significant. (**i**)(**j**) HT1080 cells were treated with doxorubicin (1 μg/mL) (i) or etoposide (20 μM) (j) for 24 h and then subjected to Cell Proliferation Assay. Data shown are the mean ± S.D. (n = 3). Significant differences were determined by one-way ANOVA, followed by Tukey-Kramer test; ** $p < 0.01$. (**k**)(**l**) A549 (k) or U2OS (l) cells were transfected with siRNA for negative control or MKRN1 (MKRN1 #1 or MKRN1 #2). After 48 h, cells were treated with cisplatin (25 μM) for 24 h and then subjected to Cell Proliferation Assay. Data shown are the mean ± S.D. (n = 3). Significant differences were determined by one-way ANOVA, followed by Tukey-Kramer test; ** $p < 0.01$, * $p < 0.05$.

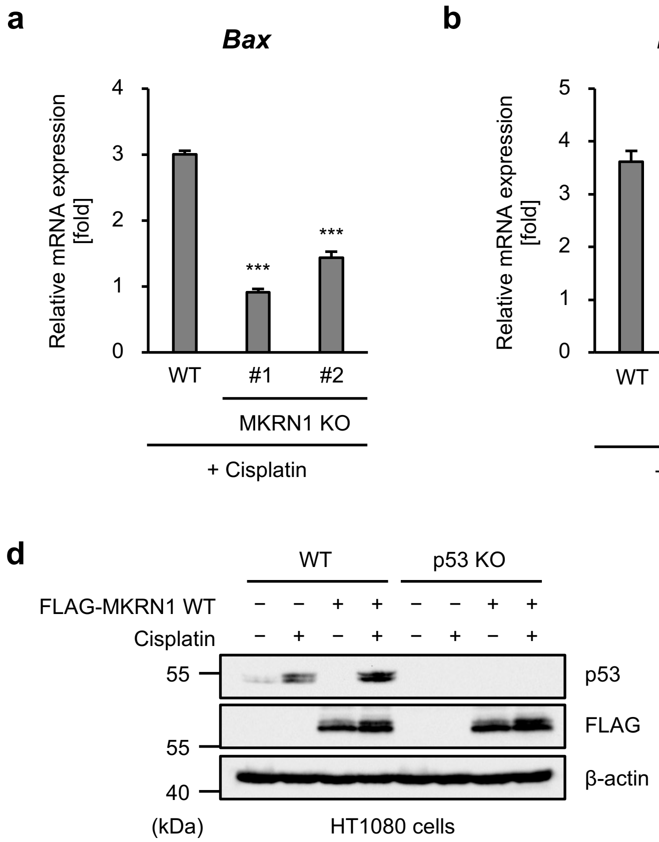

Extended Data Figure 3.

(**a**)-(**c**) HT1080 cells were treated with cisplatin (25 μM) for 16 h. mRNA levels of Bax, Puma and Noxa were analyzed by quantitative real-time PCR (normalized with GAPDH mRNA levels). Data shown are the mean ± S.D. (n = 3). Significant differences were determined by one-way ANOVA, followed by Tukey-Kramer test; *** $p < 0.001$, ** $p < 0.01$. (**d**) HT1080 cells were transfected with the indicated plasmids for 24 h, and then treated with cisplatin (25 μM) for 24 h. Cell lysates were subjected to immunoblotting with the indicated antibodies.

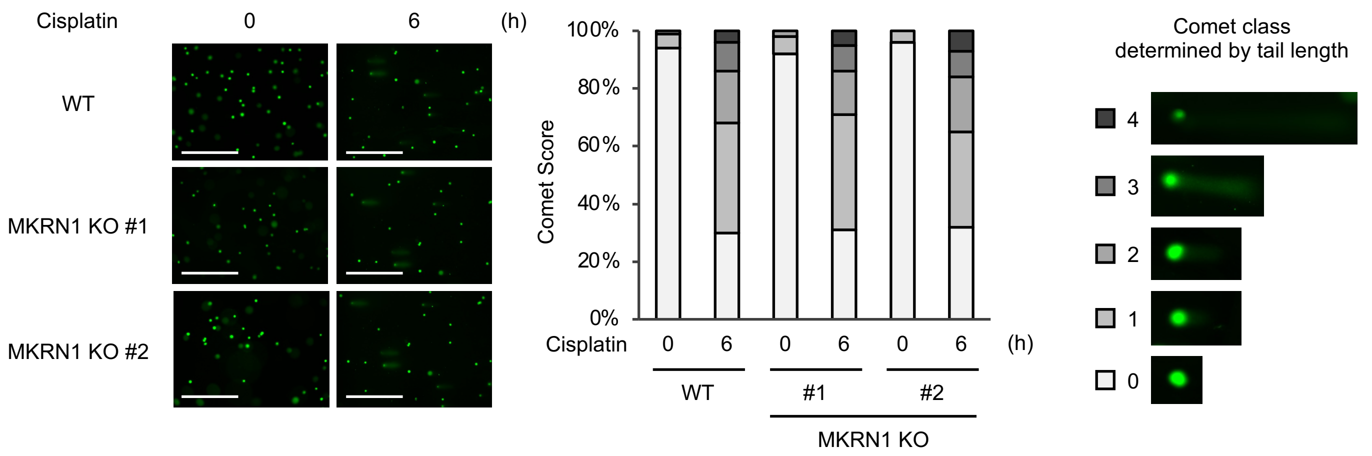

Extended Data Figure 4.

HT1080 cells were treated with cisplatin (25 μM) for the indicated periods and then performed the comet assay as described in the methods section (scale bar, 100 μm). The graph depicts the comet scores that were calculated according to comet tail length (n = 50).

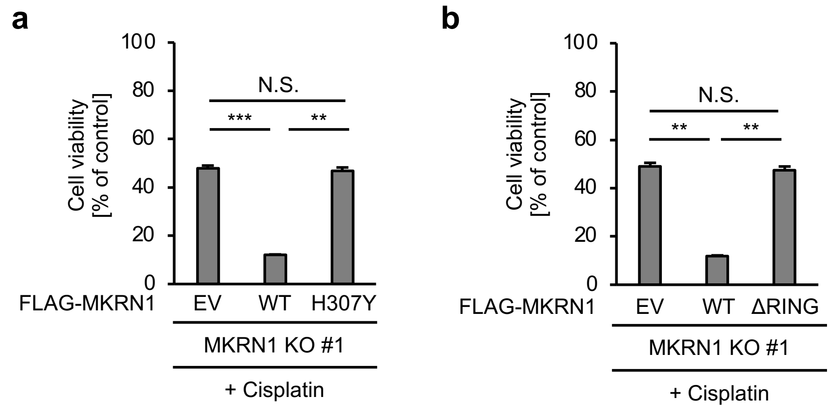

Extended Data Figure 5.

(**a**)(**b**) HT1080 cells were treated with cisplatin (25 μM) for 24 h and then subjected to Cell Viability Assay. Data shown are the mean ± S.D. (n = 3).

Significant differences were determined by one-way ANOVA, followed by Tukey-Kramer test; *** $p < 0.001$, ** $p < 0.01$, N.S.: not significant.

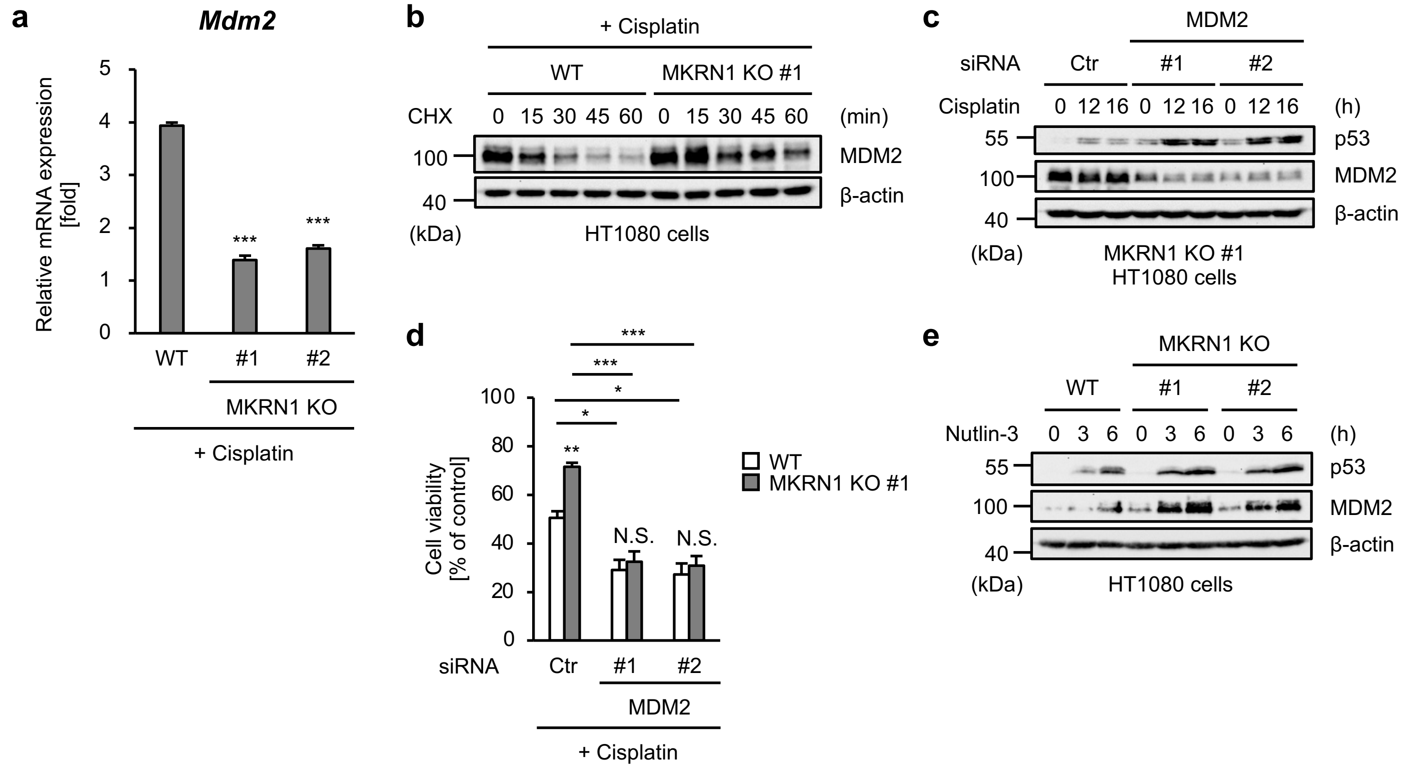

Extended Data Figure 6.

(**a**) HT1080 cells were treated with cisplatin (25 μM) for 16 h. mRNA levels of MDM2 were analyzed by quantitative real-time PCR (normalized with GAPDH mRNA levels). Data shown are the mean ± S.D. (n = 3). Significant differences were determined by one-way ANOVA, followed by Tukey-Kramer test; *** $p < 0.001$. (**b**) HT1080 cells were pretreated with cisplatin (25 μM) for 12 h and then treated with cycloheximide (10 μg/mL) for the indicated periods. Cell lysates were subjected to immunoblotting with the indicated antibodies. (**c**) MKRN1 KO HT1080 cells were transfected with siRNA for negative control or MDM2 (MDM2 #1 or MDM2 #2). After 48 h, cells were treated with cisplatin (25 μM) for the indicated periods. Cell lysates were subjected to immunoblotting with the indicated antibodies. (**d**) HT1080 cells were transfected with siRNA for negative control or MDM2 (MDM2 #1 or MDM2 #2). After 48 h, cells were treated with cisplatin (25 μM) for 24 h and then subjected to Cell Proliferation Assay. Data shown are the mean ± S.D. (n = 3). Significant differences were determined by one-way ANOVA, followed by Tukey-Kramer test; *** $p < 0.001$, ** $p < 0.01$, * $p < 0.05$, N.S.: not significant. (**e**) HT1080 cells were treated with nutlin-3 (10 μM) for the indicated periods. Cell lysates were subjected to immunoblotting with the indicated antibodies.

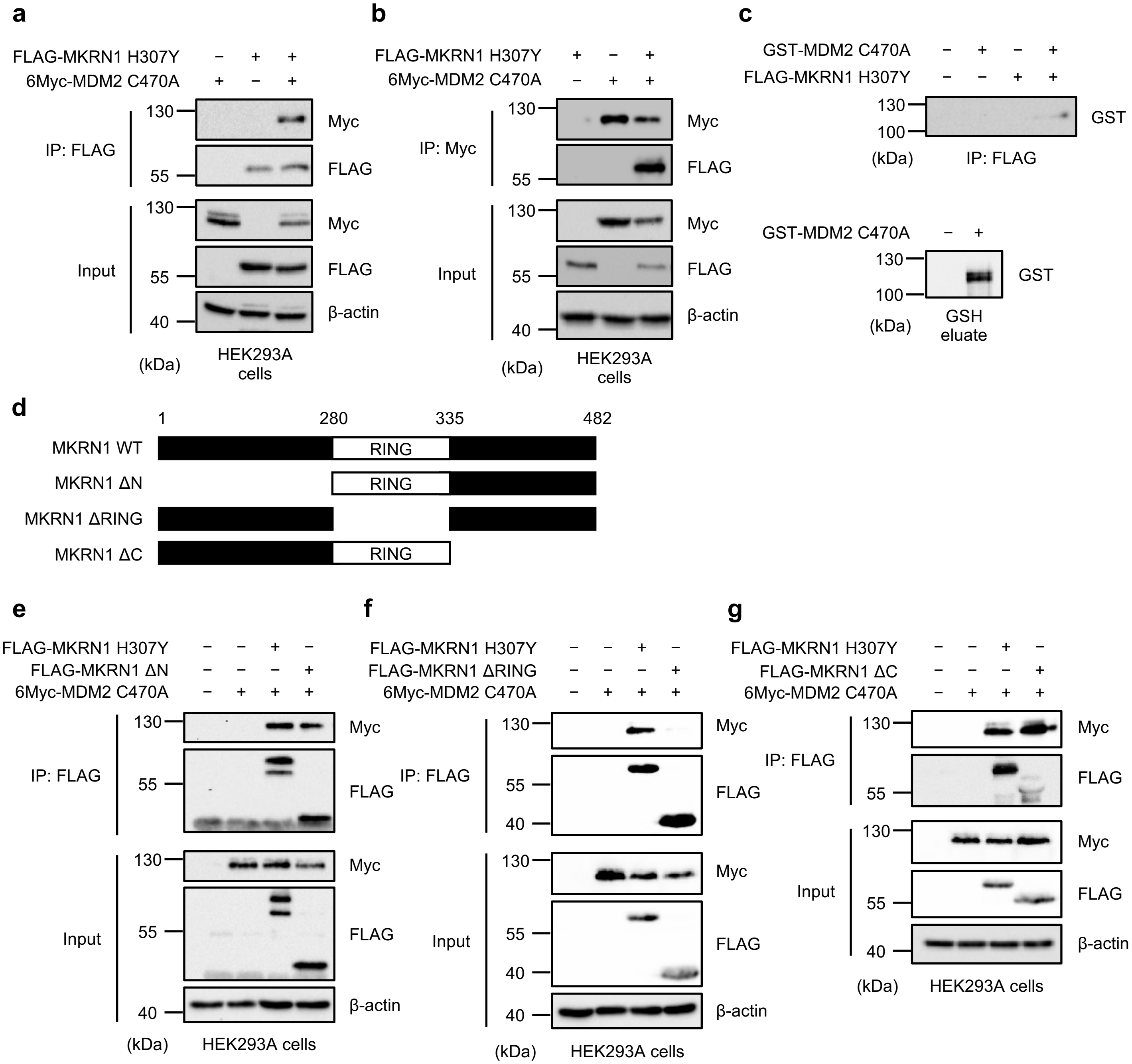

Extended Data Figure 7.

(**a**) HEK293A cells were transfected with the indicated plasmids for 24 h. Cell lysates were immunoprecipitated with anti-FLAG agarose beads and then subjected to immunoblotting with the indicated antibodies. (**b**) HEK293A cells were transfected with the indicated plasmids for 24 h. Cell lysates were immunoprecipitated with anti-Myc agarose beads and then subjected to immunoblotting with the indicated antibodies. (**c**) *In vitro* binding reactions were performed using GST-MDM2 C470A recombinant proteins and affinity-purified FLAG-MKRN1 H307Y and then subjected to immunoblotting with the indicated antibodies. (**d**) The deletion mutants of MKRN1. (**e**)-(**g**) HEK293A cells were transfected with the indicated plasmids for 24 h. Cell lysates were immunoprecipitated with anti-FLAG agarose beads and then subjected to immunoblotting with the indicated antibodies.

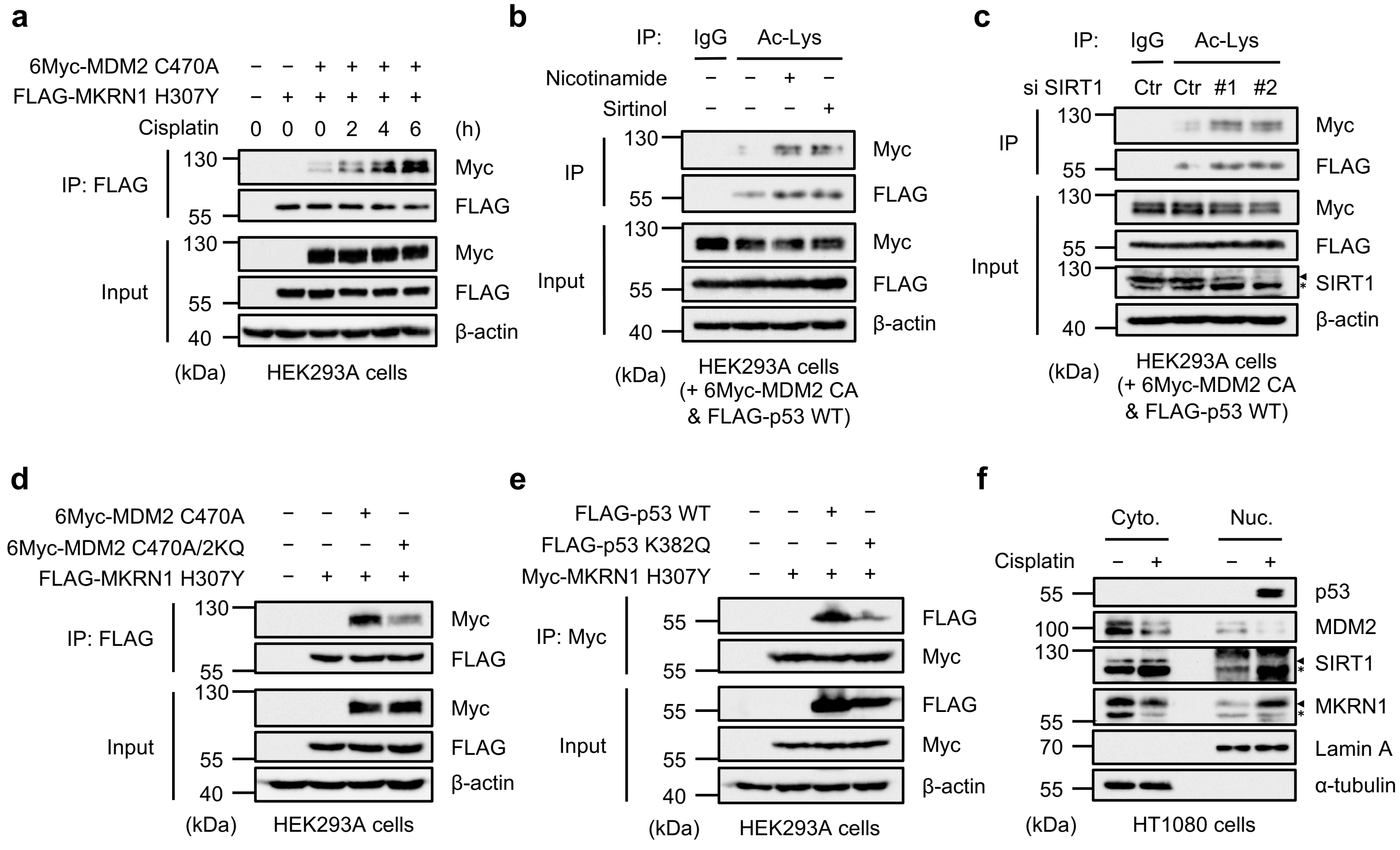

Extended Data Figure 8.

(**a**) HEK293A cells were transfected with the indicated plasmids for 24 h and then treated with cisplatin (25 μM) for the indicated periods. Cell lysates were immunoprecipitated with anti-FLAG agarose beads and then subjected to immunoblotting with the indicated antibodies. (**b**) HEK293A cells were transfected with the indicated plasmids for 24 h and treated with nicotinamide (1 mM) or sirtinol (10 μM) for 6 h. Cell lysates were immunoprecipitated with anti-IgG or Ac-Lys antibody and then subjected to immunoblotting with the indicated antibodies. (**c**) HEK293A cells were transfected with siRNA for negative control or SIRT1 (SIRT1 #1 or SIRT1 #2). After 24 h, cells were transfected with the indicated plasmids for 24 h. Cell lysates were immunoprecipitated with anti-IgG or Ac-Lys antibody and then subjected to immunoblotting with the indicated antibodies. (**d**)(**e**) HEK293A cells were transfected with the indicated plasmids for 24 h. Cell lysates were immunoprecipitated with anti-FLAG agarose beads (d) or anti-Myc agarose beads (e) and then subjected to immunoblotting with the indicated antibodies. MDM2 2KQ: K182Q/K185Q. (**f**) HT1080 cells were treated with cisplatin (25 μM) for 16 h. The nuclear and cytosol extracts were subjected to immunoblotting with the indicated antibodies. The band indicated by an asterisk is a non-specific band.

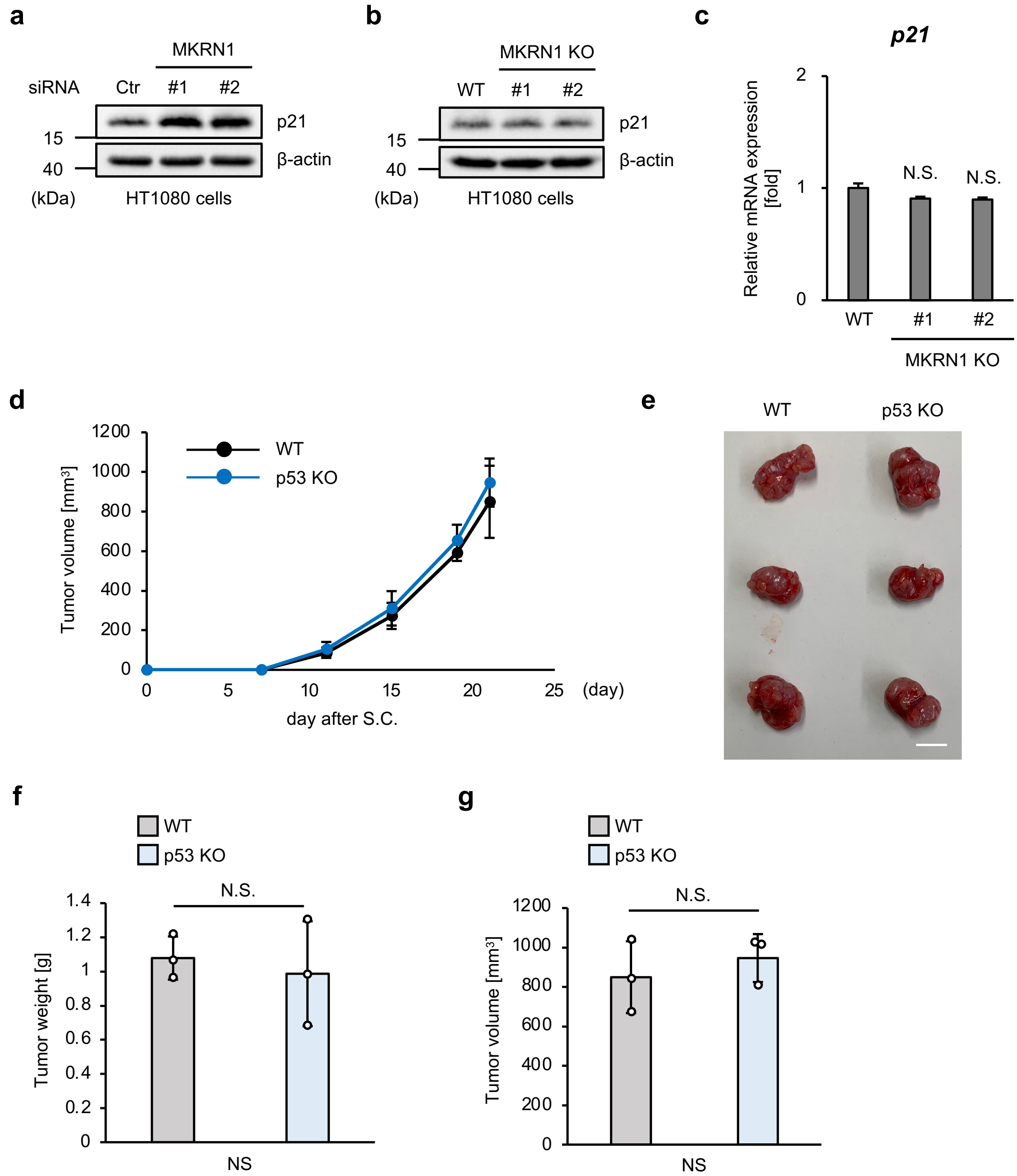

Extended Data Figure 9.

(**a**) HT1080 cells were transfected with siRNA for negative control or MKRN1 (MKRN1 #1 or MKRN1 #2). After 48 h, cell lysates were subjected to immunoblotting with the indicated antibodies. (**b**) Cell lysates from HT1080 cells were subjected to immunoblotting with the indicated antibodies. (**c**) mRNA levels of p21 in HT1080 cells were analyzed by quantitative real-time PCR (normalized with GAPDH mRNA levels). Data shown are the mean ± S.D. (n = 3). Significant differences were determined by one-way ANOVA, followed by Tukey-Kramer test; N.S.: not significant. (**d**) Tumor volume was measured and is shown as the mean ± S.D. (WT: n = 3, p53 KO: n = 3). (**e**) A representative image of tumor xenografts harvested at day 27 (scale bar, 10 mm). (**f**)(**g**) Tumor weight (f) and tumor volume (g) were measured at day 27. Data shown are the mean ± S.D. (WT: n = 3, p53 KO: n = 3). Significant differences were determined by Student's t-test; N.S.: not significant.
