# Supplementary material for: Identification of MKRN1 as a key modulator of the p53-MDM2 feedback loop
Source: Cell Death Differ. 2026 Jan 30;33(7):1474–87. doi: 10.1038/s41418-026-01662-4 (PMC13342678; doi:10.1038/s41418-026-01662-4)
Supplement: Supplementary file 2 — Full and uncropped WB (main figures) [file 41418_2026_1662_MOESM2_ESM.pdf]

Full-length and uncropped western blot for Figure 1

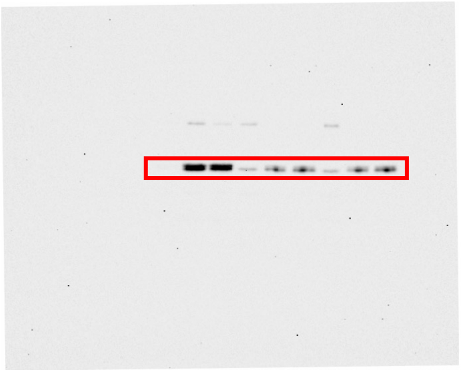

Figure 1a p53

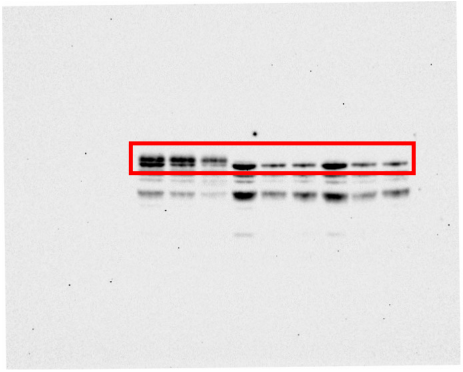

Figure 1a MKRN1

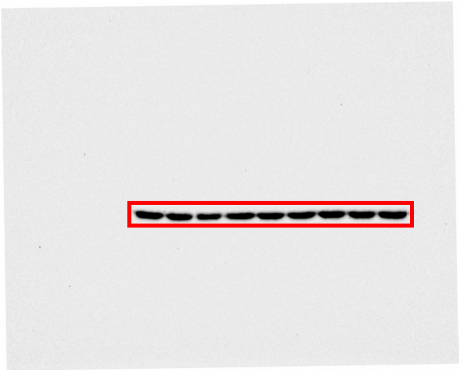

Figure 1a β-actin

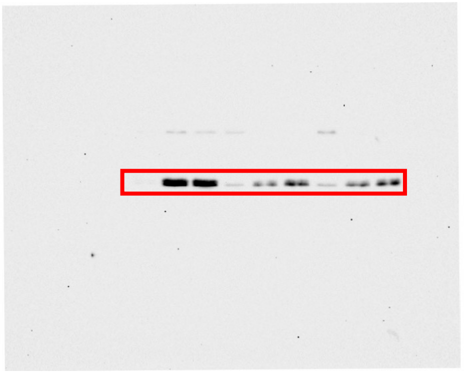

Figure 1b p53

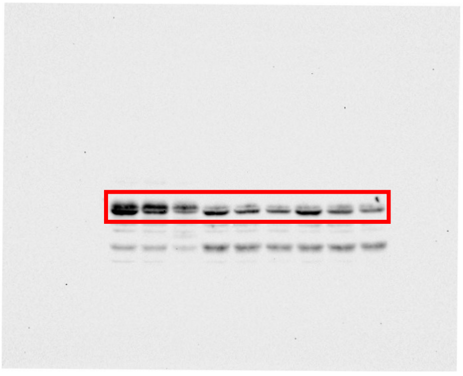

Figure 1b MKRN1

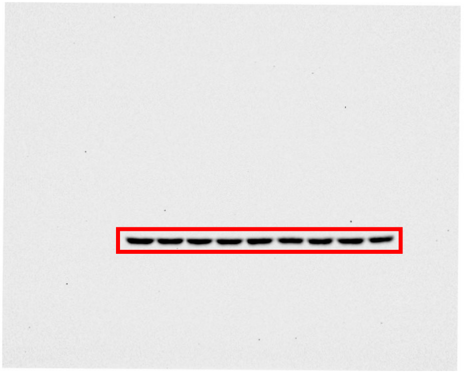

Figure 1b β-actin

Full-length and uncropped western blot for Figure 1

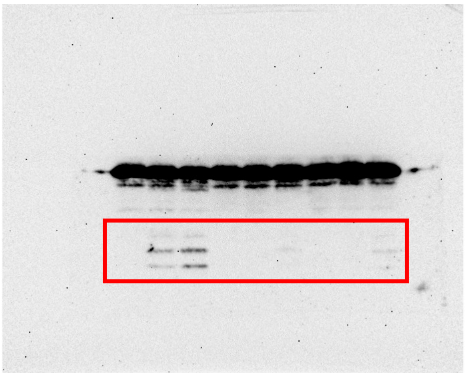

Figure 1e cleaved-caspase-3

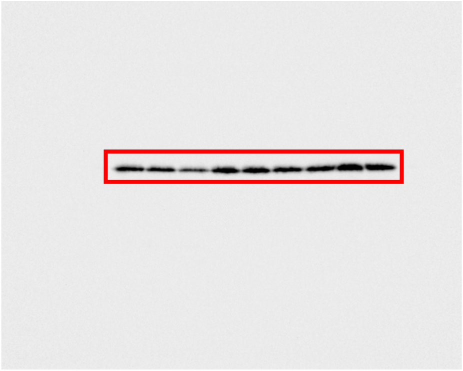

Figure 1e pro-caspase-3

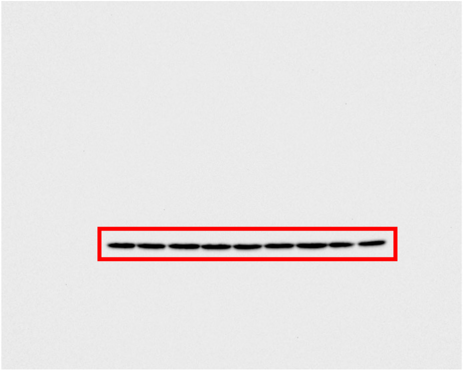

Figure 1e β-actin

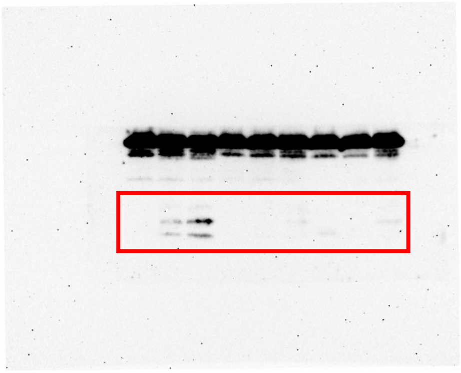

Figure 1f cleaved-caspase-3

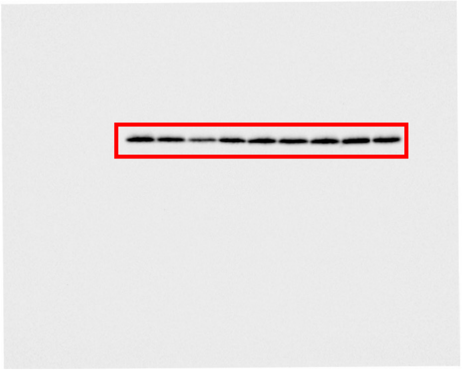

Figure 1f pro-caspase-3

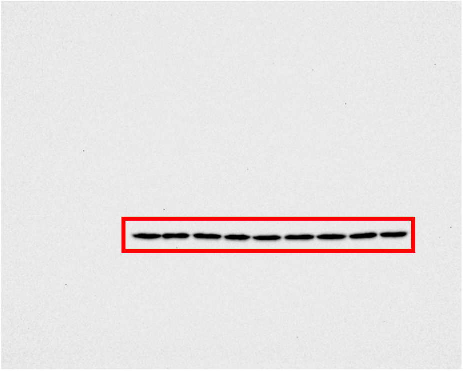

Figure 1f β-actin

Full-length and uncropped western blot for Figure 2

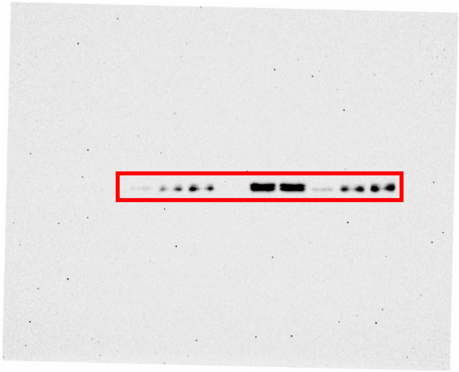

Figure 2a p53

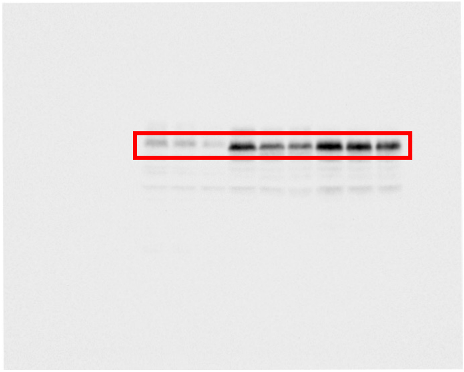

Figure 2a MKRN1

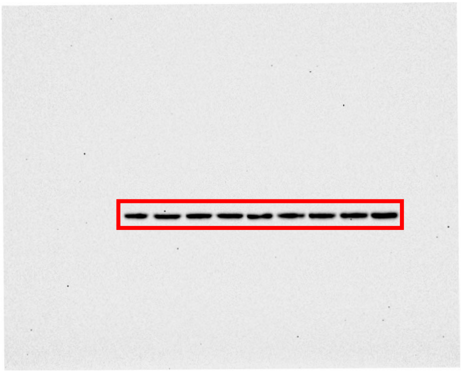

Figure 2a β-actin

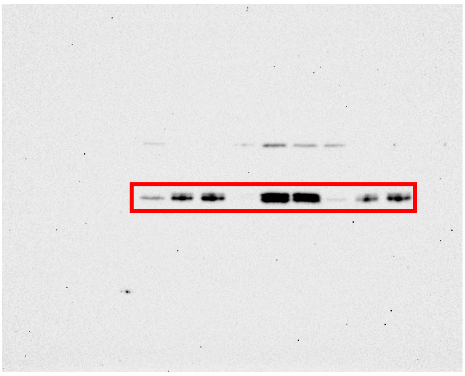

Figure 2b p53

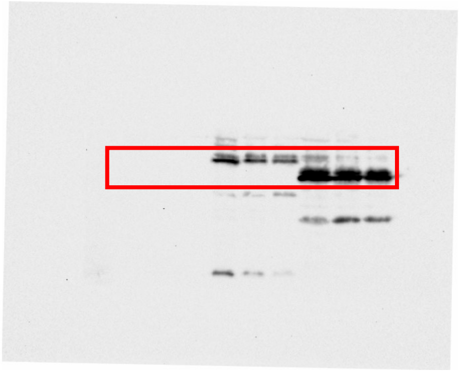

Figure 2b MKRN1

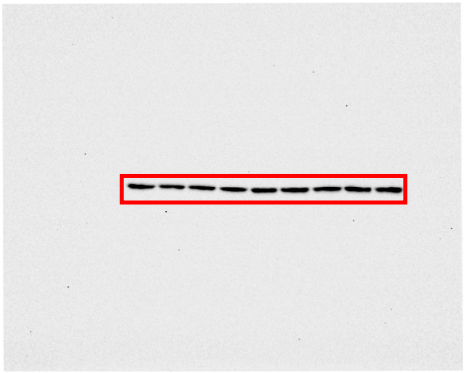

Figure 2b β-actin

Full-length and uncropped western blot for Figure 2

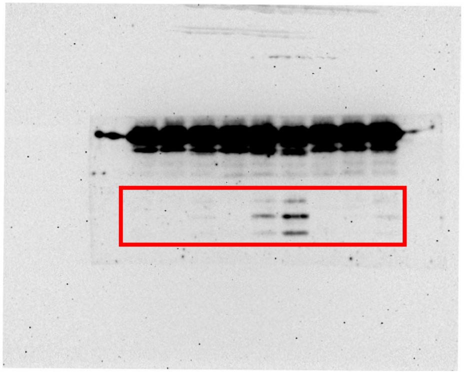

Figure 2c cleaved-caspase-3

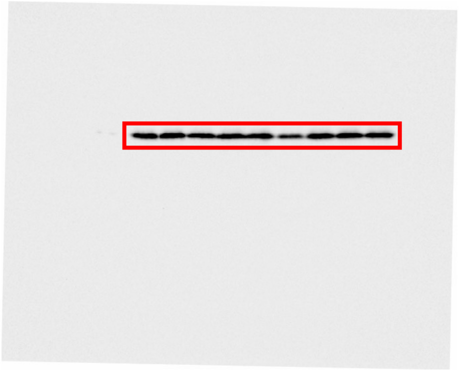

Figure 2c pro-caspase-3

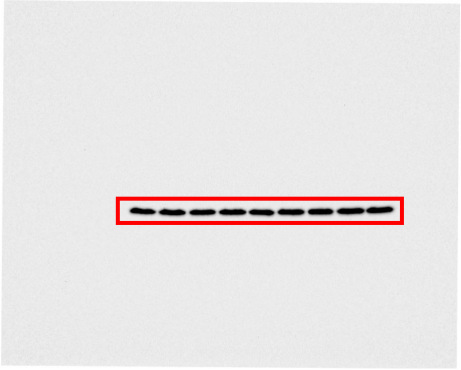

Figure 2c β-actin

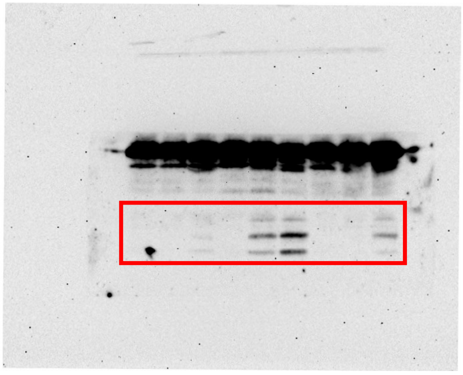

Figure 2d cleaved-caspase-3

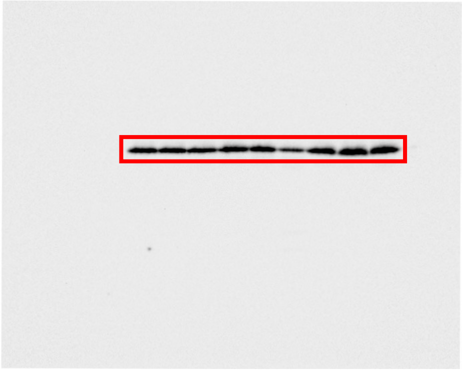

Figure 2d pro-caspase-3

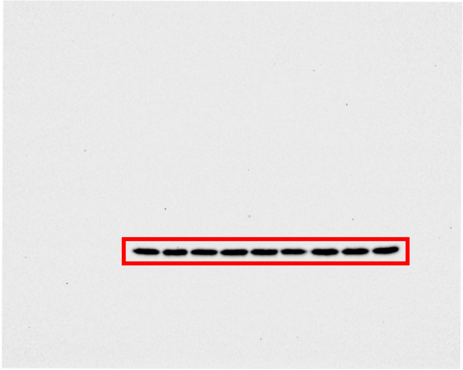

Figure 2d β-actin

Full-length and uncropped western blot for Figure 3

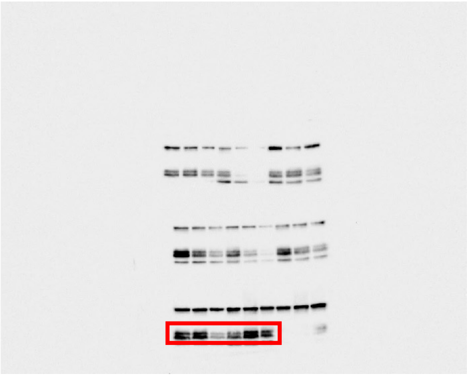

Figure 3a MDM2

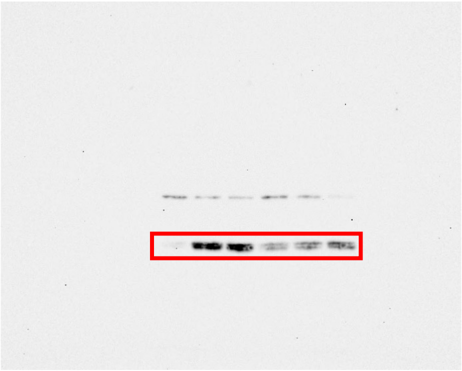

Figure 3a p53

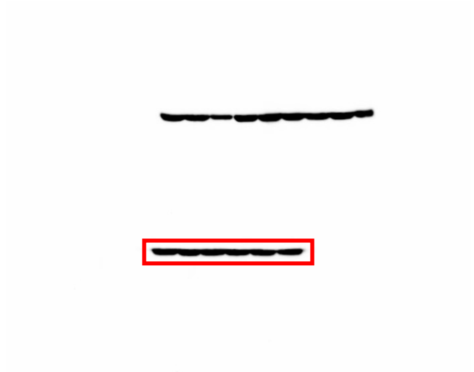

Figure 3a β-actin

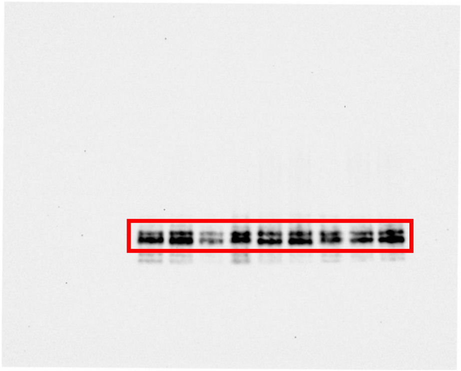

Figure 3b MDM2

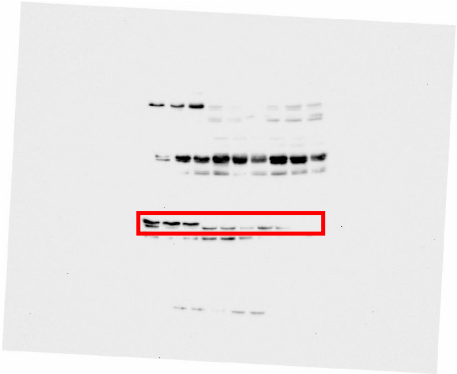

Figure 3b MKRN1

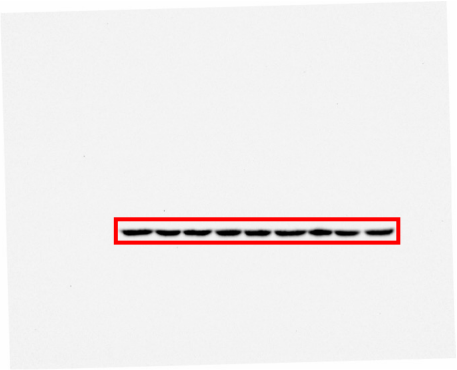

Figure 3b β-actin

Full-length and uncropped western blot for Figure 3

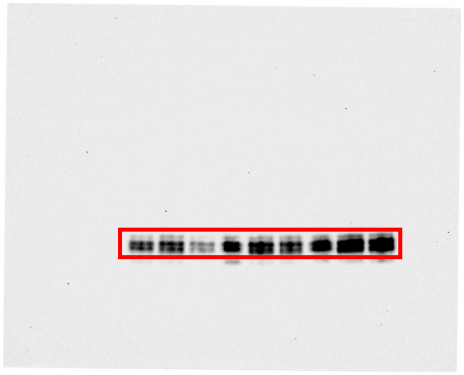

Figure 3c MDM2

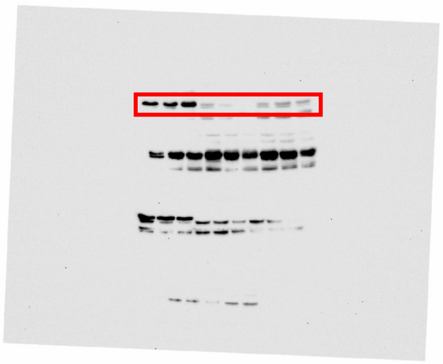

Figure 3c MKRN1

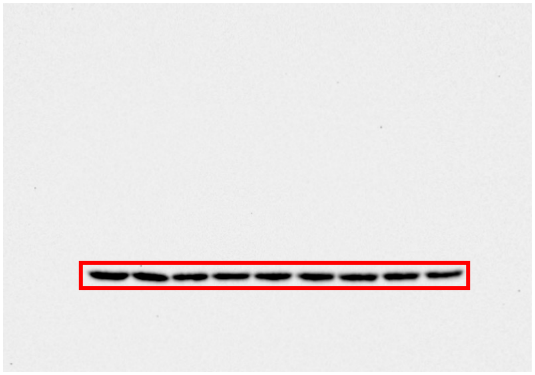

Figure 3c β-actin

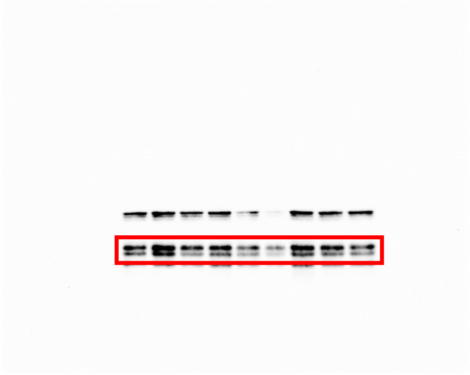

Figure 3d MDM2

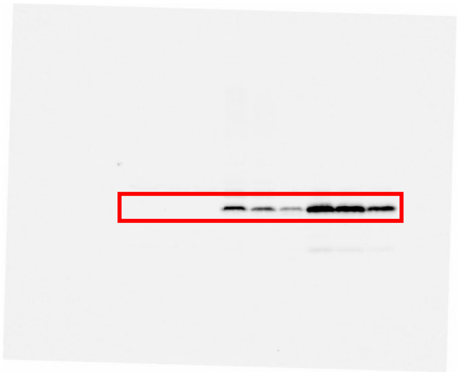

Figure 3d MKRN1

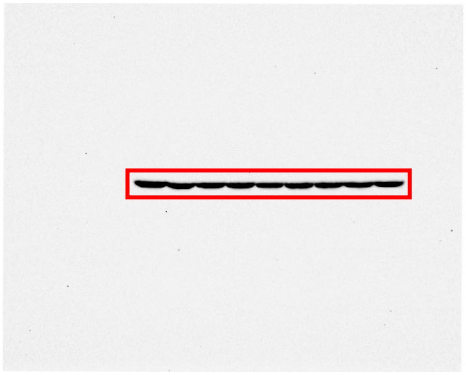

Figure 3d β-actin

Full-length and uncropped western blot for Figure 3

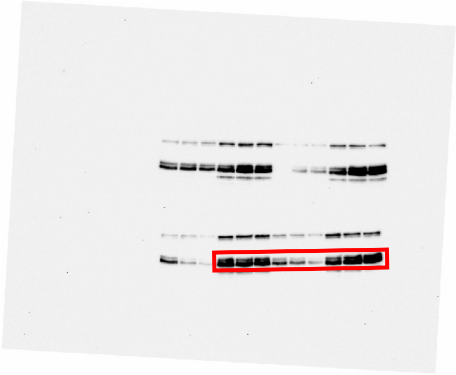

Figure 3e MDM2

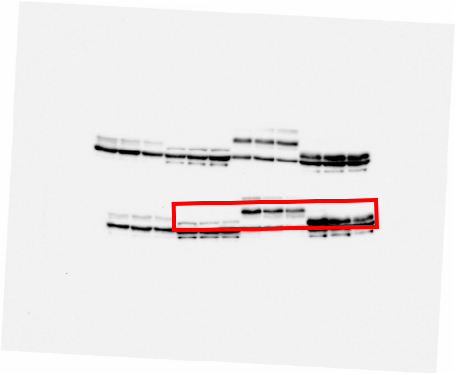

Figure 3e MKRN1

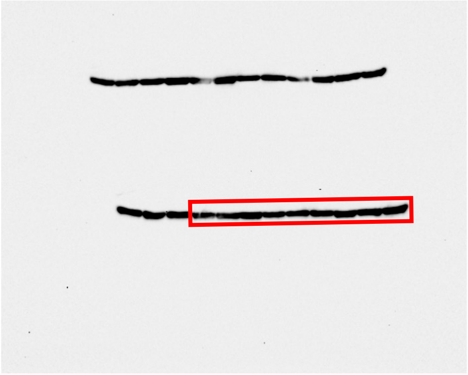

Figure 3e β-actin

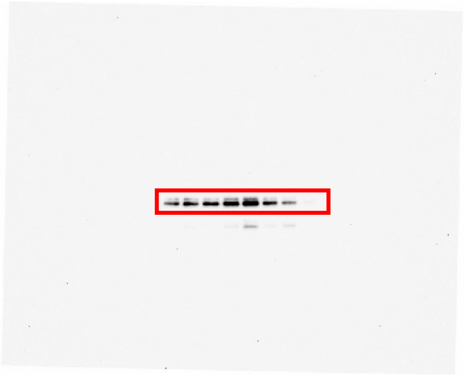

Figure 3f Myc

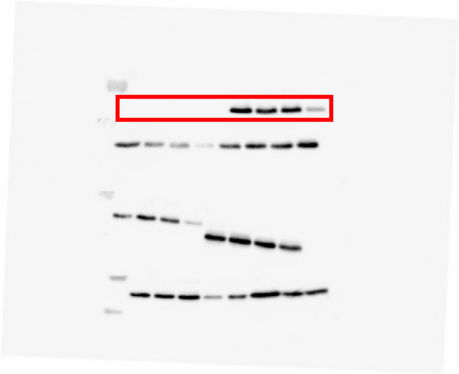

Figure 3f FLAG

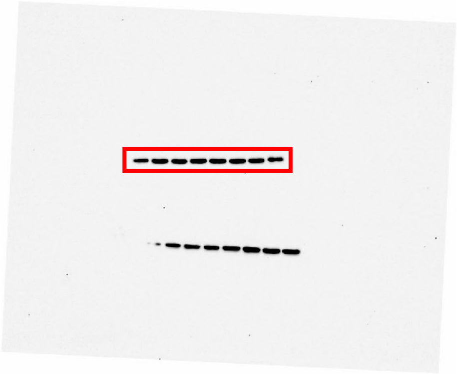

Figure 3f β-actin

Full-length and uncropped western blot for Figure 3

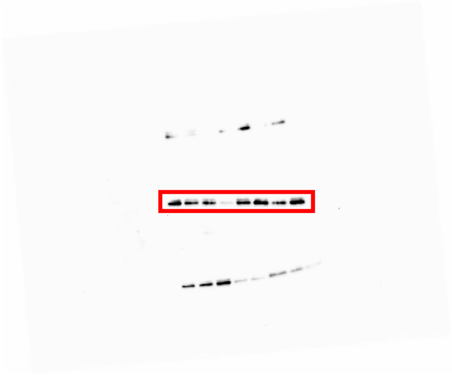

Figure 3g Myc

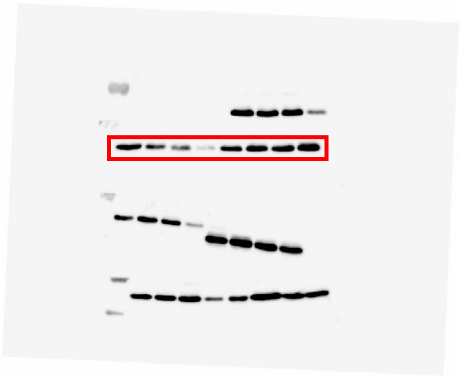

Figure 3g FLAG

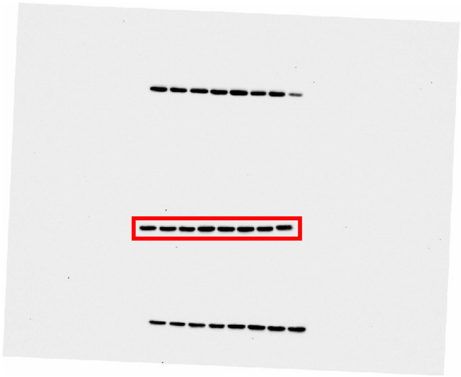

Figure 3g β-actin

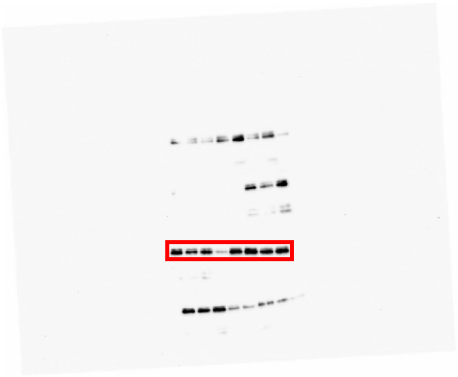

Figure 3h Myc

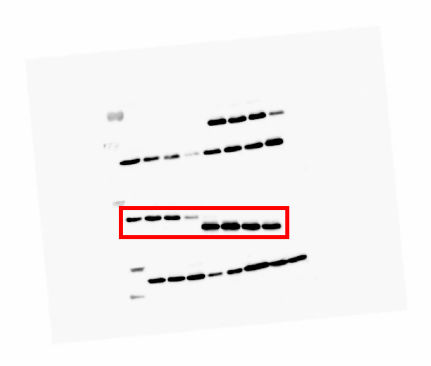

Figure 3h FLAG

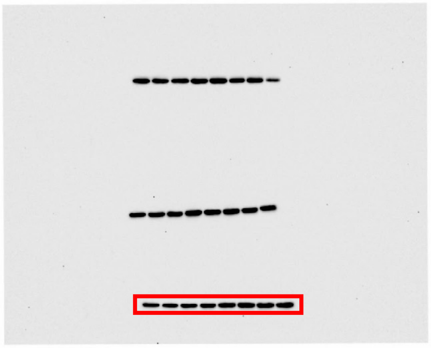

Figure 3h β-actin

Full-length and uncropped western blot for Figure 4

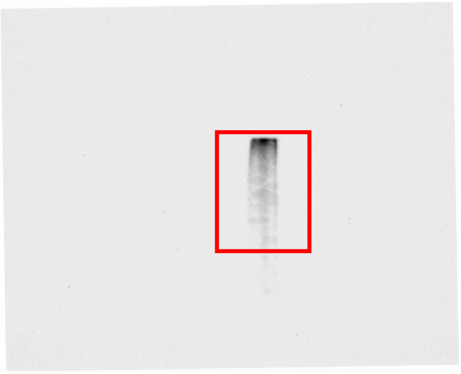

Figure 4a K48-Ub

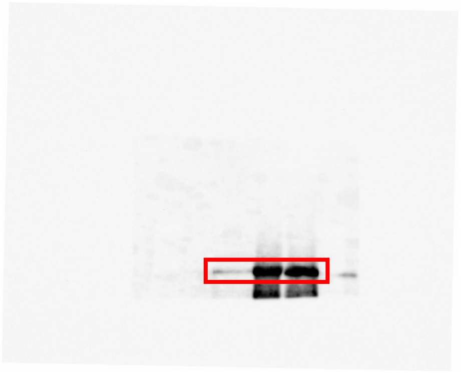

Figure 4a GST

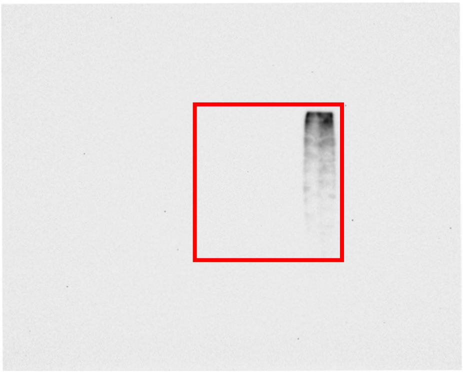

Figure 4b K48-Ub

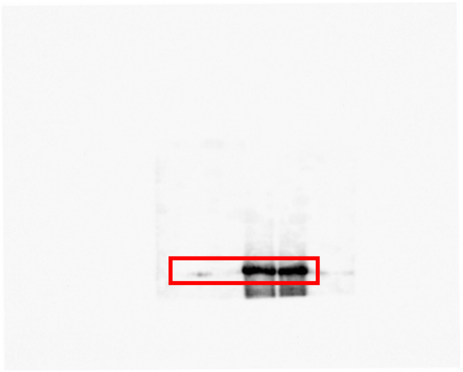

Figure 4b GST

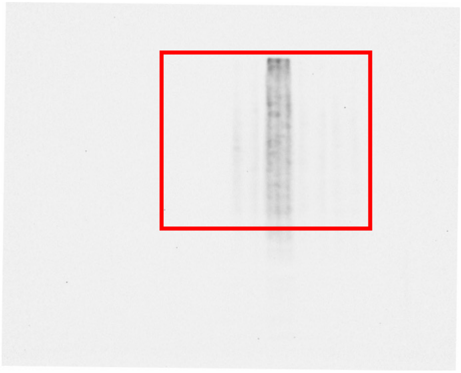

Figure 4c K48-Ub

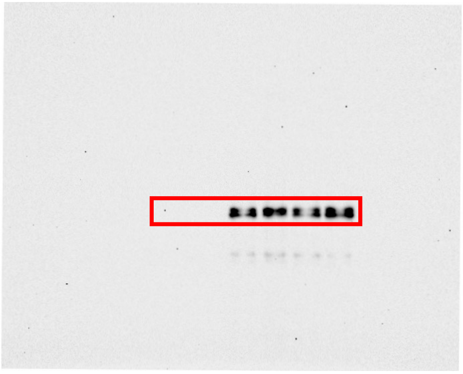

Figure 4c Myc

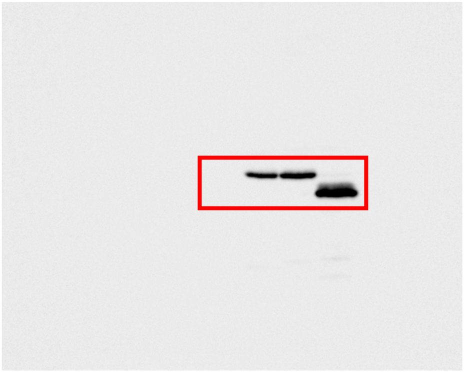

Figure 4c FLAG

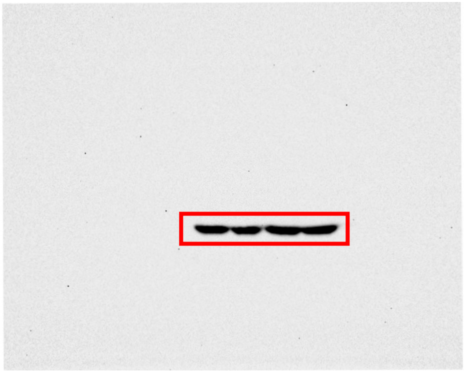

Figure 4c β-actin

Full-length and uncropped western blot for Figure 4

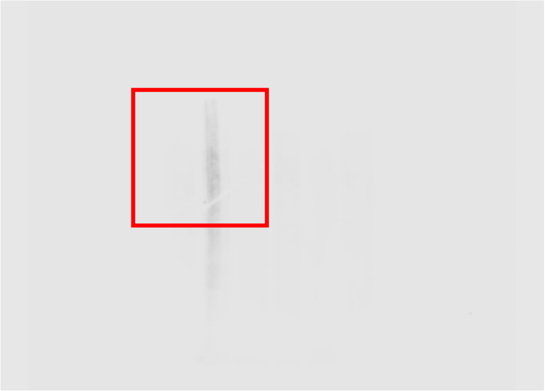

Figure 4d K48-Ub

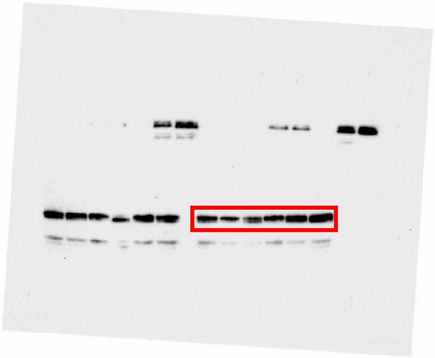

Figure 4d Myc (IP)

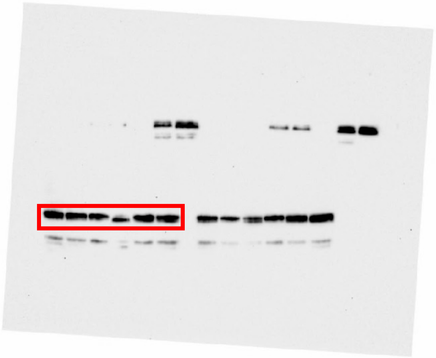

Figure 4d Myc (Input)

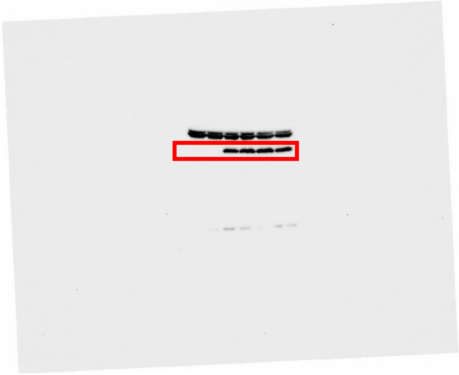

Figure 4d FLAG

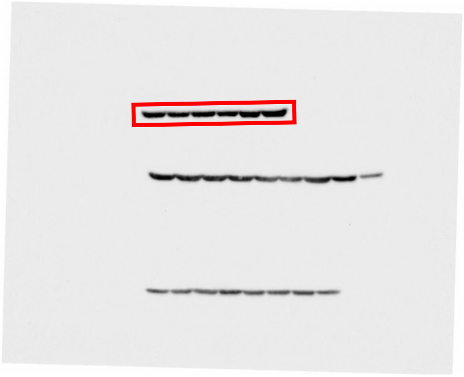

Figure 4d β-actin

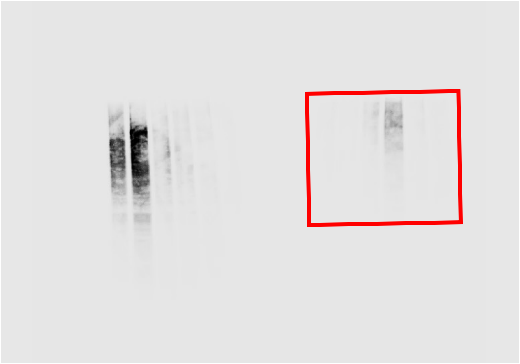

Figure 4e K48-Ub

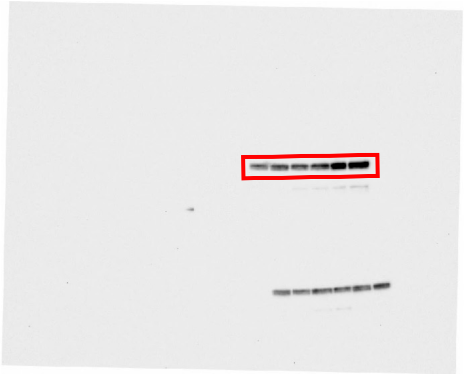

Figure 4e Myc (IP)

Full-length and uncropped western blot for Figure 4

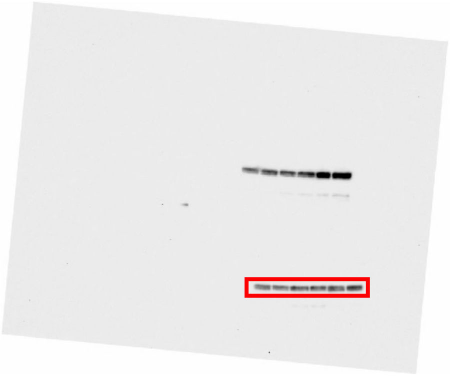

Figure 4e Myc (Input)

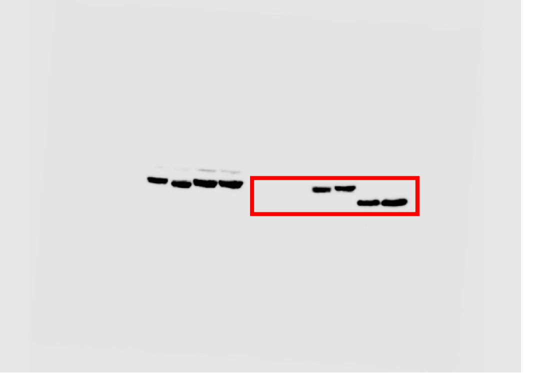

Figure 4e FLAG

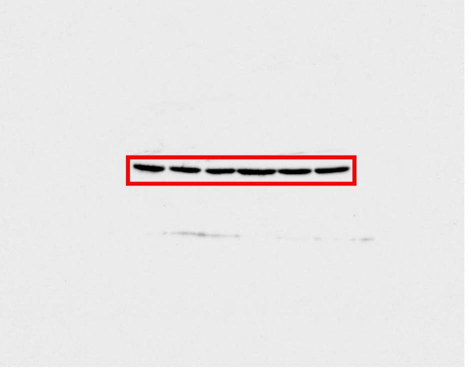

Figure 4e β-actin

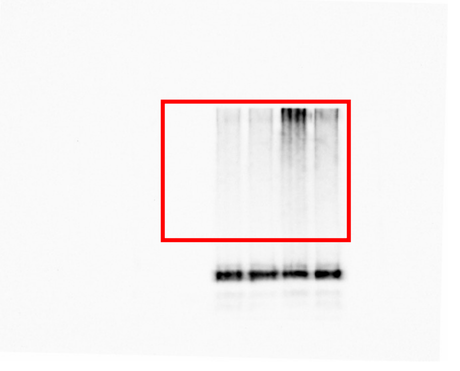

Figure 4f K48-Ub

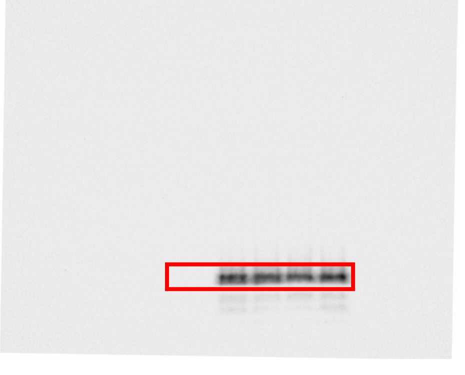

Figure 4f MDM2 (IP)

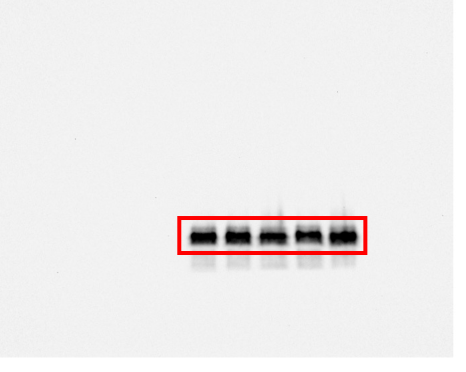

Figure 4f MDM2 (Input)

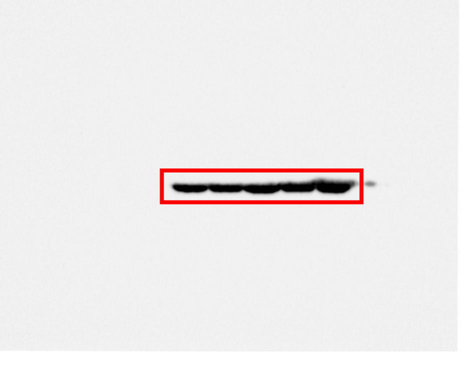

Figure 4f β-actin

Full-length and uncropped western blot for Figure 4

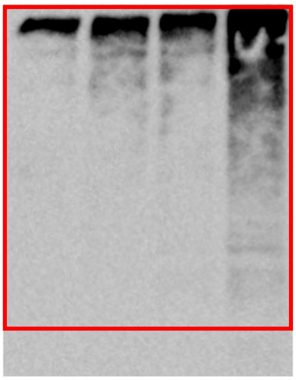

Figure 4g K48-Ub

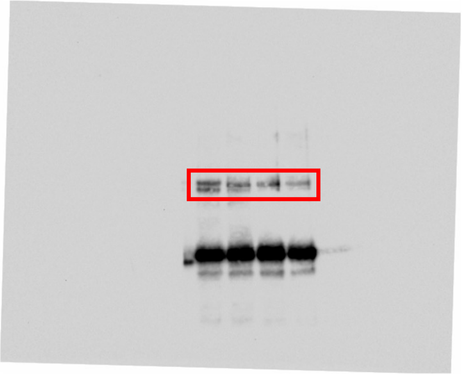

Figure 4g MDM2 (IP)

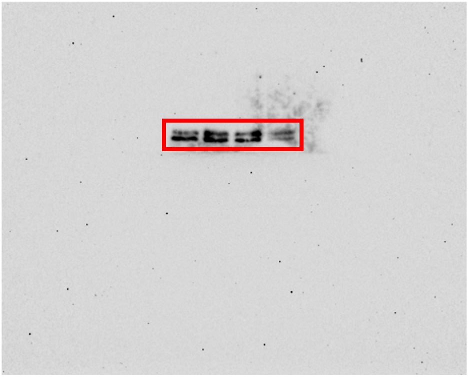

Figure 4g MDM2 (Input)

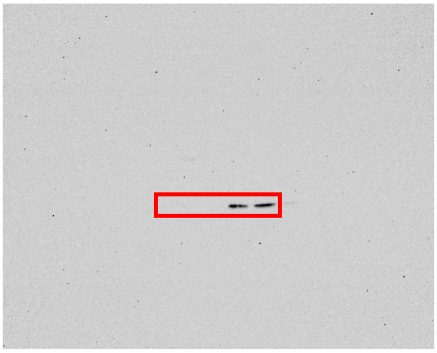

Figure 4g Myc

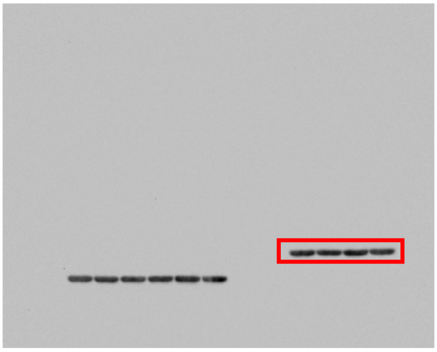

Figure 4g β-actin

Full-length and uncropped western blot for Figure 5

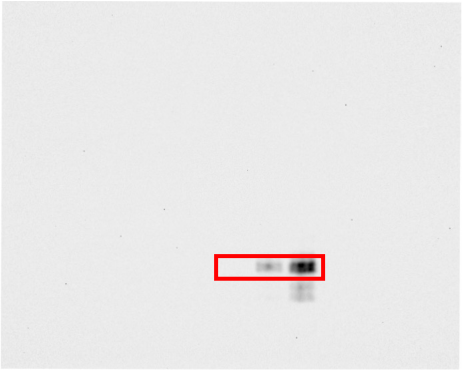

Figure 5a MDM2 (IP)

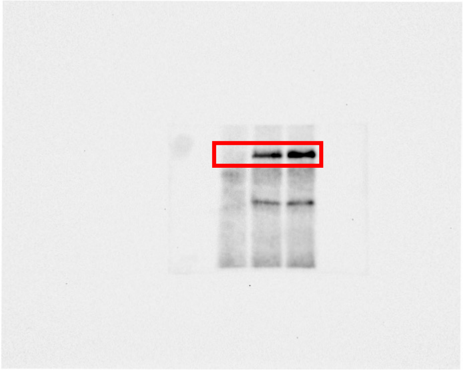

Figure 5a MKRN1 (IP)

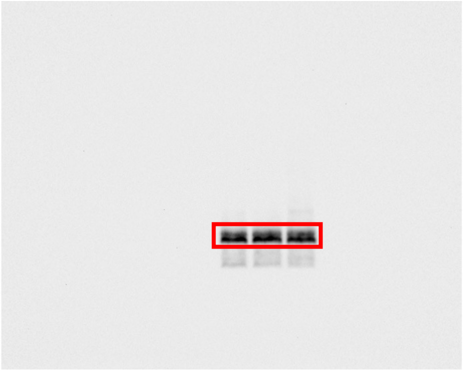

Figure 5a MDM2 (Input)

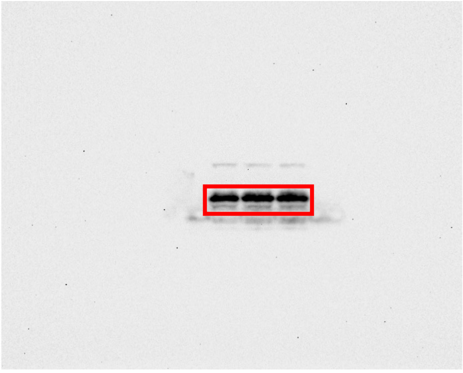

Figure 5a MKRN1 (Input)

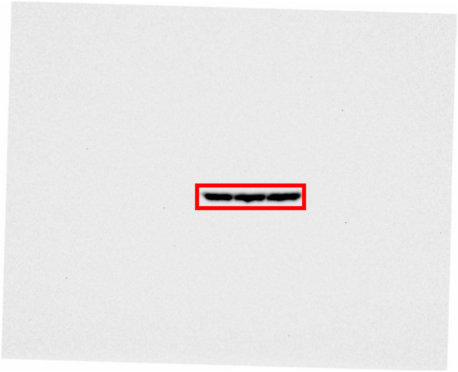

Figure 5a β-actin

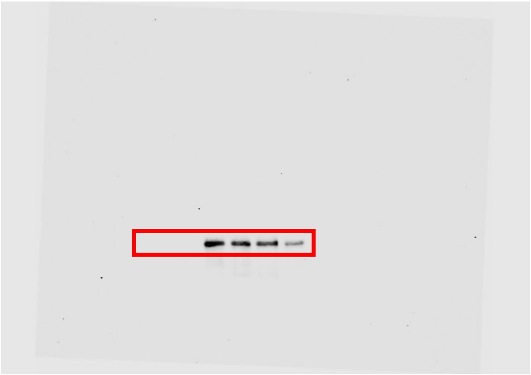

Figure 5b FLAG (IP)

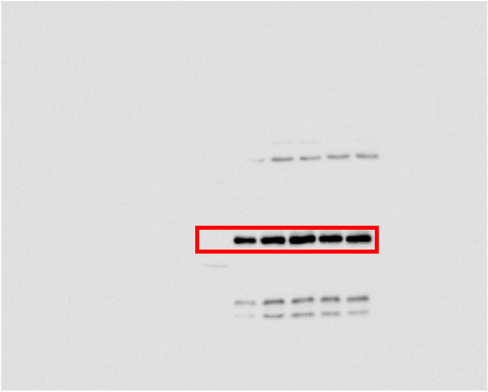

Figure 5b Myc (IP)

Full-length and uncropped western blot for Figure 5

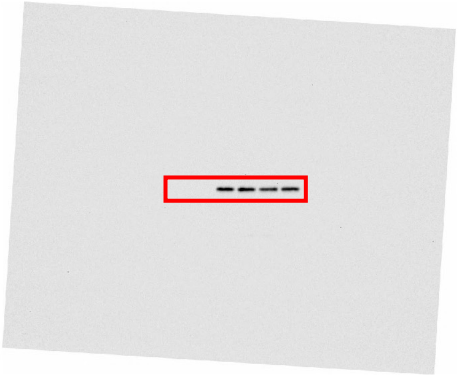

Figure 5b FLAG (Input)

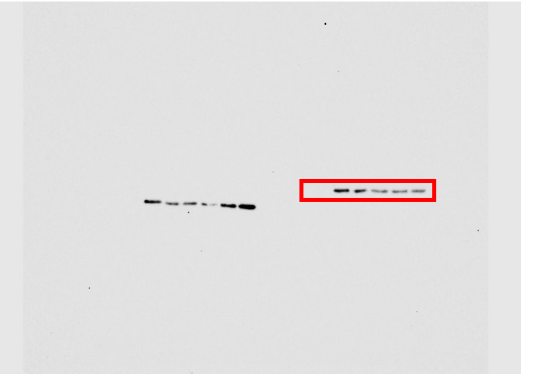

Figure 5b Myc (Input)

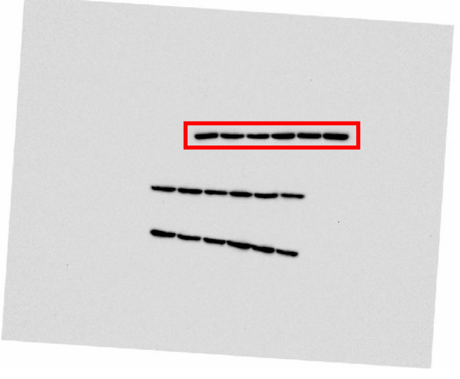

Figure 5b β-actin

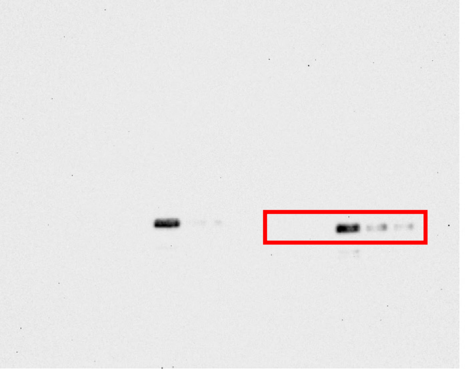

Figure 5c Myc (IP)

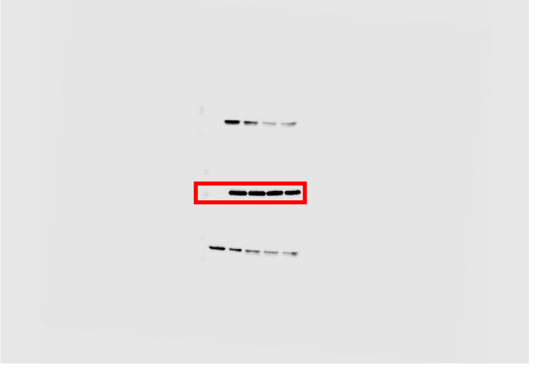

Figure 5c FLAG (IP)

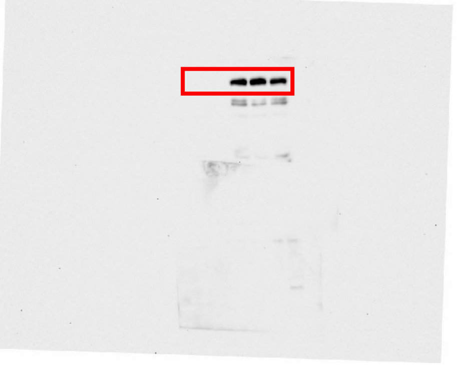

Figure 5c Myc (Input)

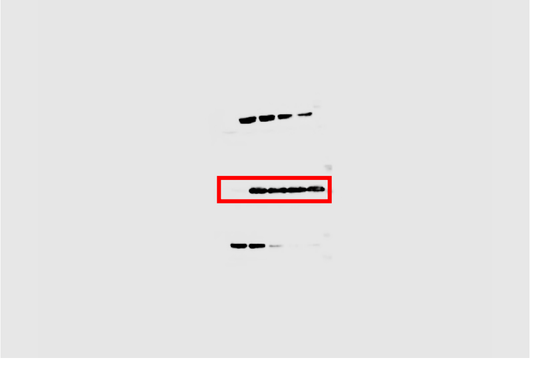

Figure 5c FLAG (Input)

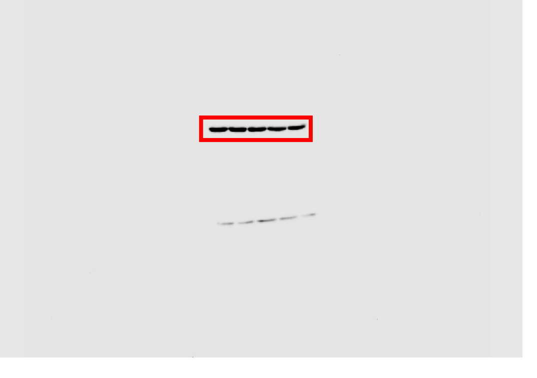

Figure 5c β-actin

Full-length and uncropped western blot for Figure 5

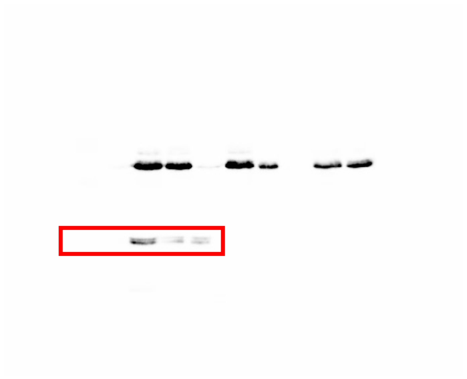

Figure 5d FLAG (IP)

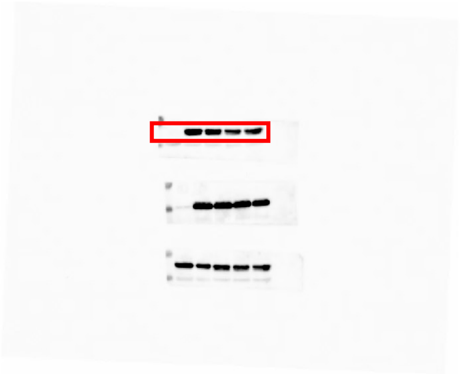

Figure 5d Myc (IP)

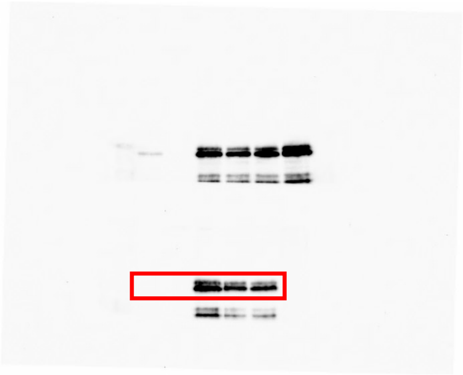

Figure 5d FLAG (Input)

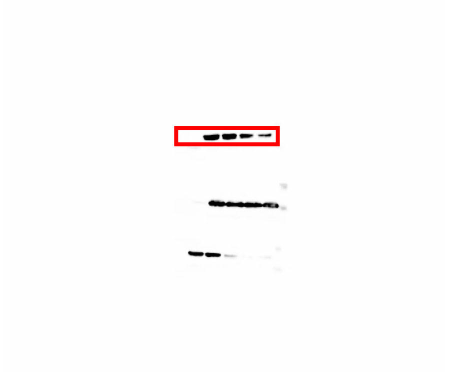

Figure 5d Myc (Input)

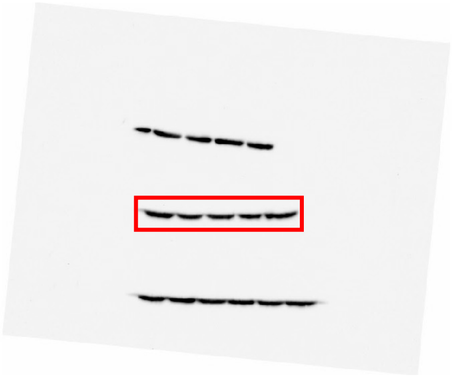

Figure 5d β-actin

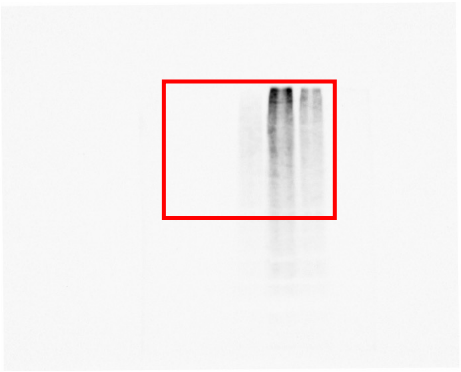

Figure 5e K48-Ub

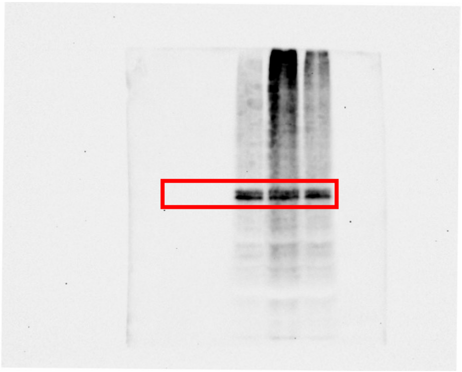

Figure 5e Myc (IP)

Full-length and uncropped western blot for Figure 5

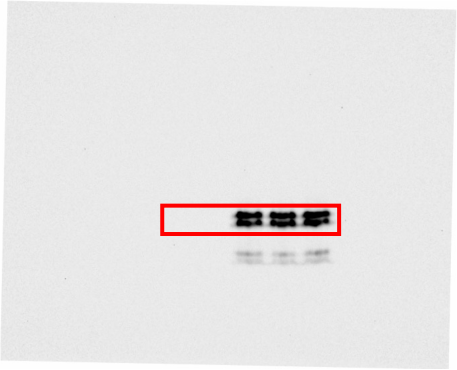

Figure 5e Myc (Input)

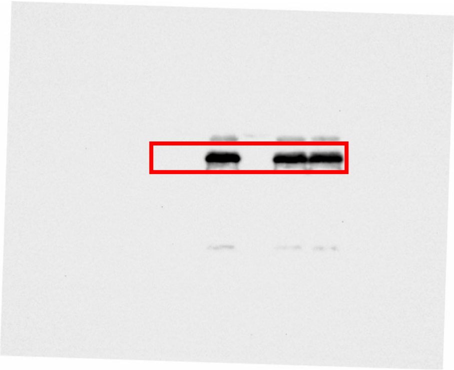

Figure 5e FLAG

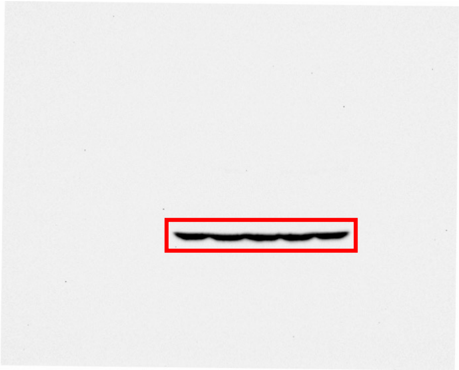

Figure 5e β-actin

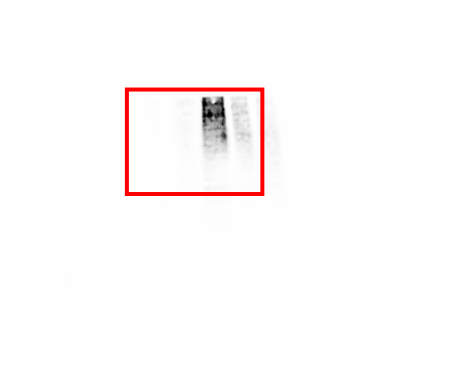

Figure 5f K48-Ub

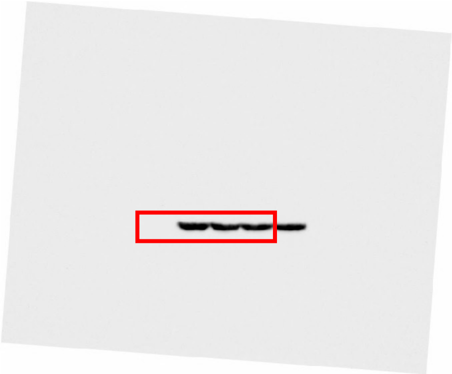

Figure 5f FLAG (IP)

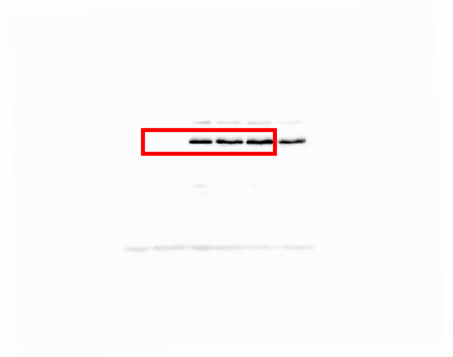

Figure 5f FLAG (Input)

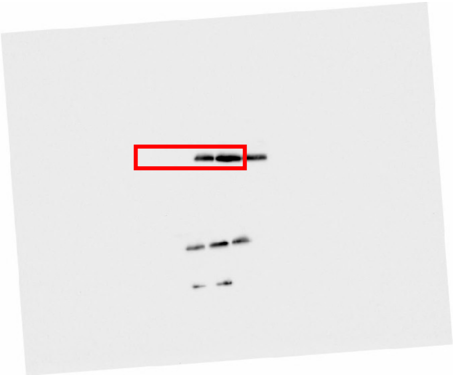

Figure 5f Myc

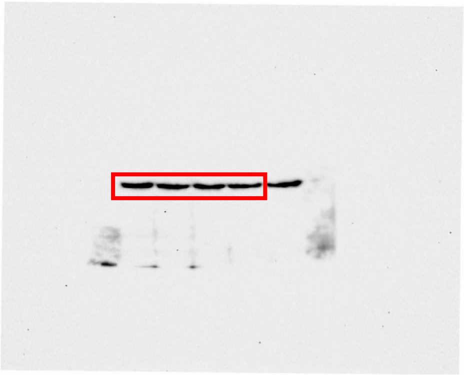

Figure 5f β-actin

Full-length and uncropped western blot for Figure 5

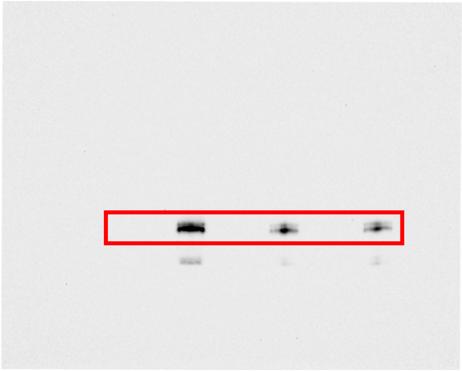

Figure 5g Myc (IP)

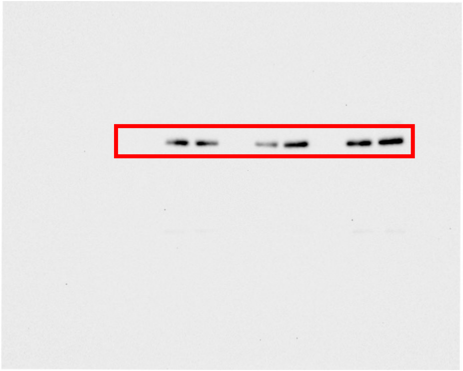

Figure 5g FLAG (IP)

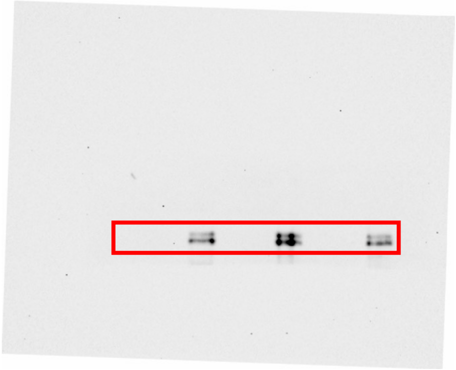

Figure 5g Myc (Input)

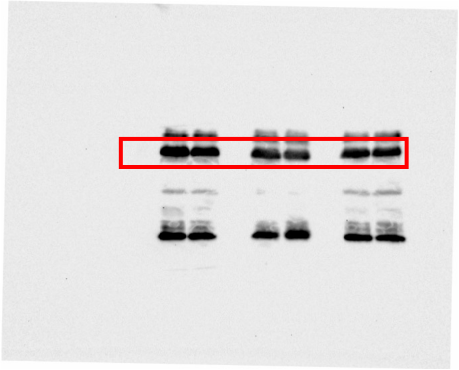

Figure 5g FLAG (Input)

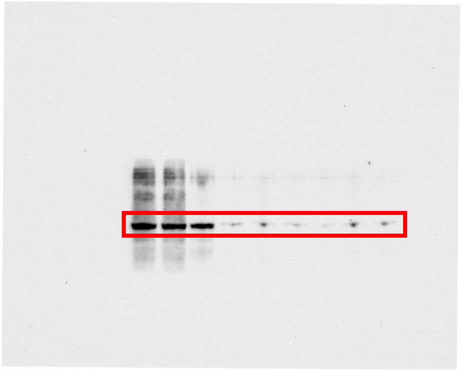

Figure 5g SIRT1

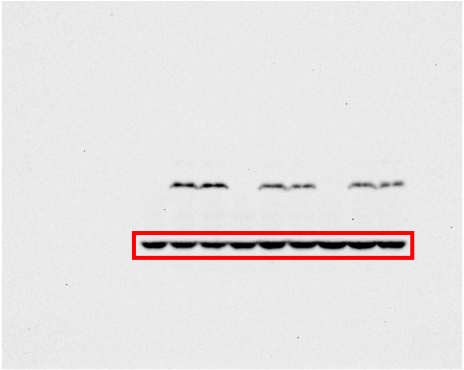

Figure 5g β-actin

Full-length and uncropped western blot for Figure 5

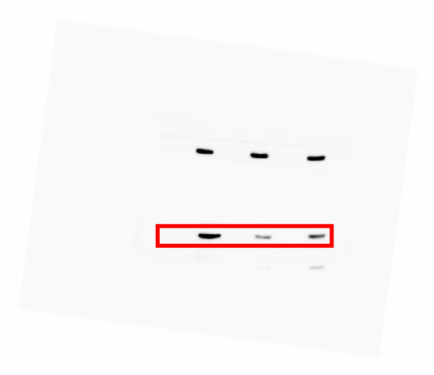

Figure 5h Myc (IP)

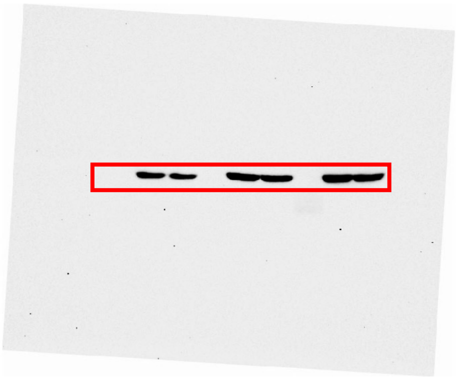

Figure 5h FLAG (IP)

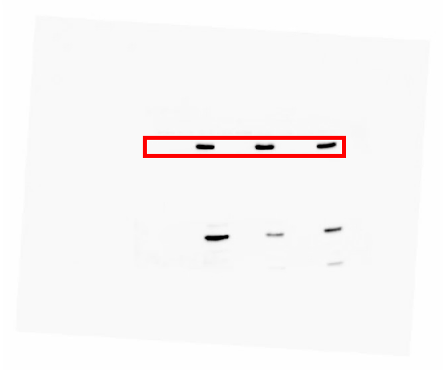

Figure 5h Myc (Input)

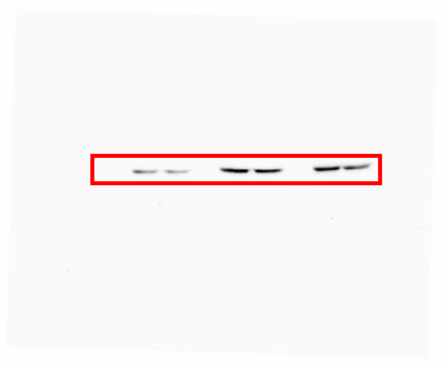

Figure 5h FLAG (Input)

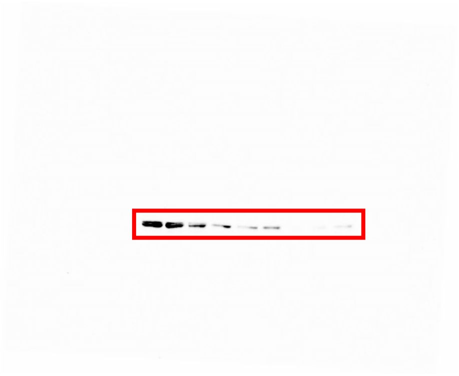

Figure 5h SIRT1

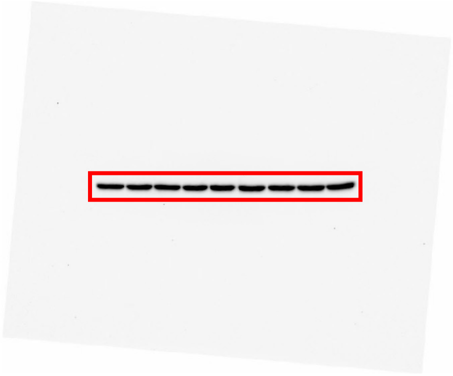

Figure 5h β-actin

Full-length and uncropped western blot for Figure 5

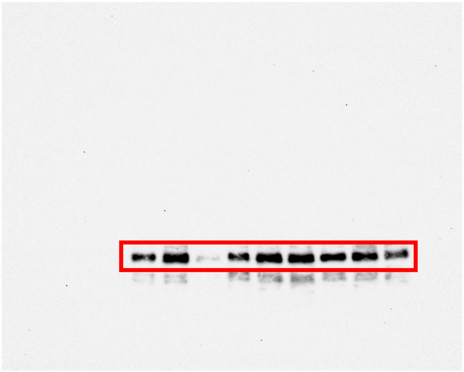

Figure 5i MDM2

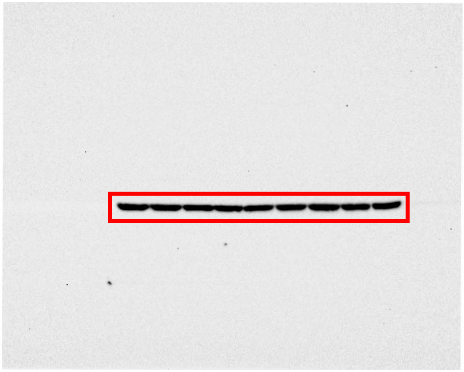

Figure 5i β-actin

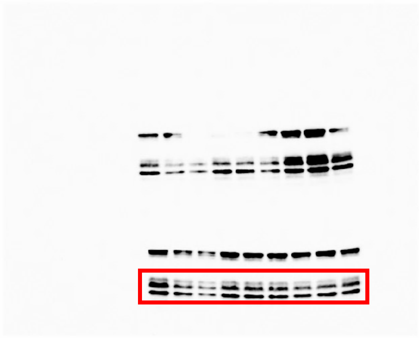

Figure 5j MDM2

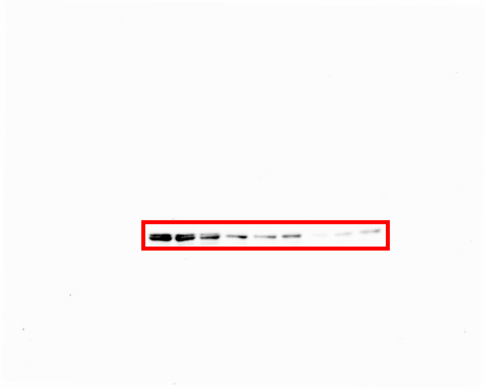

Figure 5j SIRT1

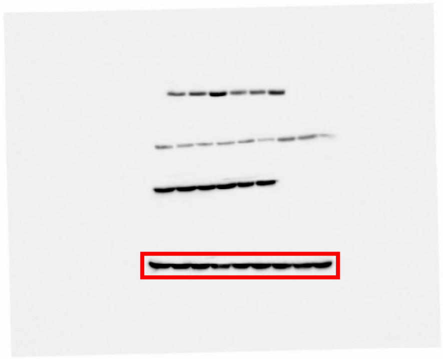

Figure 5j β-actin

Full-length and uncropped western blot for Figure 6

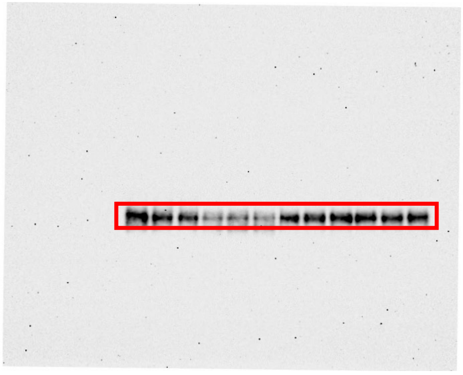
Figure 6g MDM2

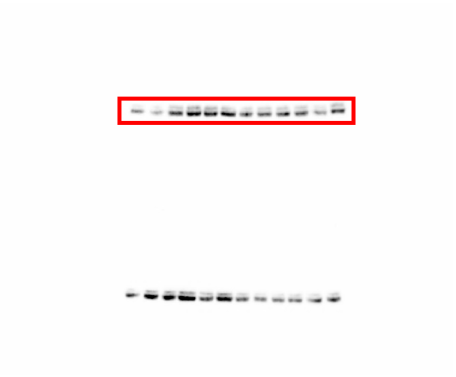
Figure 6g p53

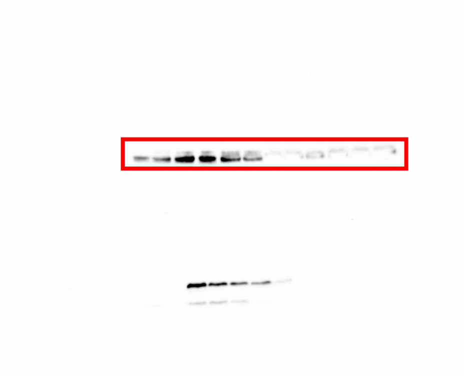
Figure 6g MKRN1

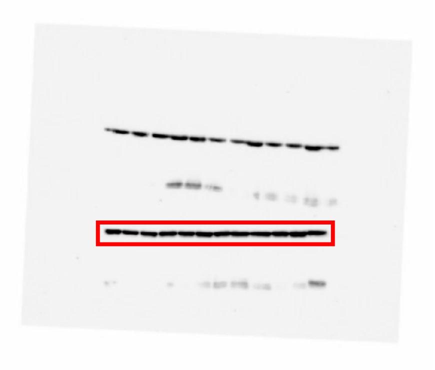
Figure 6g β-actin
