# Supplementary material for: Identification of MKRN1 as a key modulator of the p53-MDM2 feedback loop
Source: Cell Death Differ. 2026 Jan 30;33(7):1474–87. doi: 10.1038/s41418-026-01662-4 (PMC13342678; doi:10.1038/s41418-026-01662-4)
Supplement: Supplementary file 3 — Full and uncropped WB (Extended data figures) [file 41418_2026_1662_MOESM3_ESM.pdf]

Full-length and uncropped western blot for Extended Data Figure 1

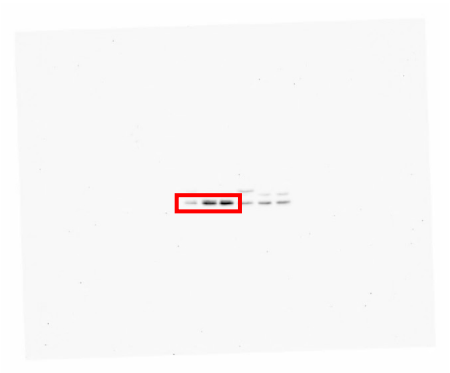

Figure 1a p53

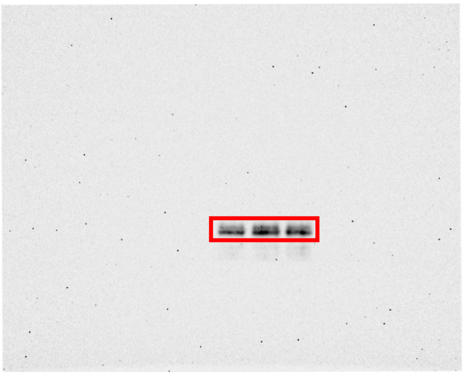

Figure 1a MDM2

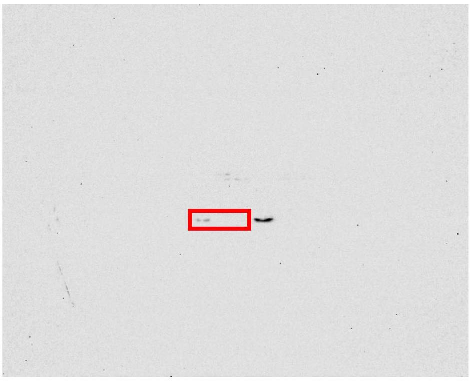

Figure 1a MKRN1

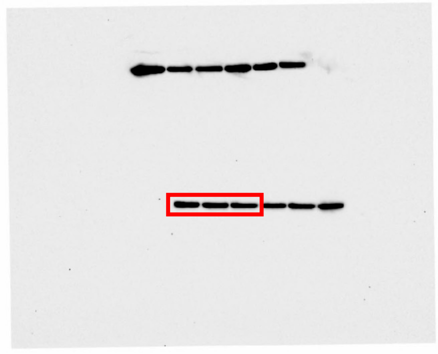

Figure 1a β-actin

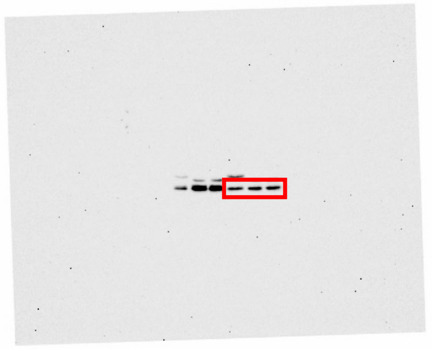

Figure 1b p53

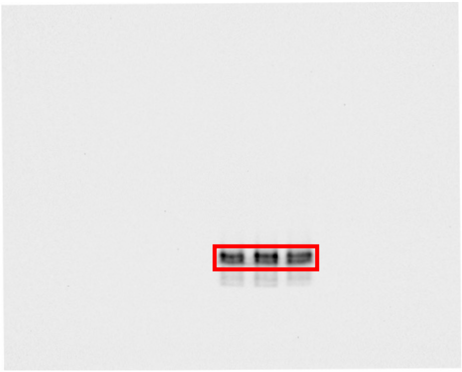

Figure 1b MDM2

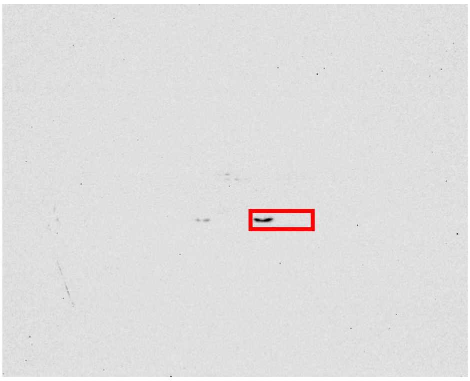

Figure 1b MKRN1

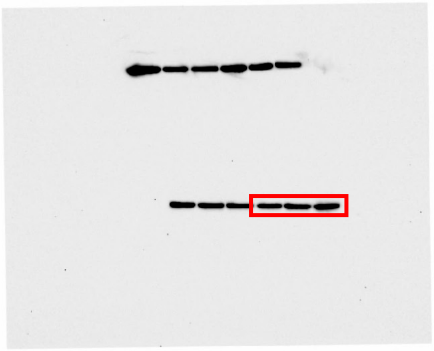

Figure 1b β-actin

Full-length and uncropped western blot for Extended Data Figure 1

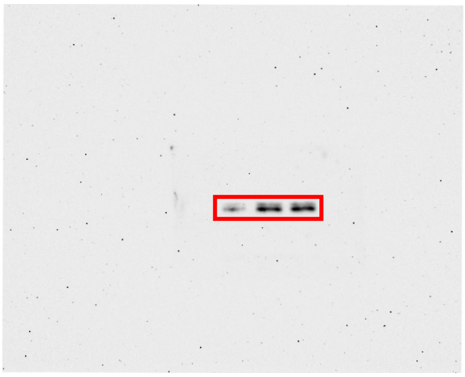

Figure 1c p53

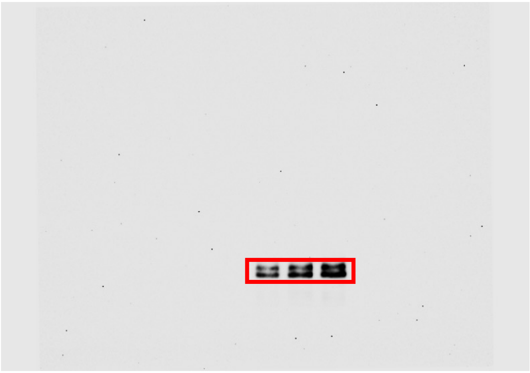

Figure 1c MDM2

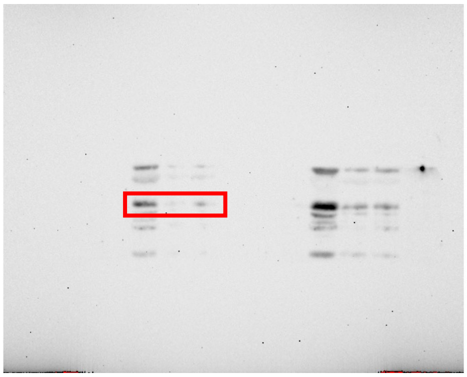

Figure 1c MKRN1

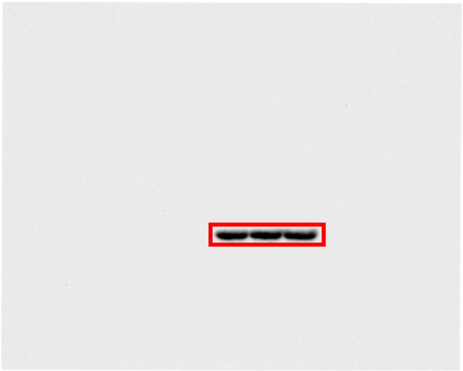

Figure 1c β-actin

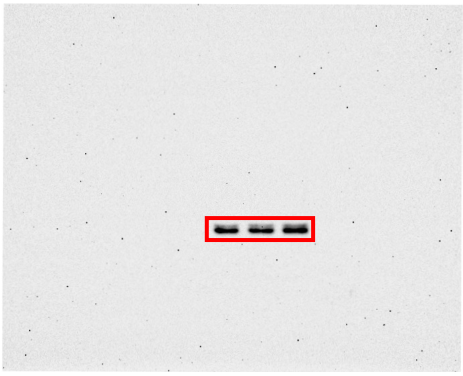

Figure 1d p53

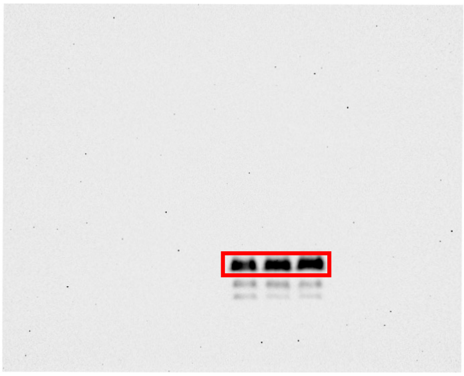

Figure 1d MDM2

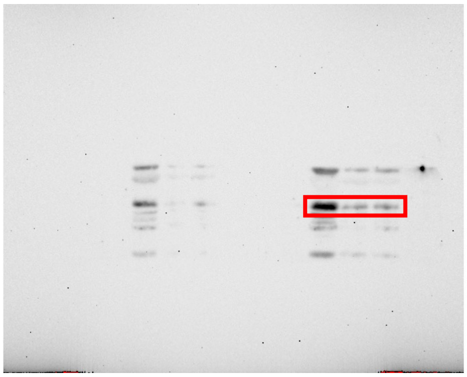

Figure 1d MKRN1

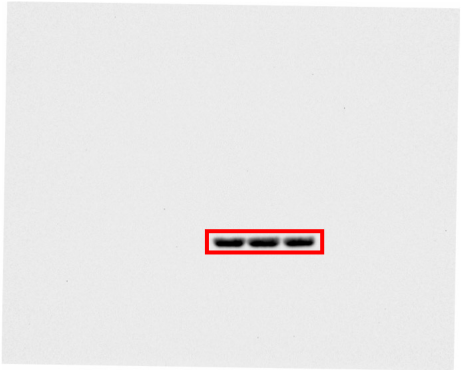

Figure 1d β-actin

Full-length and uncropped western blot for Extended Data Figure 1

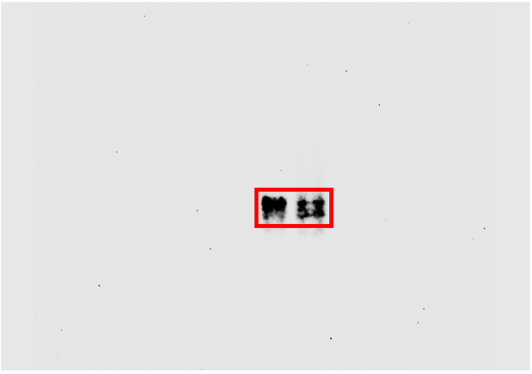

Figure 1e p53 (IP)

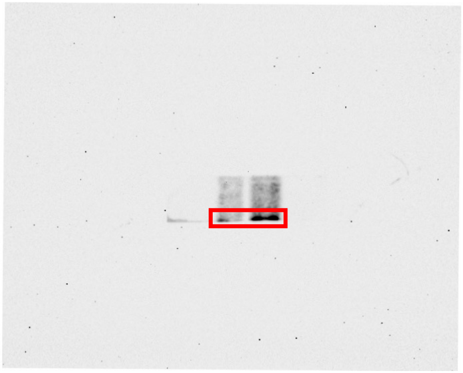

Figure 1e MKRN1 (IP)

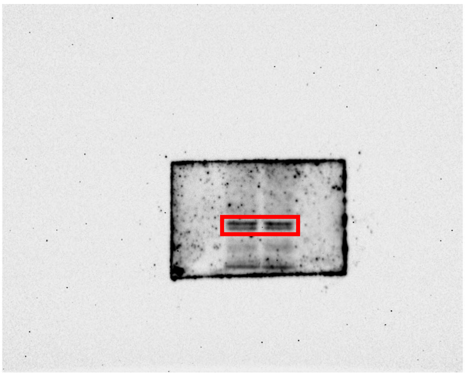

Figure 1e p53 (Input)

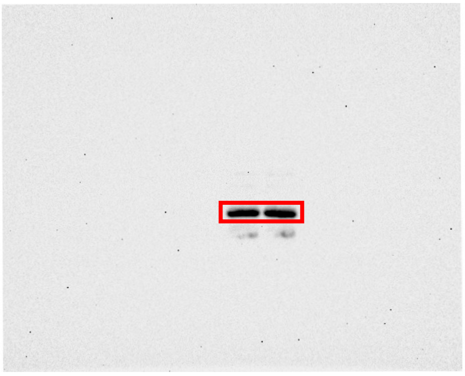

Figure 1e MKRN1 (Input)

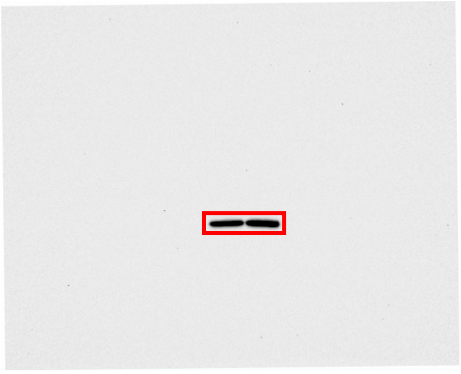

Figure 1e β-actin

Full-length and uncropped western blot for Extended Data Figure 1

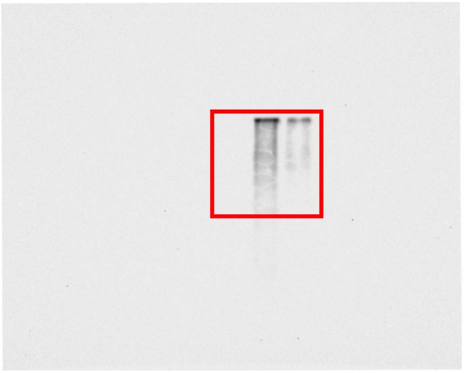

Figure 1f K48-Ub

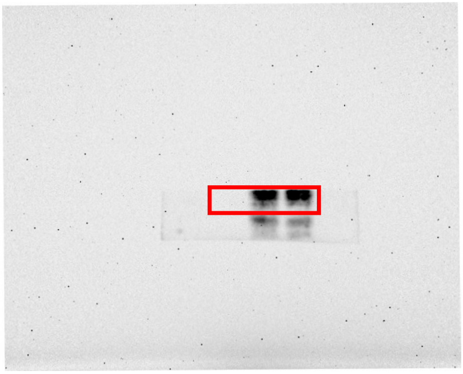

Figure 1f p53 (IP)

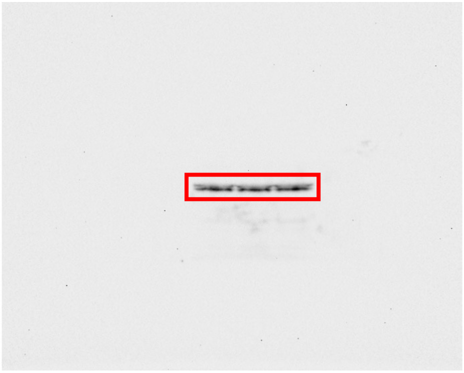

Figure 1f p53 (Input)

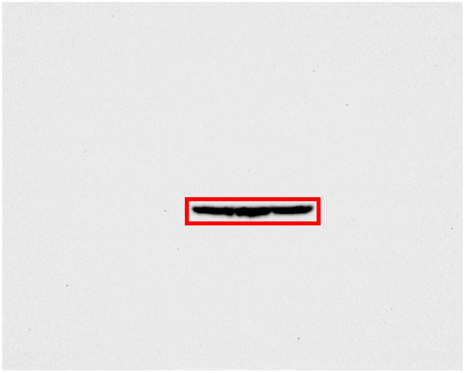

Figure 1f β-actin

Full-length and uncropped western blot for Extended Data Figure 2

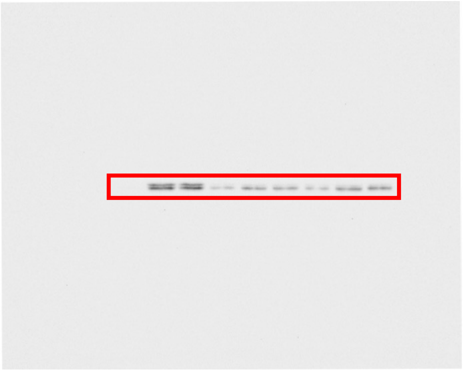

Figure 2a p53

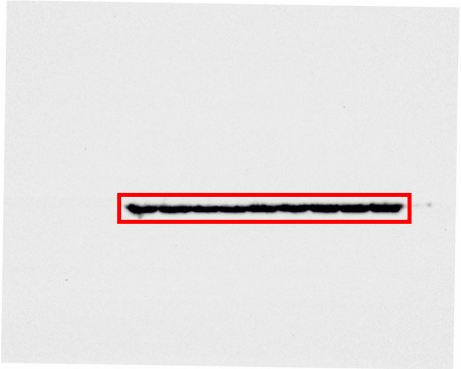

Figure 2a β-actin

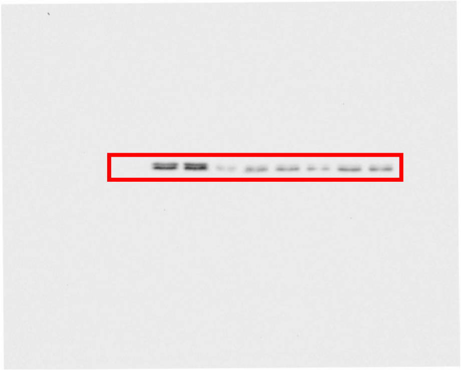

Figure 2b p53

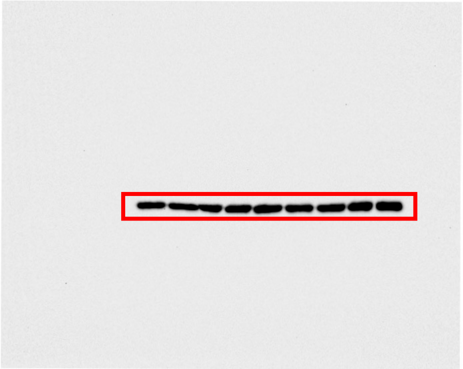

Figure 2b β-actin

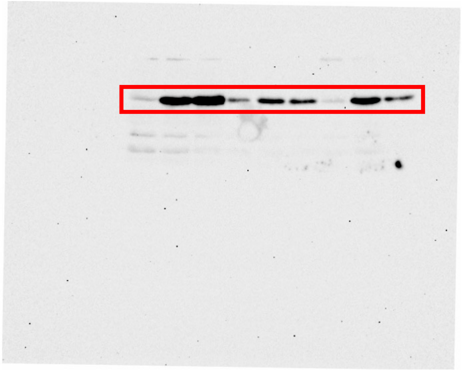

Figure 2c p53

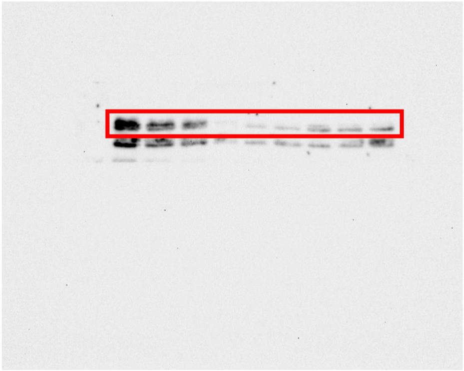

Figure 2c MKRN1

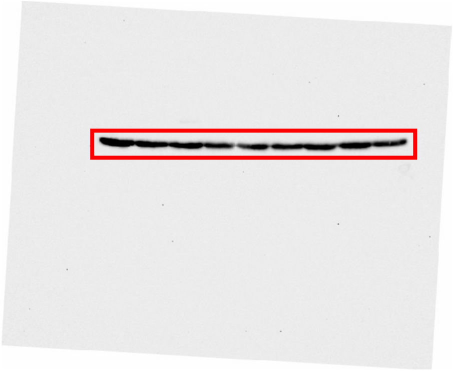

Figure 2c β-actin

Full-length and uncropped western blot for Extended Data Figure 2・3

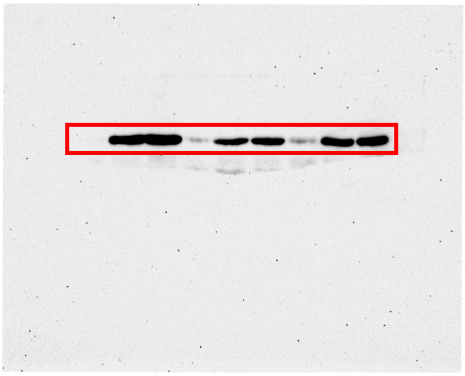

Figure 2d p53

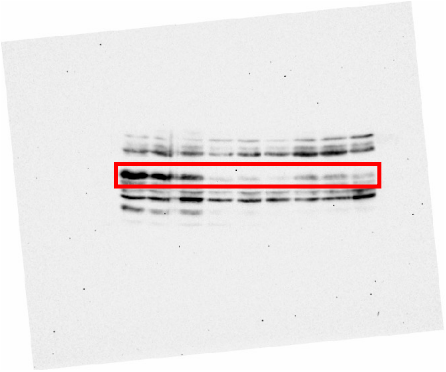

Figure 2d MKRN1

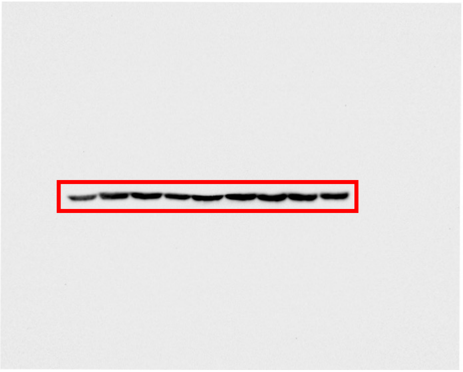

Figure 2d β-actin

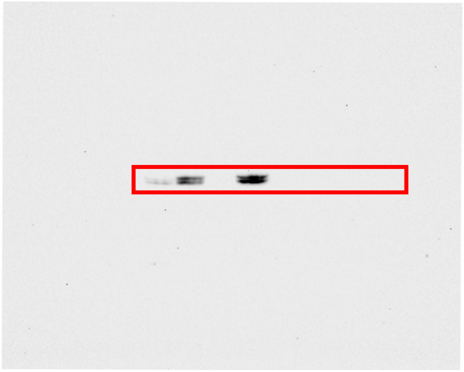

Figure 3d p53

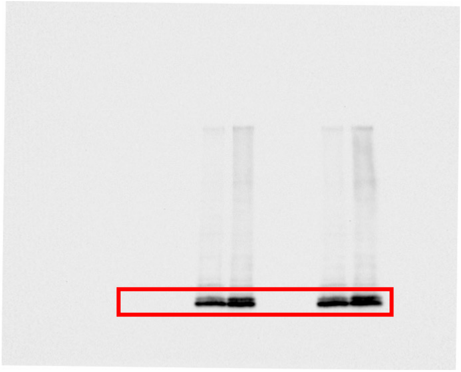

Figure 3d FLAG

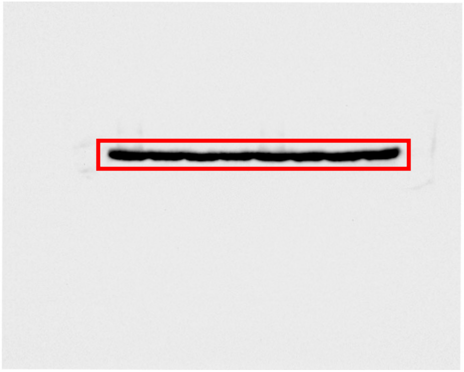

Figure 3d β-actin

Full-length and uncropped western blot for Extended Data Figure 6

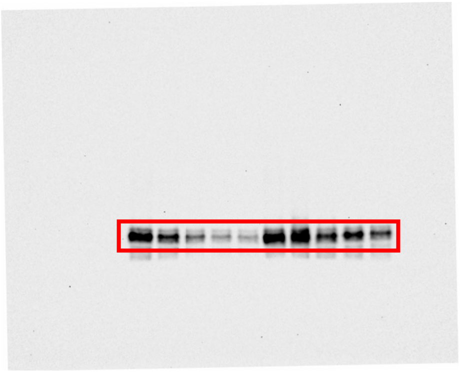

Figure 6b MDM2

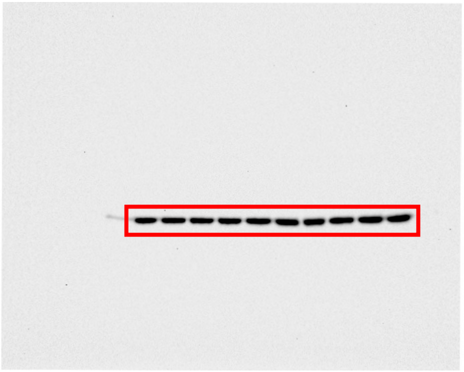

Figure 6b β-actin

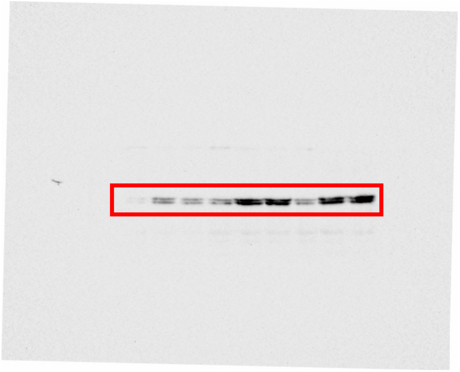

Figure 6c p53

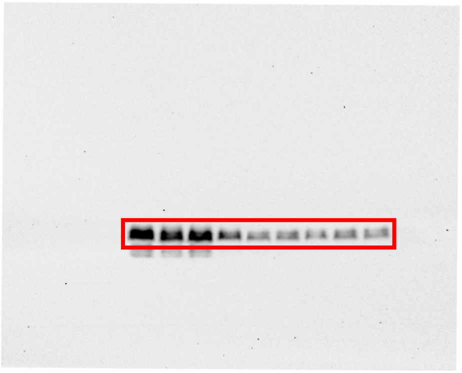

Figure 6c MDM2

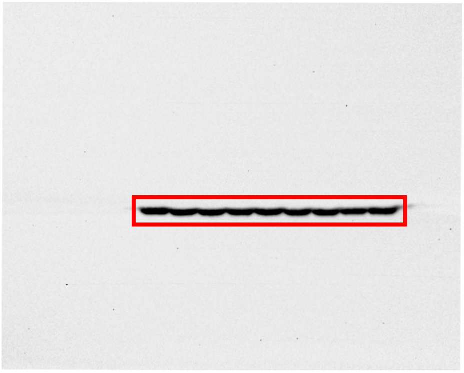

Figure 6c β-actin

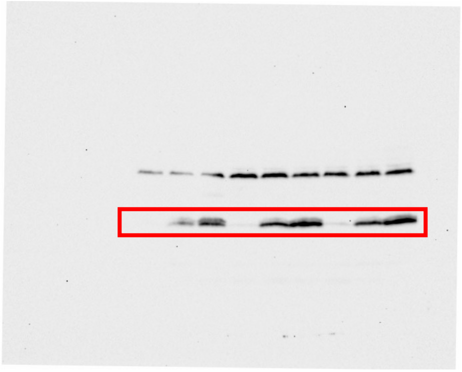

Figure 6e p53

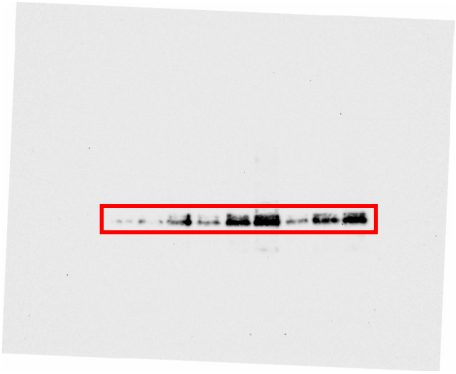

Figure 6e MDM2

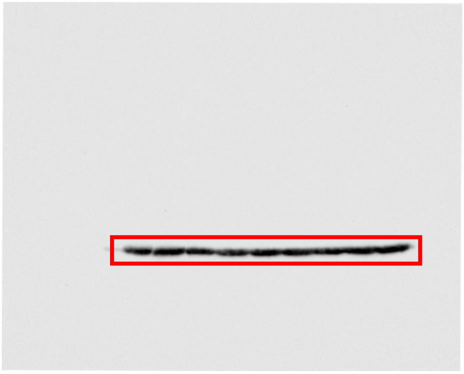

Figure 6e β-actin

Full-length and uncropped western blot for Extended Data Figure 7

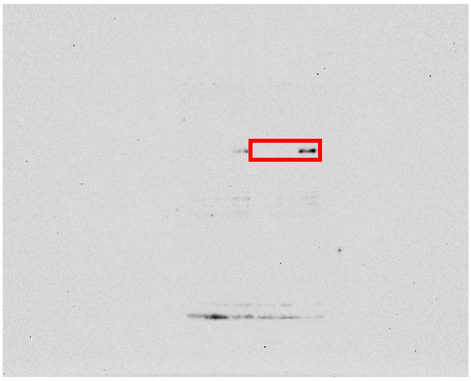

Figure 7a Myc (IP)

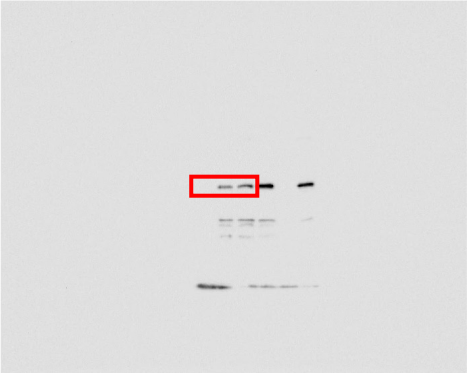

Figure 7a FLAG (IP)

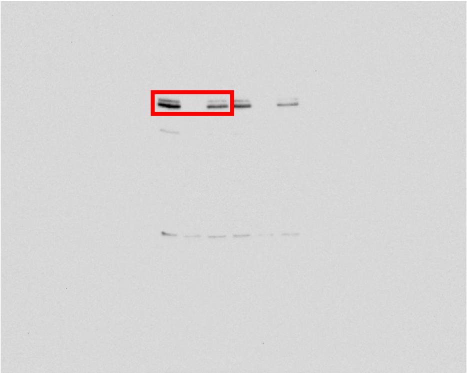

Figure 7a Myc (Input)

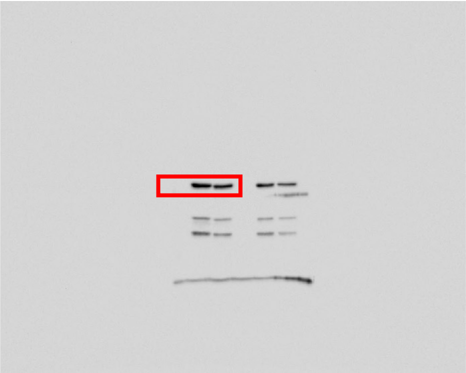

Figure 7a FLAG (Input)

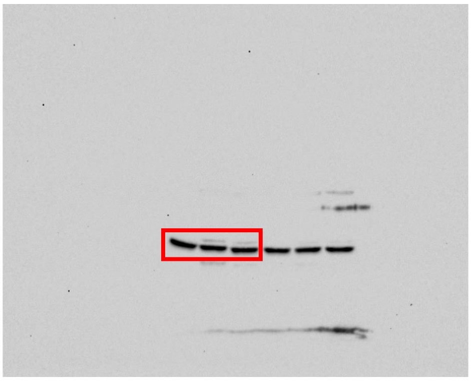

Figure 7a β-actin

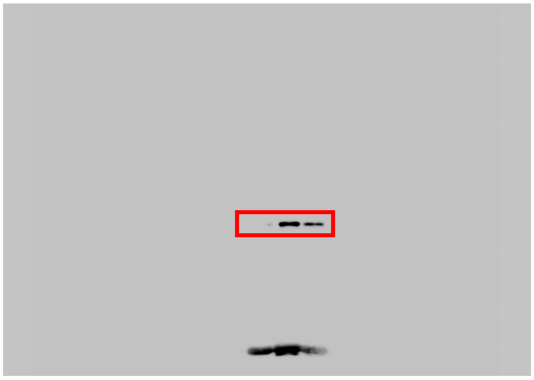

Figure 7b Myc (IP)

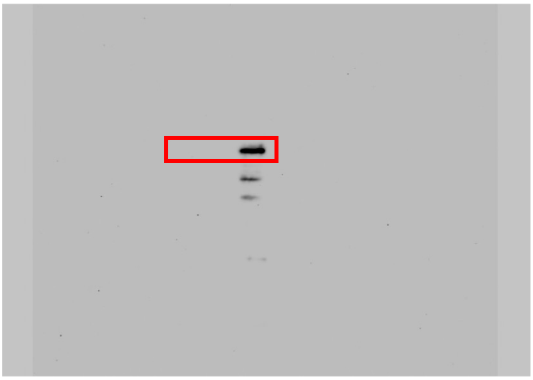

Figure 7b FLAG (IP)

Full-length and uncropped western blot for Extended Data Figure 7

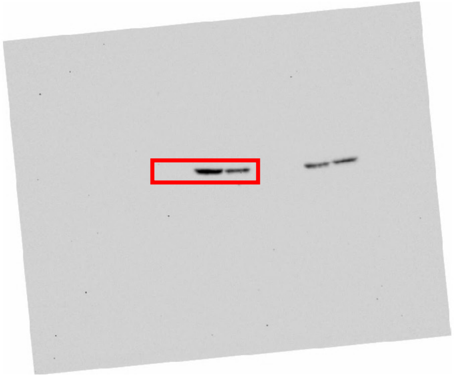

Figure 7b Myc (Input)

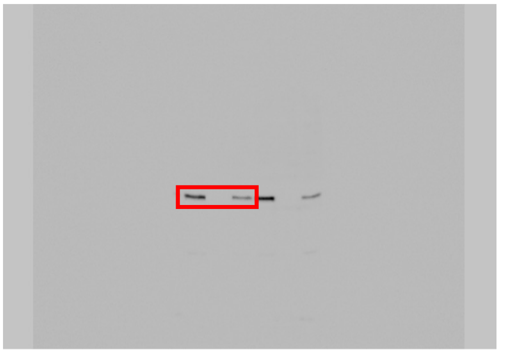

Figure 7b FLAG (Input)

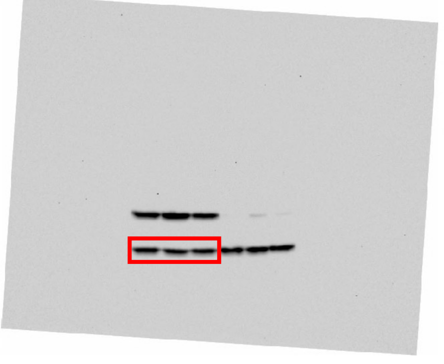

Figure 7b β-actin

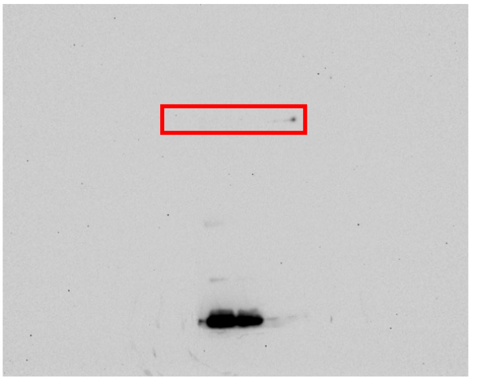

Figure 7c GST (IP)

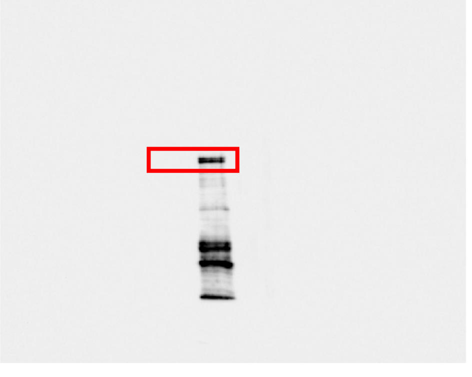

Figure 7c GST (input)

Full-length and uncropped western blot for Extended Data Figure 7

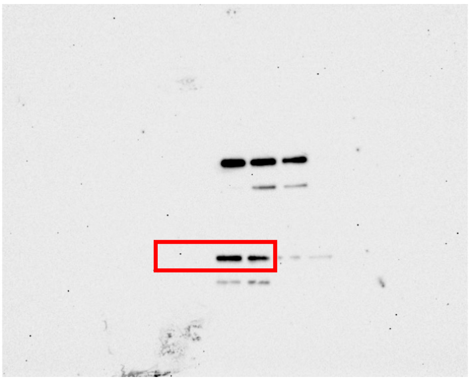

Figure 7e Myc (IP)

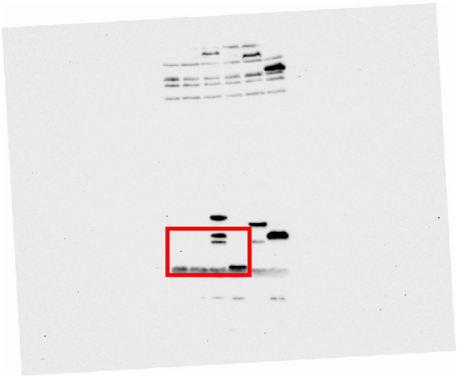

Figure 7e FLAG (IP)

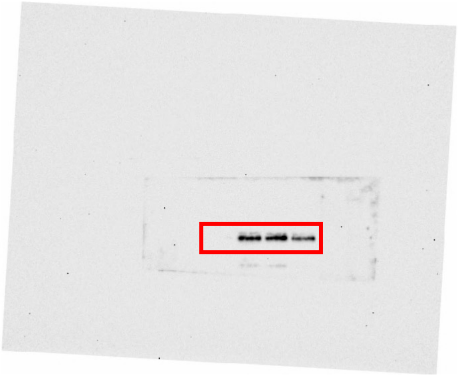

Figure 7e Myc (Input)

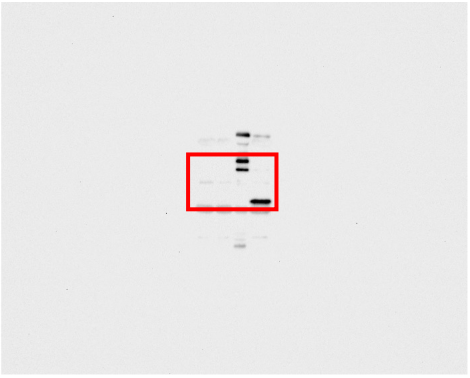

Figure 7e FLAG (Input)

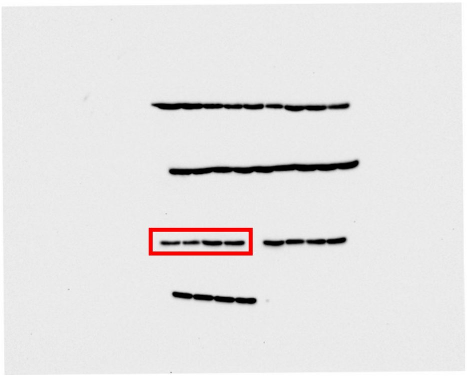

Figure 7e β-actin

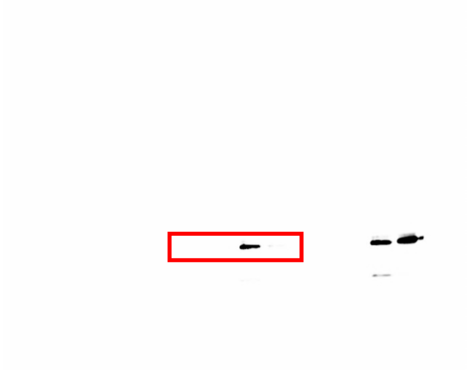

Figure 7f Myc (IP)

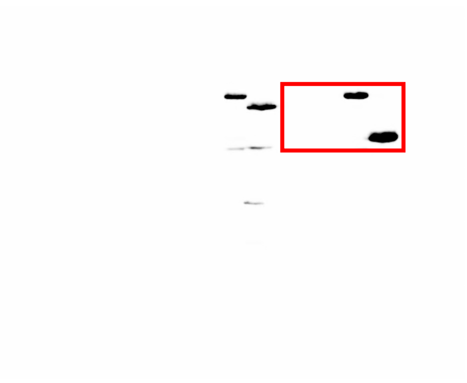

Figure 7f FLAG (IP)

Full-length and uncropped western blot for Extended Data Figure 7

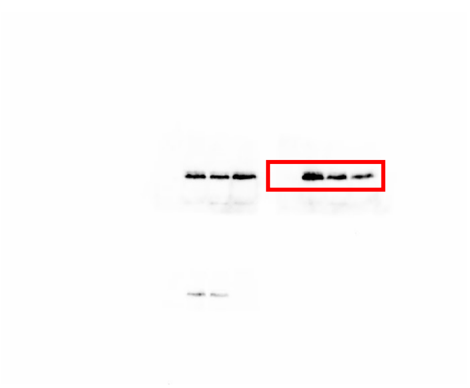

Figure 7f Myc (Input)

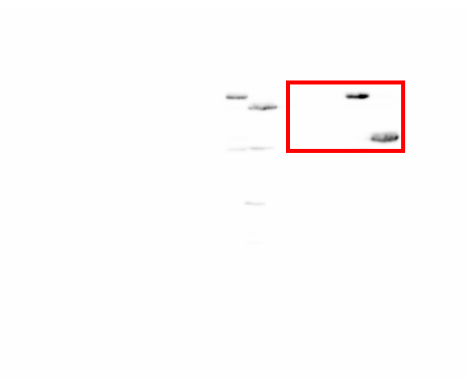

Figure 7f FLAG (Input)

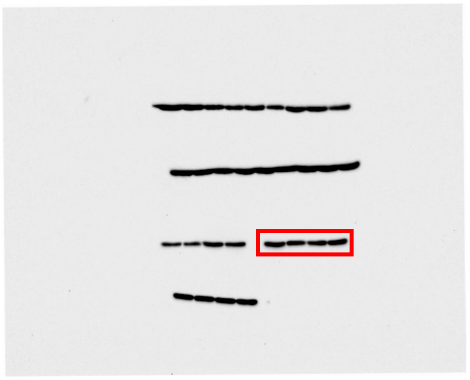

Figure 7f β-actin

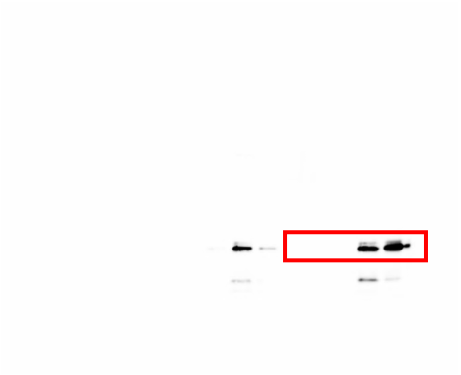

Figure 7g Myc (IP)

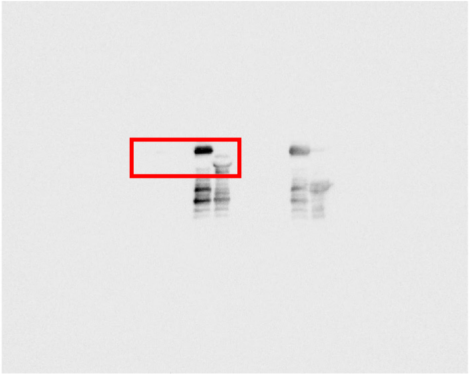

Figure 7g FLAG (IP)

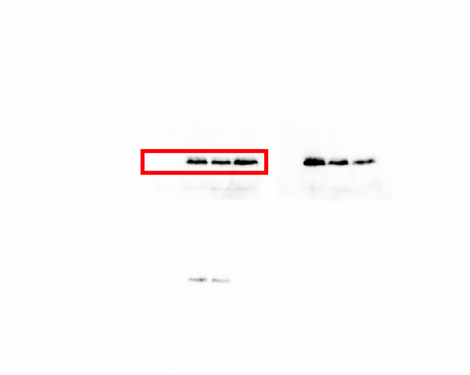

Figure 7g Myc (Input)

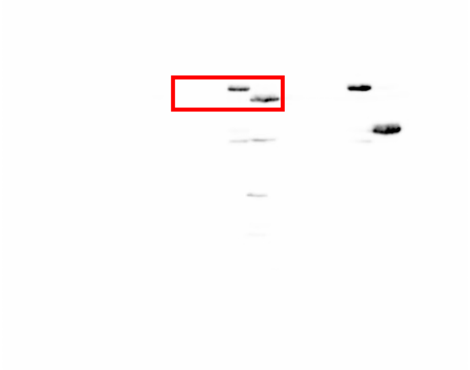

Figure 7g FLAG (input)

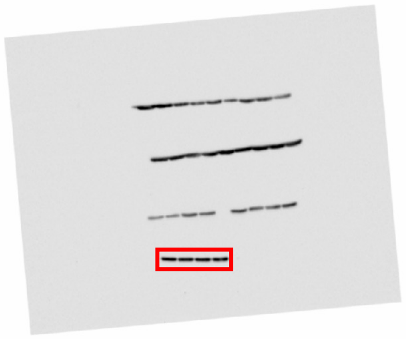

Figure 7g β-actin

Full-length and uncropped western blot for Extended Data Figure 8

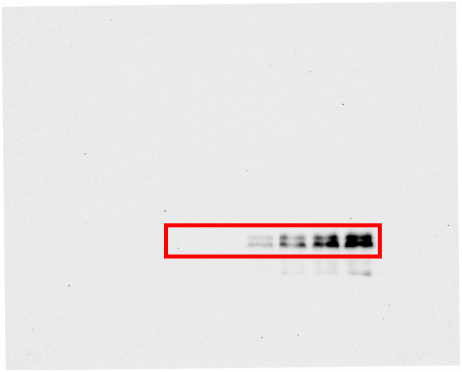

Figure 8a Myc (IP)

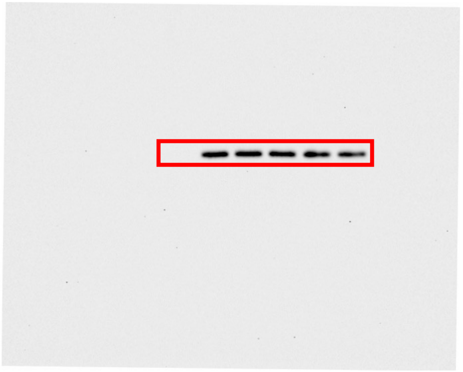

Figure 8a FLAG (IP)

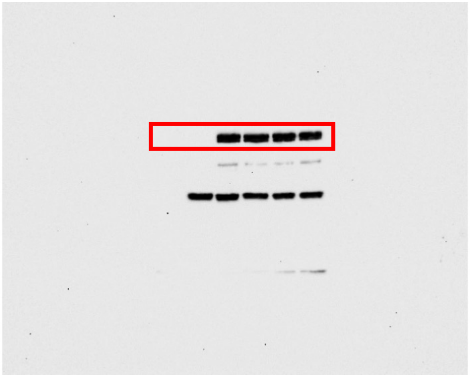

Figure 8a Myc (Input)

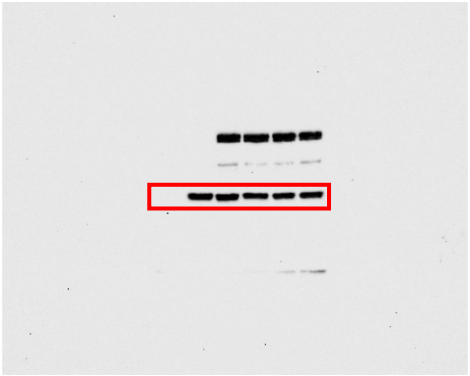

Figure 8a FLAG (Input)

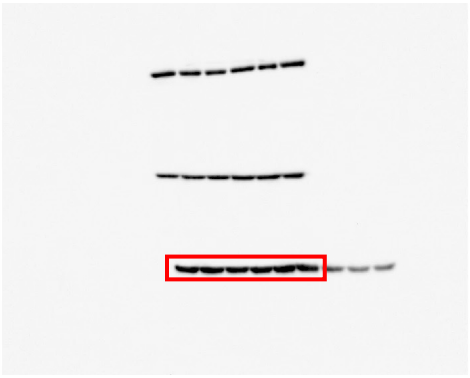

Figure 8a β-actin

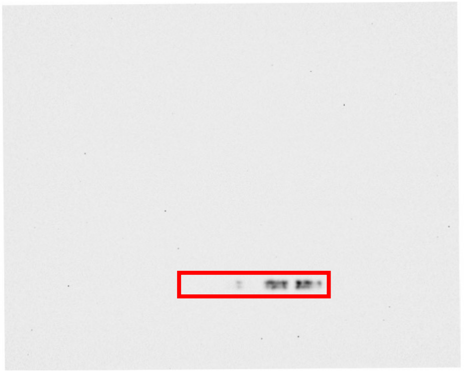

Figure 8b Myc (IP)

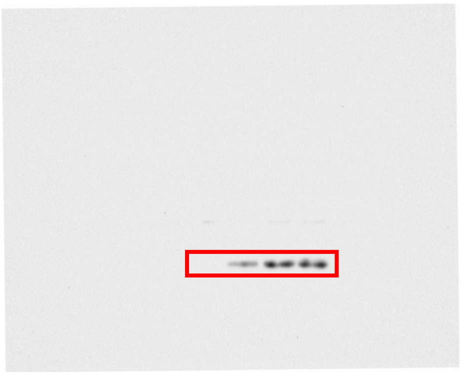

Figure 8b FLAG (IP)

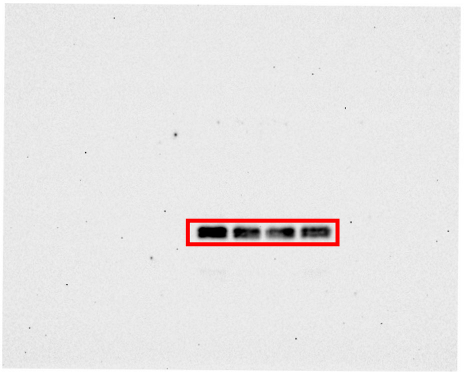

Figure 8b Myc (Input)

Full-length and uncropped western blot for Extended Data Figure 8

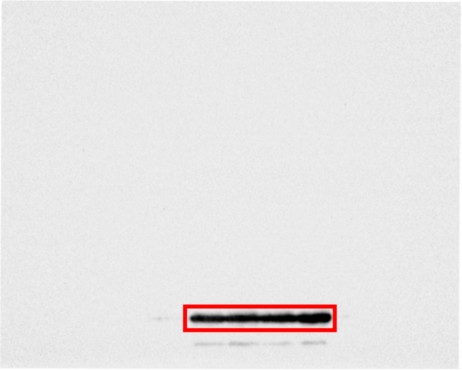

Figure 8b FLAG(Input)

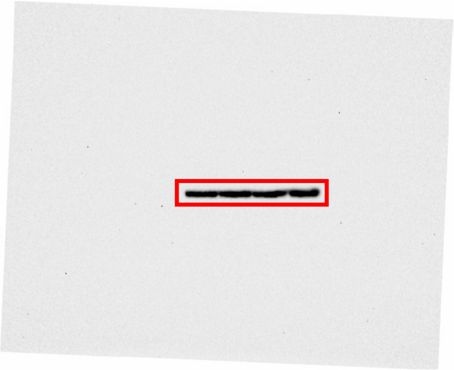

Figure 8b β-actin

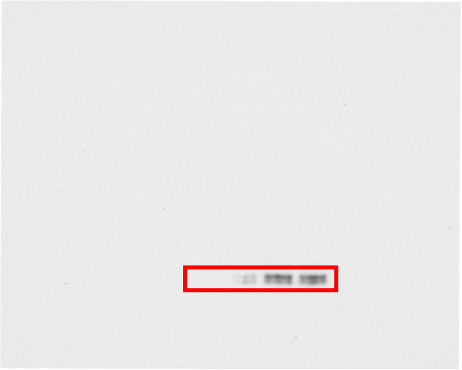

Figure 8c Myc (IP)

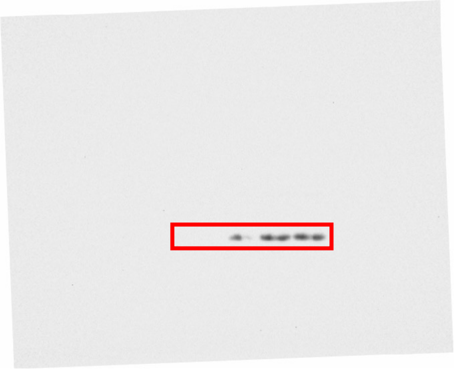

Figure 8c FLAG (IP)

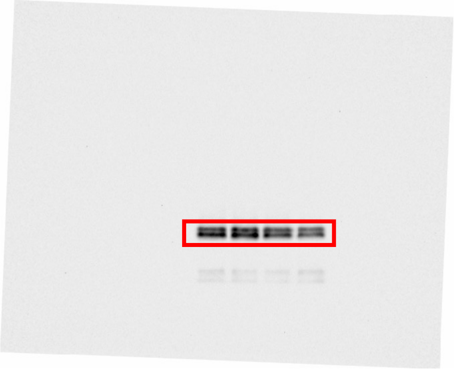

Figure 8c Myc (Input)

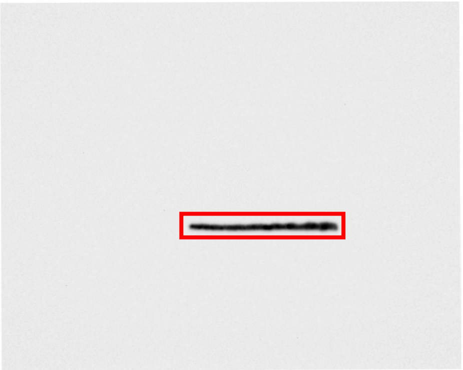

Figure 8c FLAG (Input)

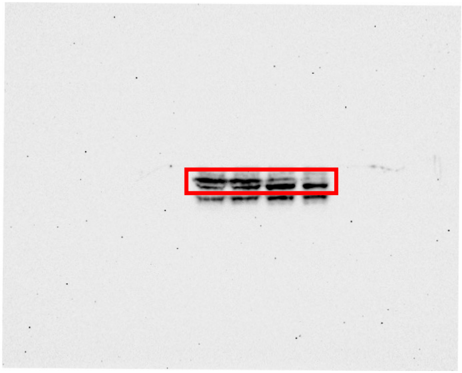

Figure 8c SIRT1

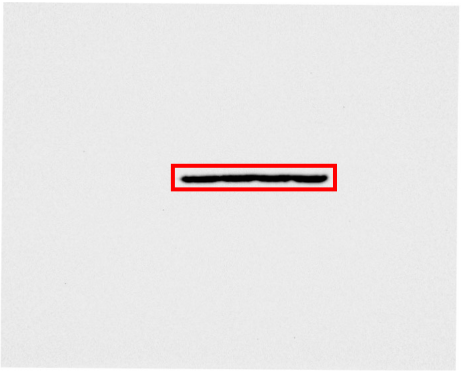

Figure 8c β-actin

Full-length and uncropped western blot for Extended Data Figure 8

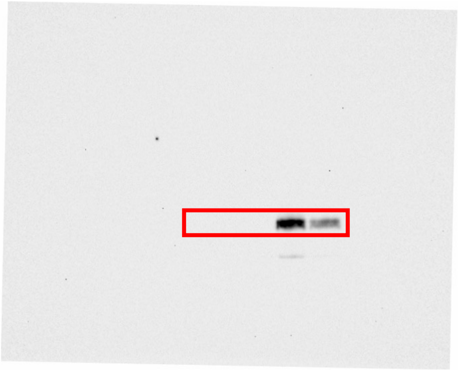

Figure 8d Myc (IP)

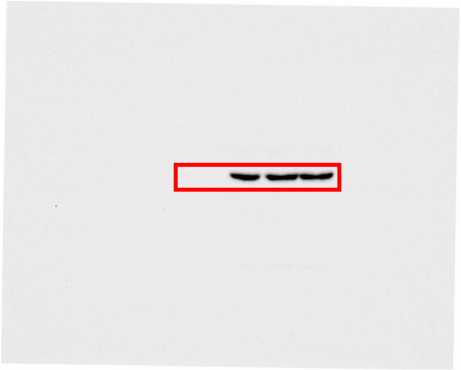

Figure 8d FLAG (IP)

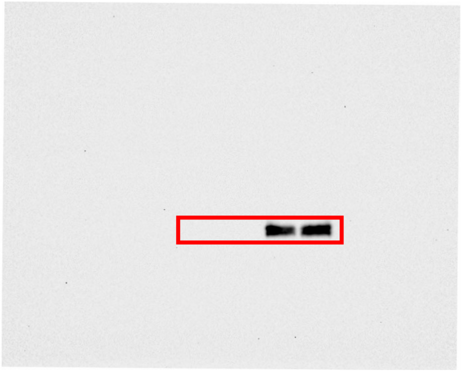

Figure 8d Myc (Input)

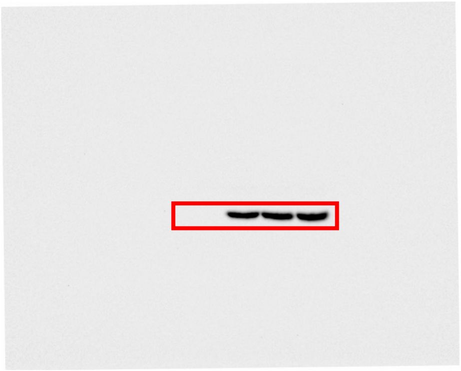

Figure 8d FLAG (Input)

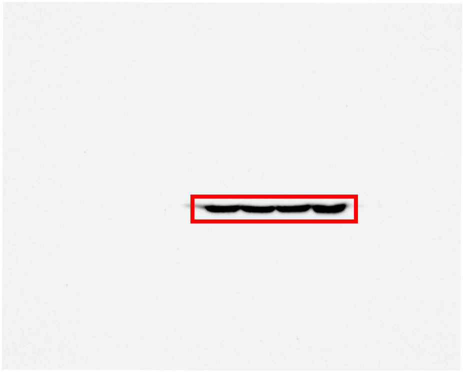

Figure 8d β-actin

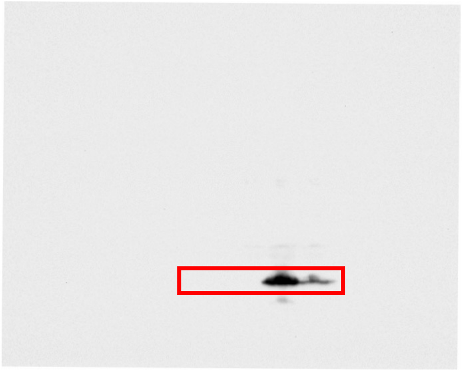

Figure 8e Myc (IP)

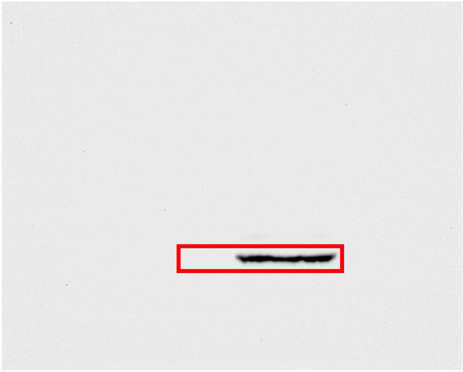

Figure 8e FLAG (IP)

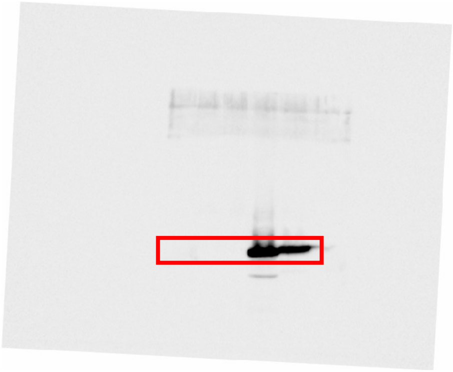

Figure 8e Myc (Input)

Full-length and uncropped western blot for Extended Data Figure 8

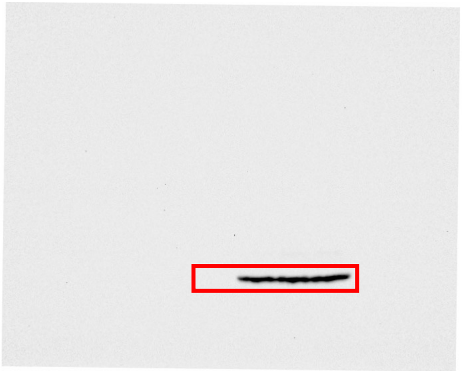

Figure 8e FLAG (Input)

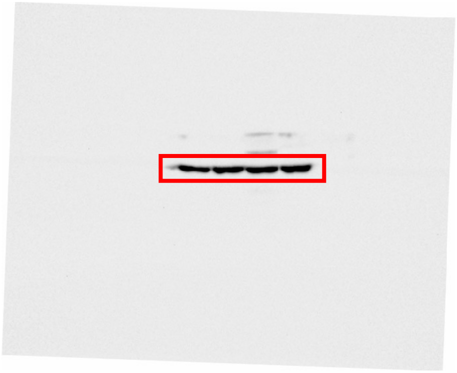

Figure 8e β-actin

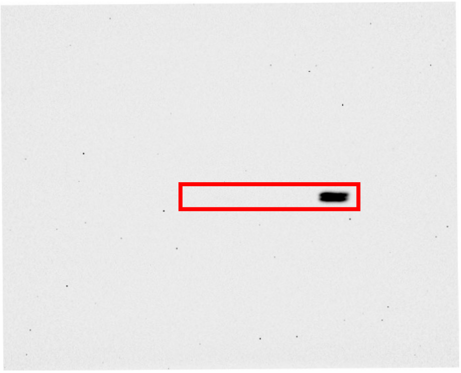

Figure 8f p53

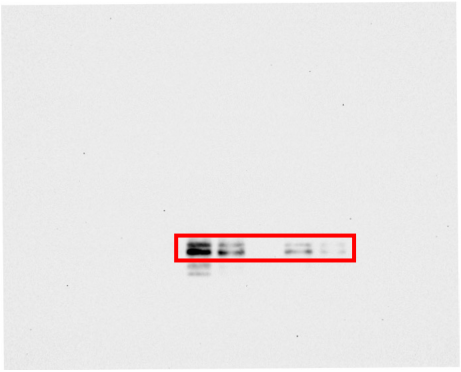

Figure 8f MDM2

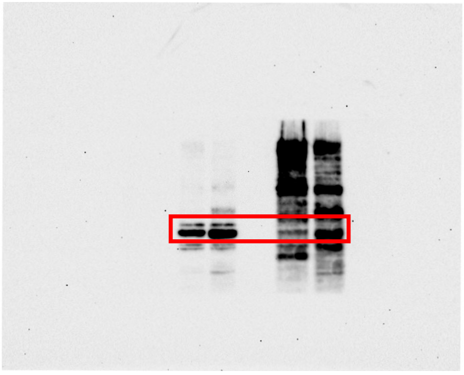

Figure 8f SIRT1

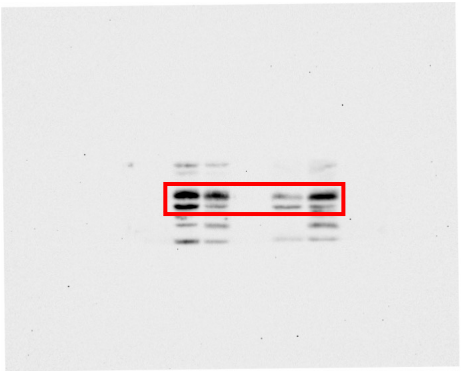

Figure 8f MKRN1

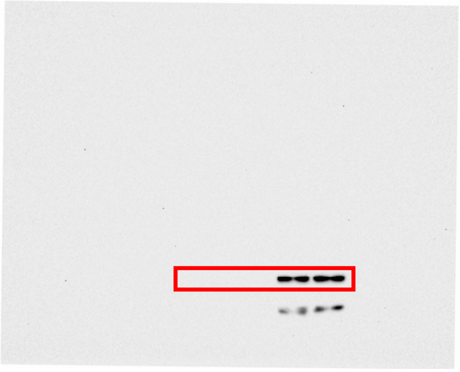

Figure 8f Lamin A

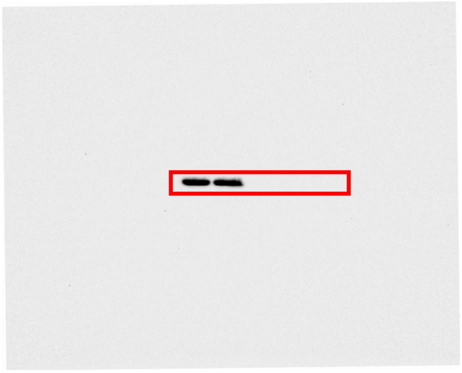

Figure 8f α-tubulin

Full-length and uncropped western blot for Extended Data Figure 9

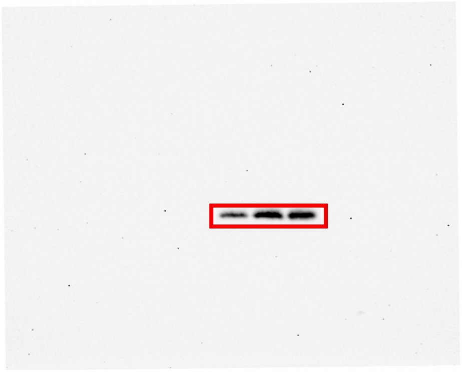

Figure 9a p21

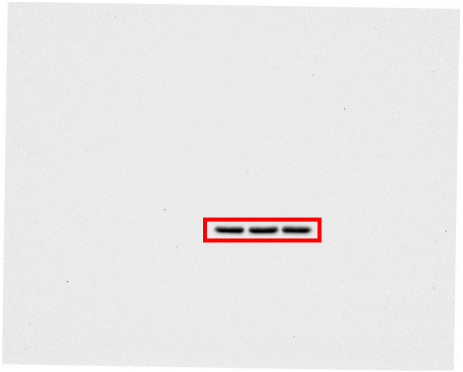

Figure 9a β-actin

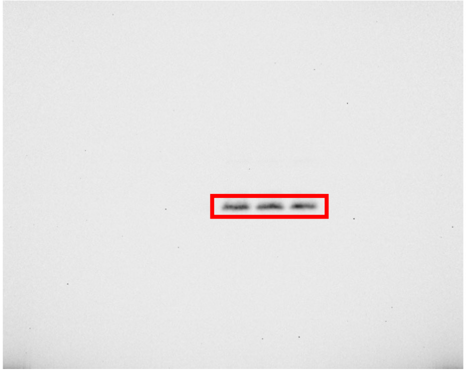

Figure 9b p21

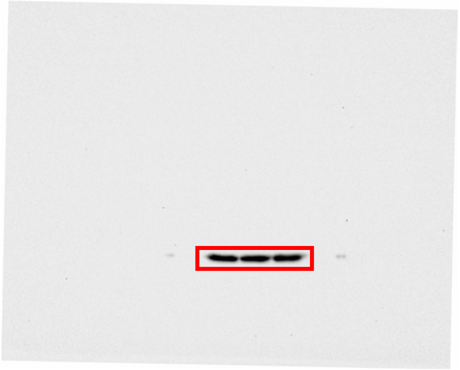

Figure 9b β-actin
